# Advances in TMDs-Based Electromagnetic Wave Absorbers: From Structural Engineering to Multicomponent Synergy

**DOI:** 10.1007/s40820-026-02266-w

**Published:** 2026-08-03

**Authors:** Yuefeng Yan, Anqi Lun, Boshi Gao, Yuhao Liu, Dechang Jia, Yu Zhou, Xiaoxiao Huang

**Affiliations:** 1 National Key Laboratory of Precision Welding & Joining of Materials and Structures, Harbin Institute of Technology, Harbin, 150001 People’s Republic of China; 2 School of Materials Science and Engineering, Harbin Institute of Technology, Harbin, 150001 People’s Republic of China; 3 Key Laboratory of Advanced Aerospace Structural Functional Materials and Preparation Technology, Ministry of Industry and Information Technology, Harbin Institute of Technology, Harbin, 150001 People’s Republic of China; 4 College of Aeronautical Engineering, Civil Aviation University of China, Tianjin, 300300 People’s Republic of China

**Keywords:** Electromagnetic wave absorption, Transition metal dichalcogenides, Structure regulation, Multicomponent strategy

## Abstract

Transition metal dichalcogenides (TMDs) have garnered considerable attention as advanced electromagnetic wave absorption (EMA) materials due to their unique layered structures, tunable electronic properties, and intrinsic defect-induced polarization mechanisms. This comprehensive review systematically summarizes recent advances in TMDs-based absorbers, with particular focus on two fundamental development strategies. The first involves multiscale structural design of pure phase TMDs spanning from atomic to submillimeter dimensions, achieved through precise defect regulation, phase engineering, and sophisticated morphological manipulation to optimize electromagnetic parameters and attenuation capabilities. The second strategy focuses on constructing multicomponent composite systems, incorporating dielectric matrices, magnetic elements, and multicomponent hybrids to achieve synergistic enhancement through interfacial polarization, conductive loss, and magnetic dissipation mechanisms. The review critically analyzes pioneering research achievements across various subfields while identifying specific challenges and opportunities within each domain. Future perspectives highlight emerging frontiers including atomic level interface engineering, inverse design of multicomponent and multiscale architectures, sustainable large-scale synthesis techniques, and development of multifunctional smart-response systems. This work aims to establish fundamental principles and provide forward-looking guidance for designing next-generation high-performance TMDs-based EMA materials with tailored functionalities.

## Highlights

- Systematically elucidated the core advantages of transition metal dichalcogenides (TMDs) as dielectric electromagnetic wave absorbing materials, emphasizing their multiscale tunability in both composition and structure.
- Provided a comprehensive overview of recent advances in pure-phase TMDs and their multicomponent composites, highlighting their critical role in electromagnetic wave absorption.
- Integrated the latest multidisciplinary breakthroughs of TMDs in frontier research areas to outline diverse future directions and potential for TMDs-based electromagnetic wave absorbing materials.

## Graphical abstract

**Figure:**
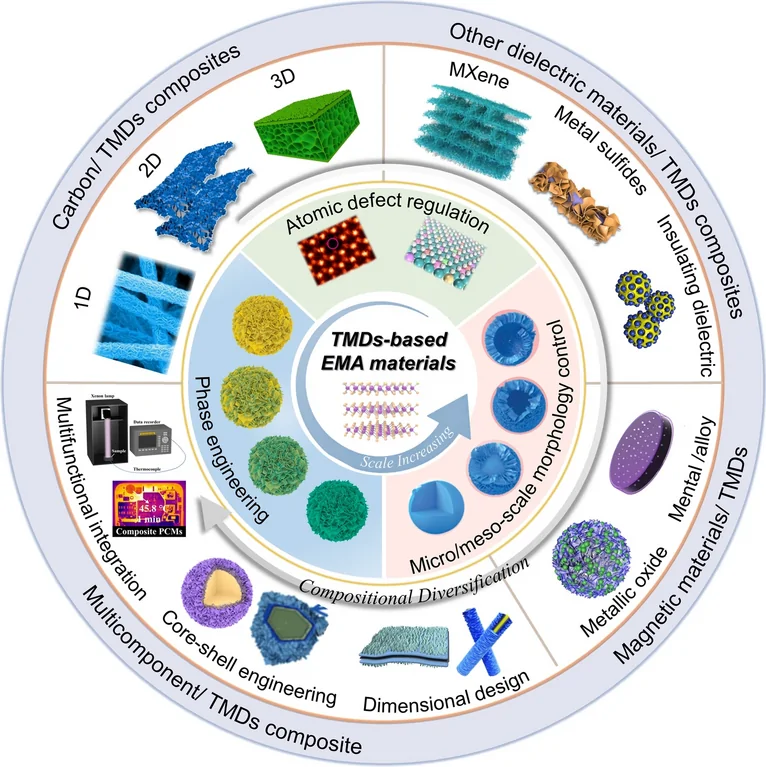

## Introduction

The exponential growth of wireless technologies, spanning 5G/6G infrastructure, tactical military communications, and satellite constellations, has precipitated an invisible crisis of electromagnetic pollution [1–6]. This proliferation generates disruptive interference in medical implants, aviation systems, and defense electronics, while long-term radiation exposure raises public health concerns. Conventional shielding solutions relying on metal enclosures merely reflect electromagnetic wave (EMW), exacerbating secondary pollution and failing critical stealth requirements in aerospace and defense applications [7–10]. Consequently, electromagnetic wave absorbing (EMA) materials capable of dissipating incident radiation as thermal energy through intrinsic loss mechanisms have become technologically indispensable [11–13]. In response to increasingly intricate electromagnetic environments, next‑generation EMA materials are required to simultaneously achieve thinner profiles, broader absorption bandwidths, lighter weight, and stronger attenuation. This set of demands is particularly challenging because traditional absorbers struggle to balance impedance matching with high loss across wide frequency ranges [14–18].

Transition metal dichalcogenides (TMDs), exemplified by MoS_2_ and WS_2_, have emerged as attractive candidates for meeting these multifaceted demands, owing to inherent structural and electronic advantages that directly address the limitations of conventional absorbers [19]. The advantageous features for TMDs-based EMWA materials are outlined below: (1) their two-dimensional (2D) layered architecture provides atomically thin confinement with micrometer-scale lateral dimensions, achieving superior specific surface areas [20]. This unique 2D structural characteristic creates abundant edge/defect sites and establishes conductive network pathways [21]. Concurrently, it amplifies EMW multi-reflection and scattering, and significantly prolongs EMW propagation trajectories, collectively boosting energy dissipation efficiency. These attributes enable lighter weight and broader bandwidth without sacrificing mechanical integrity [22]. (2) Polymorphic phase diversity, notably semiconducting 2H and metallic 1T phases in MoS_2_, enables dynamic conductivity tuning for optimal impedance matching and attenuation capacity across microwave spectra [23]. This directly contributes to thinner profiles and stronger absorption. (3) Electronic property tenability through layer number control or doping further modulates bandgaps and charge densities, creating tailored pathways for electron hopping and dipolar relaxation [24, 25]. This characteristic offers additional degrees of freedom for bandwidth broadening and loss tuning. (4) Intrinsic chemical compatibility facilitates hybridization with carbon networks, magnetic species, and conductive polymers, establishing multi-interfacial architectures where interfacial polarization, magnetic resonance, and conductive loss synergistically enhance broadband attenuation [26]. This further strengthens the ability to achieve high performance at low filler loadings.

This convergence of advantages has catalyzed two foundational strategies in recent TMDs-based absorber research. (1) Structural engineering on pure TMDs orchestrates defect engineering, phase transition, and micromorphology and hierarchical structure design across atomic to mesoscopic scales. Atomic vacancy generation and heteroatom doping induce localized dipoles in MoS_2_ lattices, amplifying Debye relaxation loss at microwave frequency [27, 28]. Controlled phase transitions between 2H and 1T phase configure electron transport networks critical for conductive dissipation [29]. Morphological modifications at the microscale substantially modulate dielectric properties and electrical characteristics, thereby enhancing EMW scattering and significantly improving absorption performance [30]. This structural control mechanism amplifies energy dissipation through tailored interactions at the material interface level. Hierarchical structure design of porous aerogels further extends EMW propagation paths to enhance wave–matter interactions through recurrent scattering. (2) Complementarily, multicomponent integration constructs heterostructured absorbers where hierarchical interfaces establish cooperative loss mechanisms [31]. Multicomponent engineering significantly expands the parametric design space for advanced materials, enabling synergistic integration of intrinsic properties from constituent components. This strategy allows precise tuning of dielectric behavior and electrical characteristics through controlled phase interactions. Furthermore, coupling with magnetic compounds imparts foreign magnetic dissipation capabilities to intrinsically nonmagnetic TMDs. Simultaneously, heterogeneous interfaces formed between dissimilar components facilitate intensive interfacial polarization via multiscale dipole rearrangement [32]. Beyond electromagnetic optimization, this approach further introduces unprecedented functionalities to TMDs-based systems, including infrared stealth and mechanical reinforcement, thereby accelerating the development of multifunctional EMA materials for next-generation applications.

**Fig. 1:**
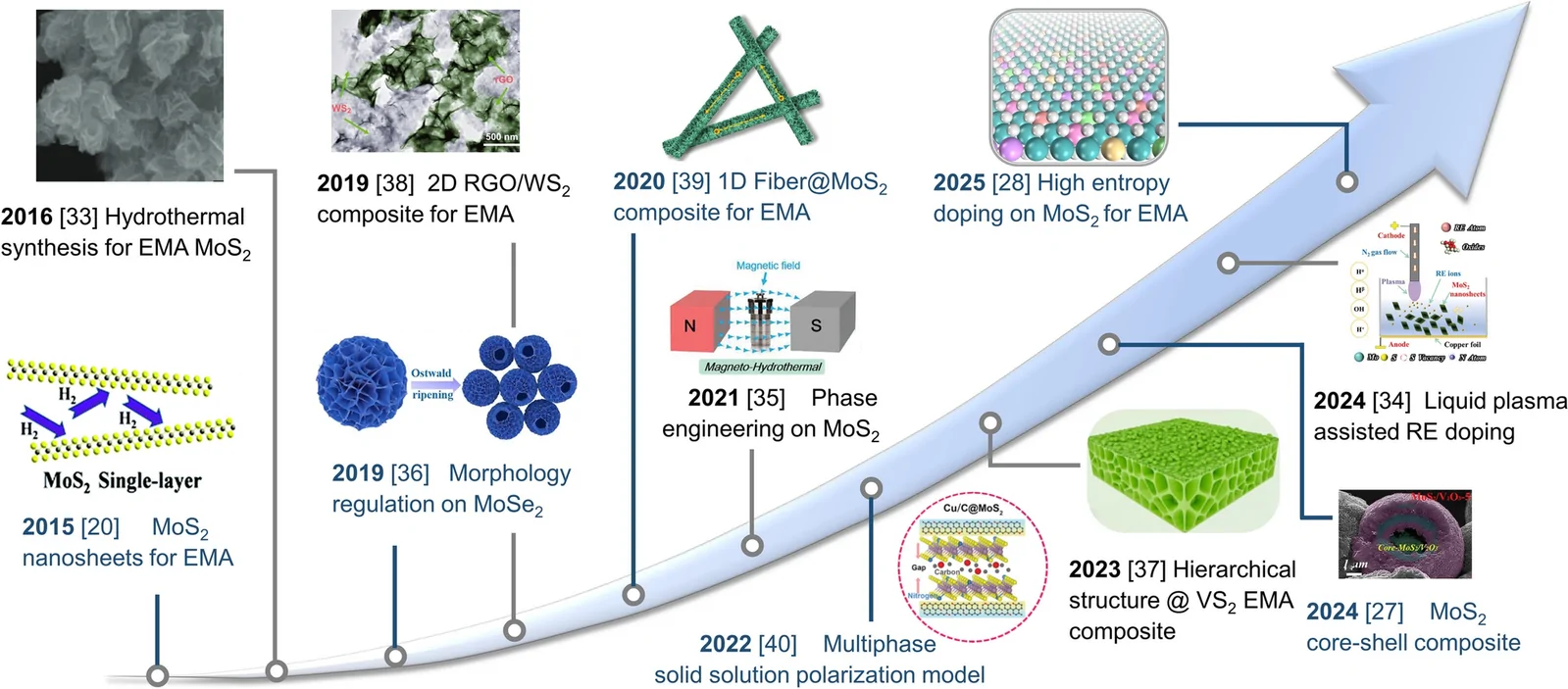
Timeline of development and major milestones in TMDs-based EMA materials. Reproduced with permission from Refs. [20, 27, 28, 33–40]

Some significant breakthroughs in TMDs-based EMA materials at key milestones are shown in Fig. 1. The outstanding EMA properties of MoS_2_ nanosheets were first revealed in 2015 [20]. Then, the hydrothermal method was introduced and became the most commonly used synthesis approach for TMDs-based EMA materials [33]. Subsequently, extensive research has been conducted along two main lines: multi-scale compositional and structural regulation of pure TMDs, and the design of TMDs-based multicomponent composites. Examples include doping effects [28, 34], phase engineering [35], and morphology control [36] of TMDs, as well as typical TMDs-based composites featuring different dimensions [27, 37–40]. This review first provides a comprehensive overview of the composition, structural features, and electronic properties of TMDs, elucidating their fundamental connections with electromagnetic behavior. We then analyze how multiscale structural modifications, spanning atomic-scale doping/defects, phase engineering, and micro/meso-scale morphology control, collectively optimize EMA performance. Furthermore, we highlight innovative design approaches for multicomponent TMDs composites, such as integration with dielectric matrices, magnetic materials, and conductive polymers. Through a critical comparison of recent breakthroughs in TMDs-based absorbers, we delineate current research trends and unresolved challenges. The review concludes by outlining actionable future pathways to enhance the functionality and applicability of TMDs-based EMA systems.

## Fundamental Mechanisms of TMDs-based EMW Materials

The interaction between materials and electromagnetic fields is characterized by their electromagnetic parameters, namely the complex relative permittivity (*ε*_*r*_ = *ε′* *−* *jε"*) and complex relative permeability (*μ*_*r*_ = *μ′* *−* *jμ"*). It is widely accepted that the real part of permittivity (*ε′*) reflects the material's ability to store electric field energy, while the imaginary part (*ε"*) represents its capacity to dissipate electric field energy [41]. Similarly, the real part of permeability (*μ′*) indicates magnetic energy storage, and the imaginary part (*μ"*) corresponds to magnetic energy dissipation [42, 43]. When EMW impinges on a material surface, it splits into incident, transmitted, and reflected components. In the context of EMA materials based on the metal-backing model, where the absorber is coated onto a perfect electric conductor, all transmitted waves are reflected back into the absorber for secondary absorption. Optimal EMA performance requires two critical factors: excellent impedance matching and sufficient attenuation capability. The former requires that the wave impedance of the material closely matches that of free space, allowing maximal wave penetration into the material rather than surface reflection. The latter demands efficient conversion of incident electromagnetic energy into thermal dissipation. However, extensive literature demonstrates that these two properties are generally mutually contradictory. Materials with good impedance matching often exhibit weak attenuation, while strongly lossy materials frequently suffer from impedance mismatch [44–46]. Thus, reconciling this inherent trade-off remains a fundamental challenge in the design of high-performance microwave absorbers.

### Impedance Matching Characteristic

The intrinsic wave impedance of a material and its matching characteristics with free space can be quantitatively described by the following equation [4, 47]:

1 $$Z = \left| {\frac{{Z_{r} }}{{Z_{0} }}} \right|$$

2 $${\mathrm{Z}}_{r} = \sqrt {\frac{{\mu_{r} }}{{\varepsilon_{r} }}} , \; {\mathrm{Z}}_{0} = \sqrt {\frac{{\mu_{0} }}{{\varepsilon_{0} }}}$$

where *Z*_*r*_, Z_0_, *ε*_0_*, μ*_0_ represent the material’s intrinsic impedance, the impedance of free space, the vacuum permittivity and vacuum permeability, respectively. When the value of *Z* approaches to 1, it indicates excellent impedance matching. This formulation explicitly demonstrates that achieving optimal impedance matching requires precise control over the material's electromagnetic parameters, particularly through careful balancing of permittivity and permeability.

In practical applications involving metal-backed absorbers, the input wave impedance (*Z*_in_) of the absorber material is more widely recognized, as defined by the subsequent equation [48]:

3 $$Z_{{{\mathrm{in}}}} = Z_{0} \sqrt {\frac{{\mu_{r} }}{{\varepsilon_{r} }}} {\mathrm{tanh}}\left( {j\frac{2\pi fd}{{\mathrm{c}}}\sqrt {\mu_{r} \varepsilon_{r} } } \right)$$

where *f* and c represent the frequency of the EMW and the speed of light in vacuum. d is the thickness and of the absorber. This parameter comprehensively accounts for both the primary reflection at the absorber's front surface and the secondary reflection occurring at the absorber-metal interface. A value of *Z*_in_ approaching 1 signifies that the material has achieved superior impedance matching performance. Impedance matching efficacy could also be quantifiable through the Δ function more visually described as following [49]:

4 $$\Delta = \left| {\sinh^{2} \left( {Kfd} \right) - M} \right|$$

5 $$K = \frac{{4\pi \sqrt {\varepsilon^{\prime } \mu^{\prime } } }}{{c \cdot \cos \delta_{\varepsilon } \cos \delta_{\mu } }}\sin \frac{{\delta_{\varepsilon } + \delta_{\mu } }}{2}$$

6 $$M = \frac{{4\varepsilon^{\prime } \cos \delta_{\mu } \mu^{\prime } \cos \delta_{\varepsilon } }}{{\left( {\mu^{\prime } \cos \delta_{\varepsilon } - \varepsilon^{\prime } \cos \delta_{\mu } } \right)^{2} + \left( {\tan \frac{{\delta_{\mu } - \delta_{\varepsilon } }}{2}} \right)^{2} \left( {\mu^{\prime } \cos \delta_{\varepsilon } + \varepsilon^{\prime } \cos \delta_{\mu } } \right)^{2} }}$$

where $\delta_{\varepsilon } = \arctan \frac{{\varepsilon^{\prime \prime } }}{{\varepsilon^{\prime } }}$ and $\delta_{\mu } = \arctan \frac{{\mu^{\prime \prime } }}{{\mu^{\prime } }}$. Δ values asymptotically approaching zero indicate maximal EMW penetration. Achieving effective EMW attenuation (i.e., ≥ 90% absorption, RL ≤ −10 dB) necessitates Δ values below the critical threshold of 0.4. This performance boundary represents a fundamental constraint in EMA material design, where Δ function minimization governs interfacial impedance compatibility. This condition demands synergetic engineering of dielectric dissipation and magnetic loss characteristics within the absorber architecture.

### Attenuation Capacity and EMW Loss Mechanism

Following successful EMW penetration into the material matrix, sufficient attenuation capability is essential to efficiently convert incident wave energy into thermal dissipation. This energy transformation process is quantitatively governed by the attenuation constant (*α*), which comprehensively characterizes the material's electromagnetic energy decay potential as described in Eq. (7). Current methodologies routinely deconstruct the EMW attenuation capability into dielectric loss and magnetic loss components for discrete investigation of polarization and magnetization response mechanisms [50].

7 $$\alpha = \frac{{\sqrt 2 {\uppi }f}}{{\mathrm{c}}} \times \sqrt {\left( {\mu^{\prime \prime } \varepsilon^{\prime \prime } - \mu^{\prime } \varepsilon^{\prime } } \right) + \sqrt {\left( {\mu^{\prime \prime } \varepsilon^{\prime \prime } - \mu^{\prime } \varepsilon^{\prime } } \right)^{2} + \left( {\mu^{\prime } \varepsilon^{\prime\prime} + \mu^{\prime \prime } \varepsilon^{\prime } } \right)^{2} } }$$

#### Dielectric Loss Mechanism

The alternating electric field component of EMW interacts with charges in materials, inducing two distinct effects: charge migration and dipole polarization [51]. Charge migration, which relies on excellent electrical conductivity, generates currents that convert electromagnetic energy into Joule heat through conduction loss. Some phases of TMDs exhibit metallic properties, such as 1T MoS_2_, which can provide strong conductive loss and thus ensure the EMW dissipation capability of TMDs-based absorbers [35]. While conduction loss provides strong EMW attenuation capacity and is commonly observed in various absorber materials, excessive microcurrents can produce opposing electric fields that re-radiate EMW, leading to significant impedance mismatch. Consequently, although conduction loss efficiently dissipates electromagnetic energy, it is not an ideal attenuation mechanism and must be carefully controlled within certain limits. In contrast, dipole polarization depends on inherent dipoles within the material system, which may originate from polar functional groups, intrinsic crystal dipole moments, heteroatoms, vacancies, grain boundaries, or heterogeneous interfaces [52]. Polarization loss is less constrained by impedance mismatch limitations, making it a more desirable EMW attenuation mechanism. For TMDs-based materials, dipole polarization loss mainly originates from intrinsic defects, such as S vacancies, edge sites with dangling bonds, intercalated ions or molecules, and interfaces. These structural imperfections break local charge symmetry and create polarized centers, which respond to alternating electromagnetic fields and contribute to dielectric loss [40]. The dipole polarization-relaxation process in dielectric materials is typically described by the Debye theory, as expressed in following equations [53]:

8 $$\varepsilon^{\prime } = \varepsilon_{\infty } + \frac{{\varepsilon_{0} - \varepsilon_{\infty } }}{{1 + \omega^{2} \tau^{2} }}$$

9 $$\varepsilon^{\prime \prime } = \varepsilon_{\infty } \frac{{\varepsilon_{0} - \varepsilon_{\infty } }}{{1 + \omega^{2} \tau^{2} }}\omega \tau$$

where *ω* = 2π*f* represents the circular frequency of EMW, *τ* represents the relaxation time of the electric dipole, *ε*_0_ and *ε*_∞_ represent the static permittivity, and relative permittivity at high-frequency limit, respectively. This formulation naturally explains the frequency dispersion phenomenon where the permittivity curves gradually decrease with increasing frequency. This behavior originates from the fact that dipoles within the material rotate in response to the applied alternating electric field, but their response becomes increasingly delayed as the EMW frequency rises, eventually leading to their diminished contribution to the permittivity.

Based on the Debye theory, the *ε*′−*ε*″ relationship can be derived as Cole–Cole model [54]:

10 $$\left( {\varepsilon^{\prime } - \varepsilon_{\infty } } \right)^{2} + \left( {\varepsilon^{\prime \prime } } \right)^{2} = \left( {\varepsilon_{0} - \varepsilon_{\infty } } \right)^{2}$$

The Cole–Cole semicircle serves as a powerful tool for analyzing dipole polarization phenomena in materials. Each semicircle represents a distinct Debye dipole relaxation process. However, since the polarization relaxation processes in real material systems often deviate from the ideal Debye model, various modified versions have been proposed to more accurately describe actual dipole polarization behaviors. For instance, researchers Davidson suggested that interfacial effects and other internal interactions can hinder dipole rotation, leading to the introduction of a correction factor *β* into the Debye polarization equation to refine the dielectric model as Eq. (11) [55, 56]. For TMDs-based EMA absorbers, the polarization relaxation effects induced by defects such as phase interfaces, nanosheet edges, and vacancies are more appropriately described by this model. However, to date, relatively few studies have been conducted on the microstructure dielectric polarization structure–property relationship in this context.

11 $$\varepsilon_{r} = \varepsilon^{\prime } - j\varepsilon^{\prime \prime } = \varepsilon_{\infty } + \frac{{\left( {\varepsilon_{0} - \varepsilon_{\infty } } \right)}}{{\left( {1 + j\omega \tau } \right)^{\beta } }},\; 0 < \beta \le 1$$

Furthermore, considering the presence of leakage currents in materials, conduction loss has also been incorporated into the modified Debye polarization model, as described by the following equation [57]:

12 $$\varepsilon^{\prime \prime } = \varepsilon_{p}^{\prime \prime } + \varepsilon_{c}^{\prime \prime } = \frac{{\varepsilon_{0} - \varepsilon_{\infty } }}{{1 + \omega^{2} \tau^{2} }}\omega \tau + \frac{\sigma }{{\omega \varepsilon_{0} }}$$

where $\varepsilon_{p}^{\prime \prime }$, $\varepsilon_{c}^{\prime \prime }$, and *σ* represent polarization loss part, and conductive loss part and conductivity, respectively. This comprehensive model is widely employed by researchers to separate and quantify the polarization loss and conduction loss components, thereby enabling in-depth analysis of the dielectric loss mechanisms in materials [58, 59].

#### Magnetic Loss Mechanism

The magnetic dissipation mechanisms in microwave absorbers primarily encompass four distinct phenomena: domain wall resonance, natural ferromagnetic resonance, eddy current effects, and hysteresis loss. Hysteresis loss arises from irreversible magnetization processes under weak applied fields, while domain wall resonance typically manifests in multi-domain systems at ultralow frequencies (MHz range). Consequently, neither mechanism significantly contributes to magnetic loss in the microwave frequency regime. Contemporary understanding identifies eddy current loss and natural ferromagnetic resonance as the dominant magnetic dissipation pathways in this spectral region [60, 61]. When subjected to alternating magnetic fields, magnetic materials generate circulating currents known as eddy currents. Unlike conventional conductive loss, this phenomenon represents an energy conversion process from magnetic to electrical energy. The magnitude of eddy current loss could be described by the following relationship [62, 63]:

13 $$C_{0} \approx \frac{{2\pi \mu_{0} }}{3}\mu^{\prime 2} \sigma D^{2} f = \mu^{\prime \prime } \left( {\mu^{\prime } } \right)^{ - 2} f^{ - 1}$$

In principle, *C*_0_ should remain frequency-independent if solely attributable to eddy current effects. In general, TMDs are nonmagnetic or weakly magnetic materials, and their permeability signals in the microwave frequency range are almost negligible. Therefore, the magnetic loss in TMDs-based absorbers typically originates from the magnetic components added to the composites, rather than from the TMDs themselves. For this reason, the magnetic loss mechanism is not further discussed in this paper.

### Characterization Techniques for the EMA Performance

The EMA performance of materials is typically characterized by reflection loss (RL) metrics. When RL values fall below −10 dB, effective absorption is achieved, with the corresponding frequency range defining the effective absorption bandwidth (EAB). The strongest absorption intensity of a certain material is quantified by the minimum reflection loss value (RL_min_). Consequently, advanced EMA materials seek to maximize both EAB and RL_min_. Commonly, EMW absorption performance can be characterized by either direct (arc method and far field RCS method), or indirect method (coaxial-line and waveguide method).

#### Direct Method

The direct method mainly includes the arch method and the far field RCS method [64, 65]. These approaches directly measure the suppression of EMW signals reflected from a metal backplane covered by the EMA materials and can directly yield the reflection loss curve. By simulating or approximating the radar stealth effect of the absorber on metal components, the direct method provides the most accurate evaluation of the material's EMA performance. However, the direct method suffers from three major drawbacks. (1) It is expensive, particularly the far field RCS approach. (2) A large amount of absorbing material is required, and the sample must be fabricated as a complete absorbing layer whose size depends on the frequency range (see Table 1). (3) It does not provide intrinsic electromagnetic parameters such as complex permittivity and complex permeability, which are essential for revealing the EMA mechanisms. As a result, the direct method is not the first choice in most studies on EMA materials and is usually reserved for final performance verification.

**Table 1:** The test frequency ranges and sample sizes for different methods

Method	Length (mm)	Width (mm)	Thickness (mm)	Frequency (GHz)	Expense
Arch method	180	180		12–40	High
	300	300	Tunable	2–18	
	600	600		1–4	
Free-space Method	180	180	0.5–1	18–40	High
	300	300	1–5	2–18	
Waveguide method	7.2	3.6		26.5–40	Medium
	11.0	4.5		18–26.5	
	15.9	8.0	Tunable	12.4–18	
	22.9	10.2		8–12.4	
	34.7	15.8		5.38–8.2	
	47.2	22.0		3.95–6	

	Out diameter (mm)	Inner diameter (mm)	Thickness (mm)	Frequency (GHz)	
Coaxial-line method	7.0	3.04	2–4	2–18	Low

#### Indirect Method

The indirect method for EMA materials mainly includes the transmission reflection method (comprising the coaxial line and waveguide method) and the free space method. The transmission reflection method measures the S parameters of a sample filled in a transmission line and then retrieves the electromagnetic parameters, from which the reflection loss can be calculated based on transmission line theory (Eqs. 3 and 14) [66]. The transmission reflection method is the most convenient and widely used approach in the study of EMA materials. For powder material, it should be pressed into a specific shape via homogeneously mixing with EMW transparent matrix at a certain filling ratio. This method requires the sample to be tightly fitted to the fixture, which calls for high fabrication precision. Nevertheless, it consumes only a small amount of material and low cost, making it well suited for mechanism studies and materials screening, especially for TMDs-based EMA materials. The coaxial line method can obtain electromagnetic parameters in the range of 1–18 GHz and is the most convenient method. The waveguide method requires changing the fixture and sample size according to the frequency band, but it generally offers higher accuracy than coaxial line method. The free space method, on the other hand, uses antennas to transmit and receive EMW in free space through the sample to calculate *S* parameters. It is non-contact, which gives it a clear advantage for measuring samples at high temperatures, though the cost is relatively high [67].

14 $$RL\,\left( {{\mathrm{dB}}} \right) = 20{\text{ log}}\left| {\frac{{Z_{{{\mathrm{in}}}} - Z_{0} }}{{Z_{{{\mathrm{in}}}} + Z_{0} }}} \right|$$

## Microstructures and Properties of TMDs

### Composition and Structure

**Fig. 2:**
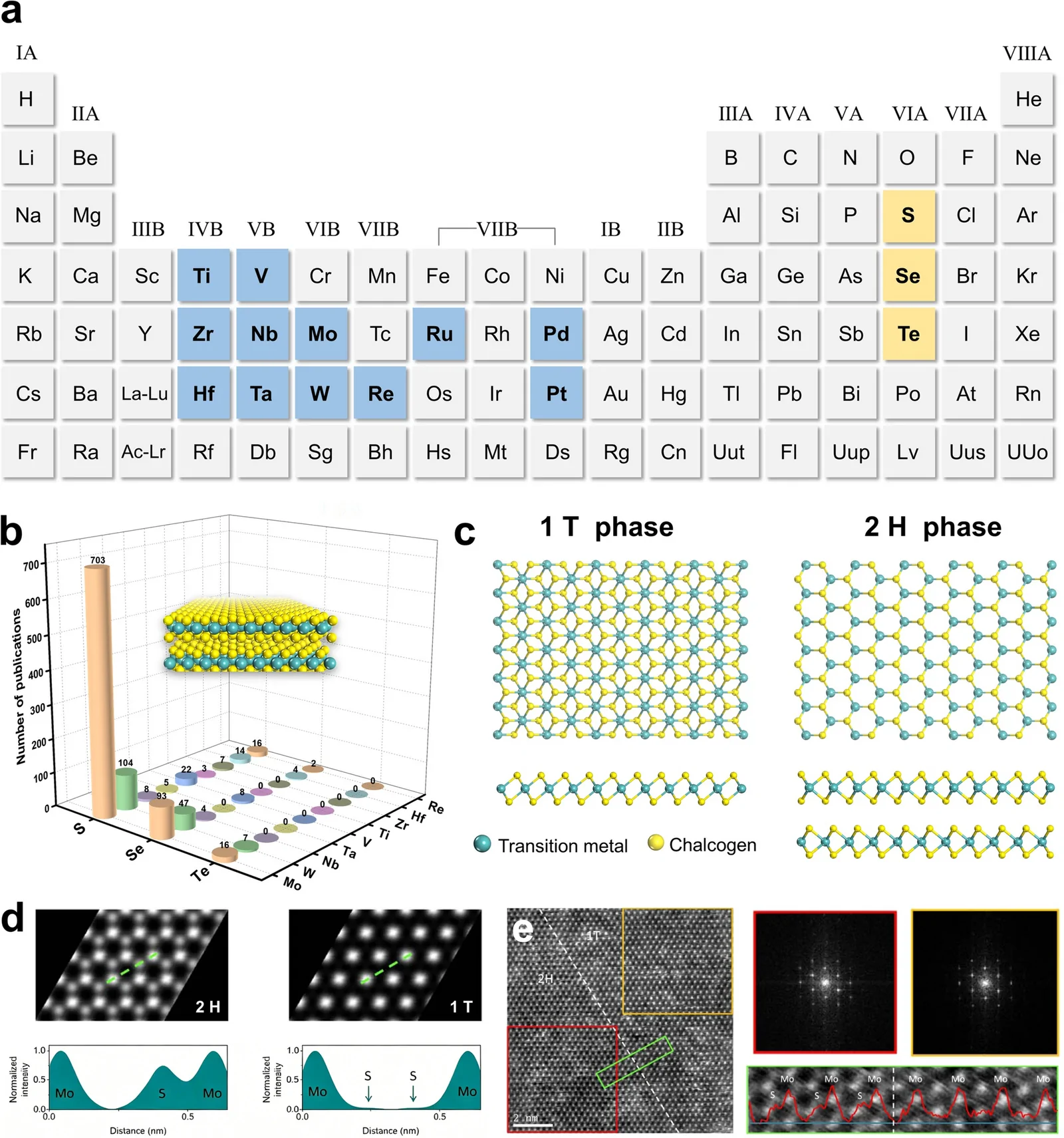
**a** Anion and cation element composition of layered TMDs; **b** number of publications of TMDs-based EMA materials with various element configurations (data collected from Web of Science); **c** atomic structure of TMDs in their typical 1T and 2H phase; **d** simulated HAADF-STEM images of ideal 2H-MoS_2_ and 1T-MoS_2_ phases. The bright spots correspond to Mo atoms; **e** STEM image of a region where a boundary between 2H and 1T phases and corresponding Fourier transform images (right top) and the intensity profile along the green region (right bottom) [72]. Copyright 2012 American Chemical Society

The family of 2D TMDs exhibits excellent cation–anion compatibility. The cationic components typically comprise transition metals from groups IVB to VIIIB, while the anionic counterparts include group VIA elements such as S, Se, and Te [68], as illustrated in Fig. 2a. This diverse elemental combination endows TMDs with a broad spectrum of tunable electronic and dielectric properties, which is directly relevant to EMW absorption because the dielectric response can be systematically adjusted by choosing different metal‑chalcogen pairs. Among them, MoS_2_ has attracted the most attention and research. Structurally, TMDs are characterized by a layered architecture featuring strong covalent bonding within individual layers and weak van der Waals interactions between adjacent layers. Each monolayer consists of a sandwich-like structure with a transition metal (M) atom covalently bonded to two chalcogen (S) atoms in an S-M-S configuration. The weak interlayer forces allow TMDs to be exfoliated into single-layer sheets or synthesized as 2D materials via bottom-up growth techniques [69]. This layered structure is particularly advantageous for EMA, as it facilitates multiple reflections between layers and prolongs the propagation path of incident EMW, thereby enhancing energy dissipation. The rich elemental diversity of TMDs has spurred extensive research into their use as EMW absorbers. As shown in Fig. 2b, current studies are predominantly focused on sulfides and selenides of the VIB group. In contrast, TMDs based on VB or IVB transition metals, as well as tellurides, remain significantly less explored, presenting promising avenues for future research.

Depending on the coordination environment of the M atoms and the stacking sequence of the layers, TMDs can adopt various polymorphic phases, including 1T, 1T′, 2H, and 3R [70]. Among these, the 1T and 2H phases (Fig. 2c) are the most extensively studied in the context of EMA field [71]. The metallic 1T phase exhibits high conductivity, which enhances conduction loss, whereas the semiconducting 2H phase provides better impedance matching; their coexistence creates abundant heterophase interfaces that promote interfacial polarization. The 1T phase exhibits octahedral coordination, where the S atoms in adjacent layers do not align along the *z*-axis, and featuring a single-layer periodicity. In contrast, the 2H phase features trigonal prismatic coordination, with sulfur atoms in consecutive layers perfectly overlapping, forming a two-layer repeating unit.

This structural distinction leads to pronounced differences in their high-angle annular dark-field scanning transmission electron microscopy (HAADF-STEM) images. As demonstrated in Fig. 2d, the simulated STEM signals reveal that in 1T-MoS_2_, the S atoms (with an atomic number much lower than Mo) remain undetectable (contributing only 3% of the Mo signal intensity), leaving only isolated triangular arrangements of Mo atoms visible. Conversely, in the 2H phase, the superposition of S atoms from upper and lower layers enhances their signal intensity to 74% of the Mo signal, producing a distinct honeycomb pattern [72]. These characteristic HAADF-STEM signatures enable clear differentiation between 1T and 2H domains in MoS_2_ monolayers, as well as the identification of 1T–2H heterophase interfaces, as shown in Fig. 2e. Such heterophase interfaces are highly effective in inducing interfacial polarization, a key dielectric loss mechanism for EMA.

### Diversity and Tunability of the Properties

The remarkable diversity in composition and structure of TMDs family provides extensive tunability in their electronic and optical properties. The electrical characteristics of TMDs are predominantly governed by their phase structures. For instance, the 1T phase of MoS_2_ exhibits metallic behavior, whereas the 2H phase demonstrates semiconducting properties. Notably, 1T-MoS_2_ is thermodynamically metastable, while 2H-MoS_2_ is the stable phase, enabling precise property modulation through phase engineering [70].

**Fig. 3:**
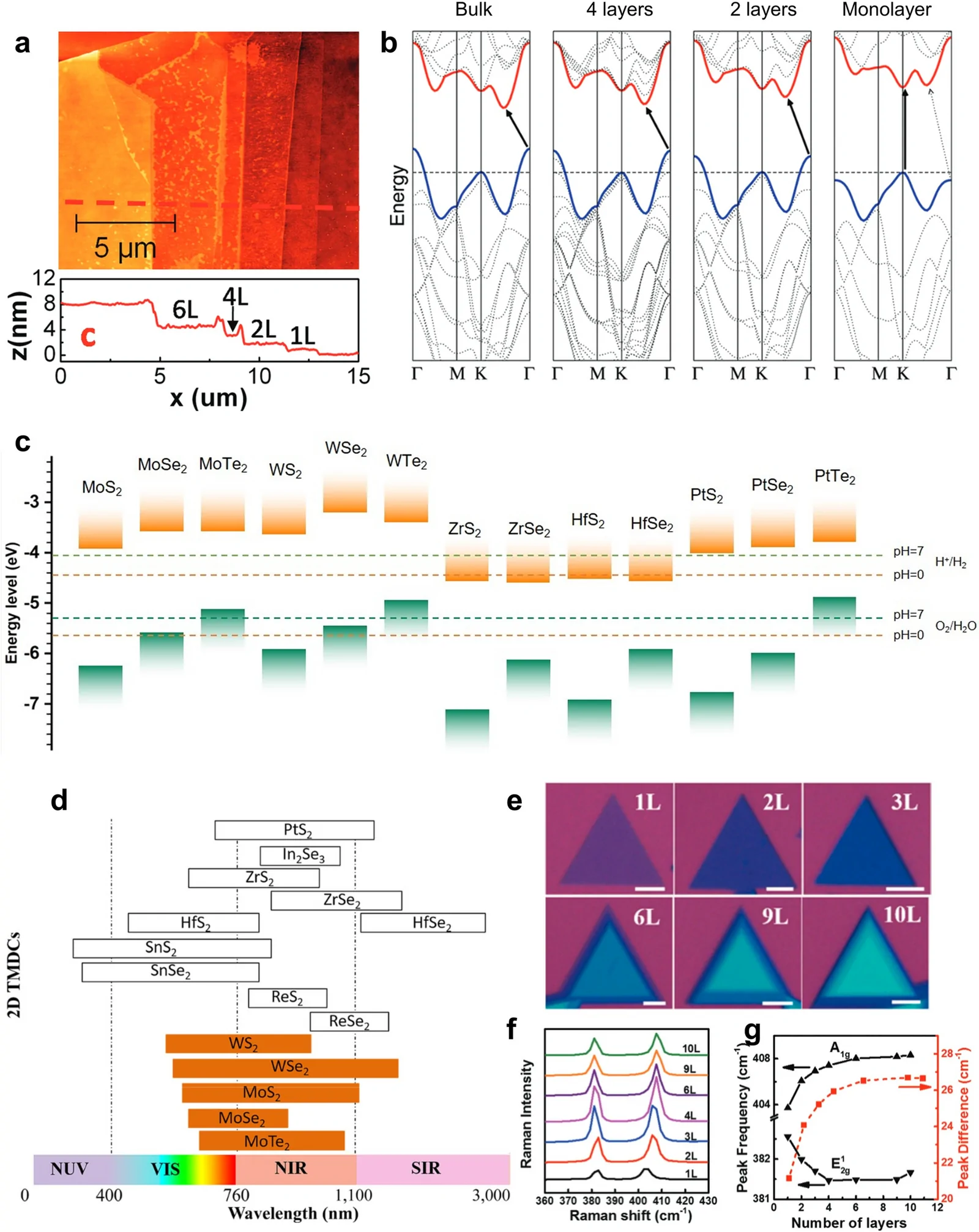
**a** Atomic force microscope (AFM) image of the exfoliated MoS_2_ sample; **b** calculated band structures of MoS_2_ with different layers [24]. Copyright 2010 American Chemical Society. **c** VBM and CBM positions of TMDs monolayers calculated by DFT method. The vacuum level is zero used as reference. Data collected from Ref. [73]. Copyright 2013 American Chemical Society. **d** Spectral responses of 2D TMDCs. NUV, VIS, NIR, and LIR represent near ultraviolet, visible, near infrared, and longwave infrared, respectively [75]. Copyright 2020 Springer Nature. **e** Optical images of MoS_2_ flakes with different layers on Si substrate; **f** Raman spectra of the MoS_2_ flakes with different layers. **g** Peak frequency variation of E_2g_^1^ and A_1g_ Raman mode and their difference [76]. Copyright 2017 Wiley-VCH

Furthermore, the electronic band structure of TMDs can be effectively tuned by varying the number of layers. When bulk MoS_2_ is exfoliated into monolayers, it undergoes a transition from an indirect to a direct bandgap semiconductor, with the bandgap ranging between 0.88 and 1.71 eV [24], as shown in Fig. 3a, b. This significant alteration in electronic behavior has positioned TMDs as promising candidates for next-generation electronic devices. The conduction band minimum (CBM) and valence band maximum (VBM) positions also vary across different TMDs compositions. As illustrated in Fig. 3c, first-principles calculations have been employed to map and compare the band edge positions of various monolayer TMDs compounds [73]. This bandgap tunability directly influences EMA performance. A narrower bandgap facilitates conductive loss, whereas layer‑controlled bandgap modulation can tailor dielectric polarization relaxation, both of which are critical for dielectric loss and impedance matching. The bandgap engineering of TMDs offers a similar pathway to optimize their EMA performance [74].

Additionally, distinct spectral responses are observed among different TMDs [75]. For example, typical TMDs like MoS_2_ exhibit strong absorption in the visible to near-infrared range, while HfSe_2_ demonstrates responsiveness in the far-infrared region (Fig. 3d). The optical and phonon vibrational properties of TMDs also exhibit pronounced layer-dependent characteristics. Figure 3e presents optical microscopy images of MoS_2_ flakes with varying layer counts on silicon substrates, where the apparent color systematically changes with thickness [76]. Correspondingly, Raman spectra (Fig. 3f, g) reveal that the A_1g_ mode undergoes a blueshift, while the E_2g_^1^ mode redshifts as the layer number increases from monolayer to few-layer configurations. These spectral shifts gradually converge toward bulk values beyond six layers. Moreover, numerous studies have reported layer-dependent variations in surface potential, work function, electrical conductivity, and carrier mobility, further highlighting the rich tunability of TMDs materials for tailored applications.

In summary, the diverse elemental compositions, tunable layered structures, and rich polymorphic phases of TMDs provide multiple degrees of freedom for tuning their dielectric properties. These structural features directly affect the electronic band structure, defect state density, and interfacial polarization behavior of TMDs, thereby determining their dielectric loss capability under EMW. Understanding these structure–property relationships is a prerequisite for designing high-performance TMDs-based EMA materials. Based on this, the following chapters will focus on structural regulation strategies, including defect engineering, phase transition, and heterointerface construction, to offer systematic guidance for optimizing the EMA performance of TMDs.

## Structure Regulation of TMDs-Based EMW Materials

Owing to their exceptional physicochemical properties outlined previously, TMDs have emerged as highly promising dielectric-type EMA materials over the past decade [77, 78]. The inherent layered structure, large specific surface area, favorable electrical conductivity, and abundant defect/edge sites enable even single-component TMDs to function as efficient microwave absorbers [79], exhibiting remarkable similarities to carbon-based materials in this regard. In contrast to carbon, however, TMDs offer additional advantages for EMA performance optimization, including polymorphic phase tunability and intrinsic nanosheet self‑assembly tendencies. As detailed in Sect. 3, TMDs possess highly tunable physicochemical characteristics. This section systematically examines various design and modulation strategies across multiple scales to optimize their EMA performance. These approaches span atomic-scale defect engineering and doping, nano-microscale phase engineering, and micro-meso-scale morphological control. By comprehensively discussing these multiscale modification methods for pure TMDs absorbers, we aim to establish fundamental design principles for developing next-generation TMDs-based absorption materials.

### Atomic Vacancy and Doping

Vacancy defect engineering has been demonstrated as an exceptionally effective approach for modulating the physicochemical properties of various EMW absorbers [80–82]. Guided by the "less is more" design philosophy, vacancies are regarded as sophisticated forms of crystal defects that can comprehensively enhance EMA performance through precise control of elemental composition, lattice configuration, and electronic distribution. Based on the charge state of the missing ions, vacancies can be categorized into anion-type and cation-type. Cation vacancies, due to the larger atomic scale and stronger chemical bonding of cations, typically require aggressive physical bombardment or chemical etching treatments for their formation [83]. These processes often simultaneously introduce anion vacancies, and large-scale defect such as cracks or pores [84]. In contrast, anion vacancies are more readily formed owing to the lighter mass and looser coordination structure of anions, making them widely prevalent in EMA applications. Extensive research has been conducted on O vacancies, establishing relatively universal principles, mature strategies, and critical insights into their fabrication, characterization, and application in EMA [85, 86]. In comparison, chalcogen (S/Se/Te) vacancy engineering, particularly in the realm of 2D TMDs, is currently experiencing its golden era of development. These materials exhibit tremendous potential and are expected to emerge as a dominant electromagnetic loss mechanism in next-generation EMA materials.

**Fig. 4:**
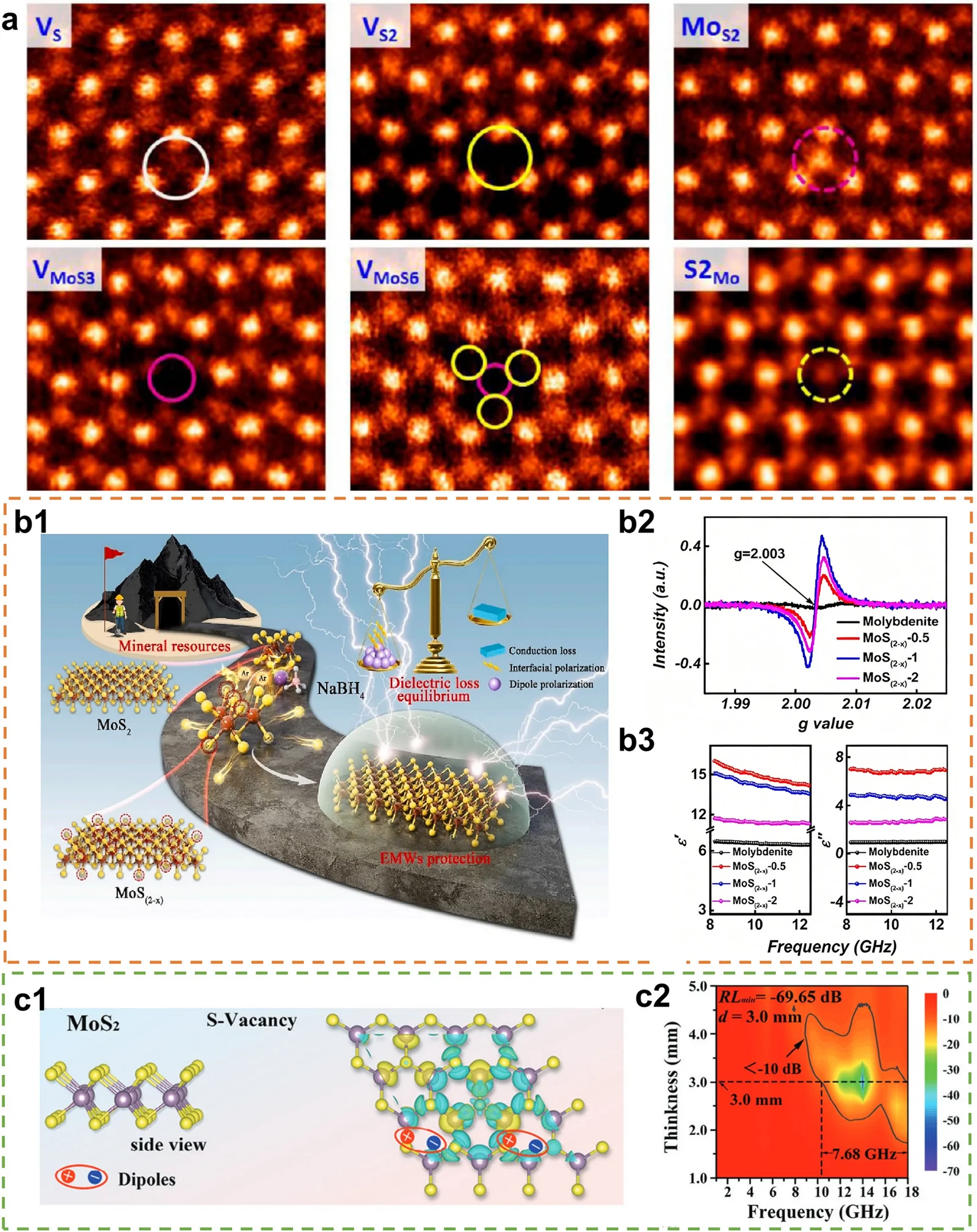
**a** Atomic resolution ADF-STEM images of different point defects present in monolayer MoS_2_, including V_S_,V_S2_,Mo_S2_,V_MoS3_,V_MoS6_, and S2_Mo_ [87]. Copyright 2015 Springer Nature. **b1** Natural molybdenite S vacancy engineering design ideas and main contents; **b2** EPR spectra of MoS_(2−*x*)_ samples (right top); **b3** permittivity curves of MoS_(2−*x*)_ samples (right bottom) [89]. Copyright 2023 Elsevier. **c1** Differential charge three-dimensional (3D) plots of S Vacancy MoS_2_; **c2** 2D RL plots of MoS_2_/V_2_O_3_ absorbers (right) [27]. Copyright 2024 Wiley-VCH

Monolayer MoS_2_ exhibits six predominant types of point defects, as illustrated in Fig. 4a. These include single sulfur vacancies (V_S_), double sulfur vacancies (V_S2_), composite vacancies formed by Mo atom with three surrounding S atoms (V_MoS3_), composite vacancies formed by Mo with three adjacent S dimers (V_MoS6_), as well as antisite defects involving Mo substituting for S dimers (Mo_S2_) and S dimers substituting for Mo (S2_Mo_) [87]. Hong et al. [88] systematically investigated these defects using first-principles calculations, determining their formation energy and enthalpy. The results reveal that V_S_ exhibit significantly lower formation energies compared to other defect types. Specifically, the formation energy of a V_S_ is merely 2.12 eV, substantially lower than that of a V_S2_ (4.14 eV) or a Mo vacancy (6.2 eV). These thermodynamic calculations demonstrate that sulfur vacancies are energetically favored in monolayer MoS_2_, with V_S_ being the most prevalent defect type, followed by V_S2_. In contrast, Mo vacancies and antisite defects are thermodynamically unfavorable and thus rarely observed under typical synthesis conditions. This defect hierarchy provides crucial insights for understanding and controlling the atomic-scale imperfections that influence the electronic and functional properties of MoS_2_.

Deng and colleagues developed an innovative approach to engineer S vacancies in MoS_2_ through NaBH_4_ reduction treatment of natural molybdenite (Fig. 4b1) [89]. This process successfully produced MoS_2−*x*_ materials with precisely controlled sulfur vacancy concentrations, as shown in Fig. 4b2. The introduction of sulfur vacancies significantly modified the electronic structure of molybdenite, leading to a reduced bandgap that facilitated EMA at lower energy levels. The sulfur vacancies enhanced the material's EMA capabilities through multiple mechanisms. First, they increased the probability of charge transfer within the molybdenite lattice, thereby improving conductive loss. Second, the vacancies created additional polarization-active sites that enhanced both interface polarization and defect polarization, resulting in substantially increased dielectric loss. Notably, polarization loss became the dominant mechanism in the high-frequency range, further boosting the overall EMA performance. Experimental results demonstrated that sulfur vacancy engineering effectively modulated the dielectric properties of MoS_2_ (Fig. 4b3). Among the synthesized samples, MoS_2−*x*_−1 exhibited optimal EMA characteristics, achieving a RLₘᵢₙ of −34.35 dB at a thickness of 2.1 mm. The EAB covered 4.04 GHz (13.96–18 GHz), showcasing the material's broadband absorption capability. These improvements were attributed to the synergistic effects of enhanced interface polarization, defect polarization, and conductive loss induced by the S vacancies. In MoS_2_-based EM absorbers, S vacancies play a crucial role in enhancing polarization loss by trapping additional charges and creating localized dipole moments. Wang et al. [27] provided compelling evidence for this mechanism through density functional theory (DFT) calculations, which revealed significant charge redistribution around sulfur vacancies. The differential charge density plots clearly illustrate the formation of strong electric dipoles, characterized by pronounced charge depletion regions (Fig. 4c1). When combined with other loss mechanisms inherent to the absorber system, including interfacial polarization and conductive loss, the presence of sulfur vacancies enables MoS_2_ absorbers to achieve exceptional broadband absorption performance. Specifically, the synergistic effects of these mechanisms result in an impressive EAB of 7.68 GHz, demonstrating the material's capability for wide-frequency EMW attenuation (Fig. 4c2). This work highlights the importance of defect engineering in optimizing the polarization characteristics of MoS_2_ absorbers, providing valuable insights for the design of high-performance, broadband EMW-absorbing materials.

In practice, two main routes exist for introducing S vacancies. Post‑synthetic reduction (e.g., NaBH_4_ treatment) can rapidly generate high vacancy concentrations but may cause uneven distribution and structural damage. Direct synthesis (e.g., tuning hydrothermal processes) offers more uniform vacancy distribution and better crystal integrity, though with a narrower tunable range and stricter control requirements. Each approach has merits and limitations, and the choice depends on specific research goals.

Due to their significantly lower formation energy, S vacancies are more likely to occur in various MoS_2_-based EMA material systems. Their superior EMW dissipation capability has been extensively confirmed in published studies [27, 40, 89, 90]. In contrast, the escape of Mo atoms from the lattice requires overcoming the constraint of S atomic layers, resulting in a higher formation energy. This process often leads to the formation of associated anion-cation complex vacancies (Fig. 4a). Although we have previously observed typical Mo vacancies at the edges of MoS_2_ nanosheets [91], no studies have yet reported the regulation of EMA performance via Mo vacancy engineering. The construction of S-vacancies can be achieved through either top-down strategies (including thermal annealing [92], ion irradiation [93], electrochemical etching [94], and liquid chemical etching [95]) or bottom-up approaches (such as solvothermal synthesis [96], target ablation [97] and doping-induced methods [98]), enabling precise control over vacancy concentration and distribution. In contrast, the formation of Mo vacancies typically requires more aggressive physical bombardment or chemical etching techniques [84]. Leveraging the well-established S vacancy construction methods, future research should focus on two key aspects: (1) precisely regulating the content and spatial distribution of S vacancies in MoS_2_-based EMA materials, (2) further fine-tuning vacancy clusters and configurations at atomic scales, and systematically elucidating and utilizing the enhanced EMA properties induced by S vacancies. This comprehensive approach represents a promising research direction for developing advanced MoS_2_-based absorbers with tailored performance characteristics.

**Fig. 5:**
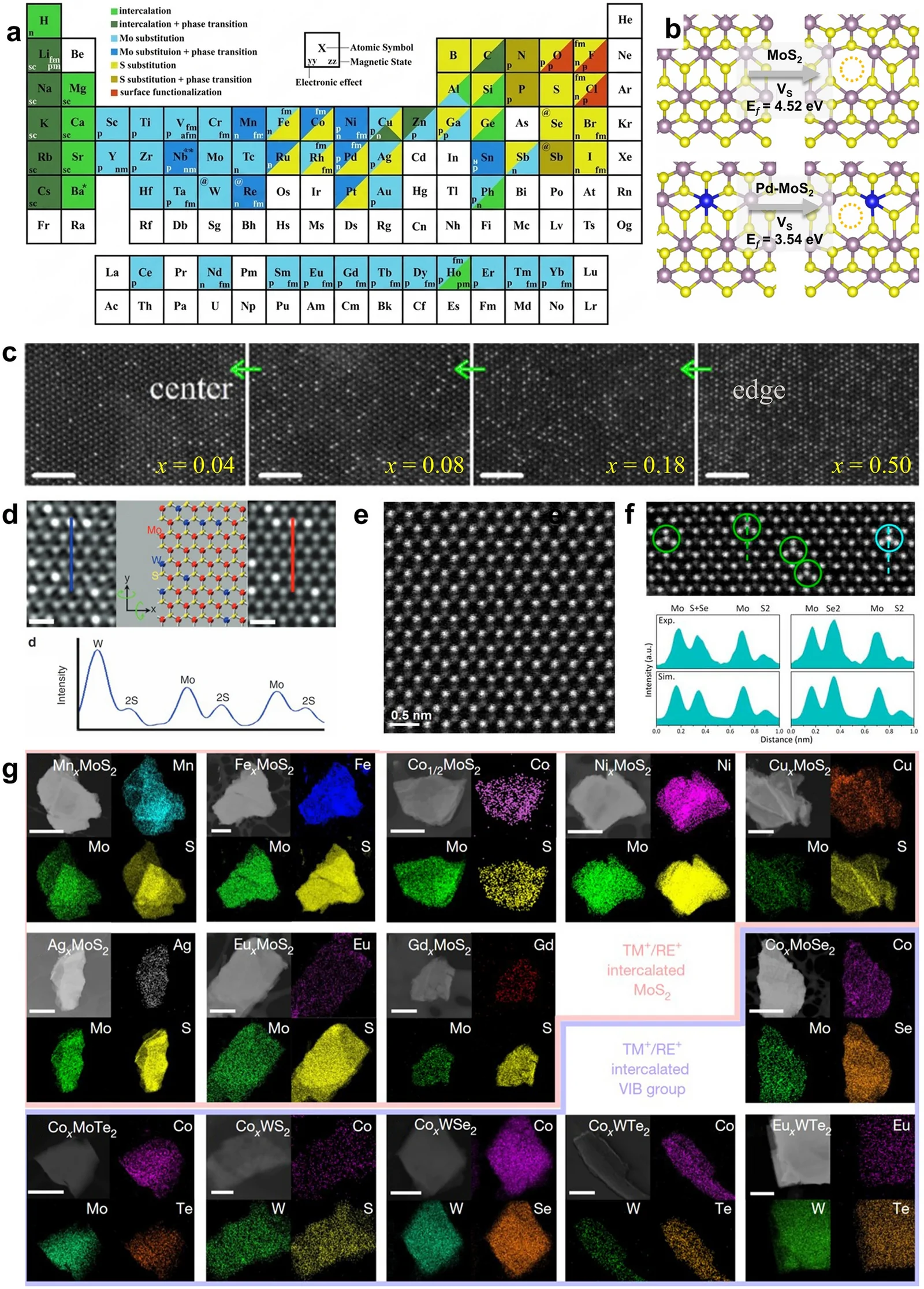
**a** Dopant positioning preferences across 2H-MoS_2_ frameworks driving coupled transformations in phase structure, electronic states, and magnetic configurations [102]. Copyright 2022 Elsevier. **b** Comparison of the relaxed atomic structures and formation energies of S vacancies in pristine MoS_2_ and Pd-doped MoS_2_ [98]. Copyright 2018 Springer Nature. **c** HAADF-STEM images of Mo_1−*x*_W_*x*_S_2_ alloy, from the Mo-rich configuration to W-rich configuration [115]. Copyright 2019 American Chemical Society. **d** Atomic-level Mo_1−*x*_W_*x*_S_2_ configuration and the corresponding simulated HAADF-STEM images (right) and measured HAADF-STEM images (left), and the intensity distribution along the marked path (bottom) [116]. Copyright 2013 Springer Nature. **e** Atomic-level HAADF-STEM image of MoS_2_ and **f** MoS_2(1−*x*)_Se_2*x*_ (top) and the corresponding intensity distribution along the marked path (bottom) [117]. Copyright 2014 American Chemical Society. **g** Cation exchange strategy for universal intercalation of TMDs [118]. Copyright 2025 Springer Nature

Atomic-scale doping demonstrates significantly lower formation energies compared to vacancy defects, with negative formation energies being readily achievable in sulfur-rich environments [99–101]. In the layered structure of MoS_2_, dopant atoms with varying electronegativities and atomic radii can form distinct configurations including intercalation, substitutional doping, and surface functionalization. Figure 5a systematically illustrates the preferred doping sites across the periodic table and the corresponding modifications in structural and electronic properties induced by different dopants [102]. Transition metals and rare-earth elements predominantly substitute molybdenum sites, inducing substantial alterations in both crystalline structure and electronic characteristics. Specifically, group IVB elements (Ti, Zr, Hf) doping in MoS_2_ shifts the Fermi level toward the valence band, creating p-type behavior [103, 104] that enhances catalytic activity while improving tribological performance and oxidation resistance [105]. Group VB dopants (V, Nb, Ta), featuring one fewer d-electron than Mo, generate new acceptor levels above the VBM [106]. This electronic restructuring results in Fermi level shifting, p-type conductivity, increased carrier concentration, improved in-plane conductivity [107], reduced bandgap, and facilitated interlayer electron transfer [108], collectively optimizing the material's functional performance. Ahmed et al. [109] found that the introduction of 5 at% vanadium doping into MoS_2_ nanosheets induces a room-temperature ferromagnetic phase with a remarkably high coercivity of 1.49 × 10^5^ A m^−1^. Theoretical studies reveal that substituting Mo with Re atoms introduces ferromagnetism, accompanied by spin polarization due to the additional valence electron from Re [110]. Moreover, the 1T phase stabilized by rhenium doping further enhances the magnetic properties of MoS_2_ nanosheets [111]. Mn doping, on the other hand, induces ferromagnetism through hybridization between S 3*p*, Mo 4*d*, and Mn 3*d* orbitals [112]. Both theoretical and experimental investigations confirm that Fe substitution for Mo leads to spin polarization and ferromagnetic ordering in MoS_2_ layers [113]. The strength and anisotropy of this ferromagnetism are strongly influenced by the strain introduced into the MoS_2_ lattice via doping. Notably, similar magnetic behavior has been observed in MoS_2_ doped with gadolinium (Gd) [101], holmium (Ho) [114], and dysprosium (Dy) [100], demonstrating the generality of this doping-induced magnetic modulation.

It is noteworthy that doping can facilitate the generation of vacancies, while the presence of vacancy defects, in turn, provides low-energy-barrier sites for dopant incorporation. As illustrated in Fig. 5b, researcher Luo et al. [98] employed DFT calculations to demonstrate that Pd doping creates favorable conditions for S vacancy formation in MoS_2_. Specifically, the formation energy of S vacancies decreases from 4.52 to 3.54 eV in Pd-doped MoS_2_. These two atomic-scale modulation strategies (doping and vacancy engineering) often coexist synergistically in MoS_2_ systems. This interplay further amplifies the defect density within the material and enhances the dipole moment, collectively contributing to a more pronounced EMA performance.

W-doped MoS_2_ represents the most extensively studied doping system of MoS_2_ in both theoretical and experimental contexts. Yang et al. [115] demonstrated that chemical vapor deposition (CVD) method enables the synthesis of monolayer Mo_1−*x*_W_*x*_S_2_ alloys with continuous compositional gradients across individual domains (Fig. 5c), achieving a seamless transition from MoS_2_ to WS_2_. This nearly arbitrary control over elemental composition offers broad opportunities for doping modulation, a strategy commonly referred to as alloying. To date, numerous experimental studies have reported the alloying of MoS_2_ with various elements, including systems such as MoS_2(1−*x*)_Se_2*x*_ [117], MoS_2(1−*x*)_Te_2*x*_ [119], Mo_1−*x*_W_*x*_S_2_ [120], Mo_1−*x*_Re_*x*_S_2_ [121], and Mo_1−*x*_Sn_*x*_S_2_ [122], among others.

The atomic-scale fine structure of such doping configurations requires characterization using spherical aberration-corrected HAADF-STEM. As shown in Fig. 5d, in W-doped MoS_2_, W atoms exhibit significantly brighter contrast compared to molybdenum atoms in HAADF-STEM images due to their higher atomic number. The simulated STEM phase results show excellent agreement with the experimental observations. A similar phenomenon occurs in anion-doped MoS_2_. Figure 5e, f presents STEM images of Se-doped MoS_2_, where selenium substitution at sulfur lattice sites produces brighter spots against the darker sulfur background, resulting in enhanced local intensity.

Alkali and alkaline earth metals exhibit a strong tendency to intercalate into the interlayers of MoS_2_ [123]. These ions donate electrons to the MoS_2_ layers, thereby enhancing the n-type doping effect and leading to improved electron donor characteristics [124]. This process often induces a phase transformation from the semiconducting 2H phase to the metallic 1T phase. The intercalation-induced expansion of the interlayer spacing facilitates the exfoliation of MoS_2_ in liquid media, a method that has been widely adopted. The earliest MoS_2_ nanosheets for EMA were indeed obtained through lithium intercalation followed by exfoliation from bulk crystals [20]. In contrast, transition metals and rare earth elements generally cannot be directly intercalated into the MoS_2_ lattice. Recently, Professor Huang Yu's team reported a cation exchange strategy that enables universal intercalation of transition and rare earth metals into various TMDs, as illustrated in Fig. 5g [118]. This breakthrough provides a novel and controllable pathway for modifying MoS_2_ beyond conventional atomic substitution doping.

**Fig. 6:**
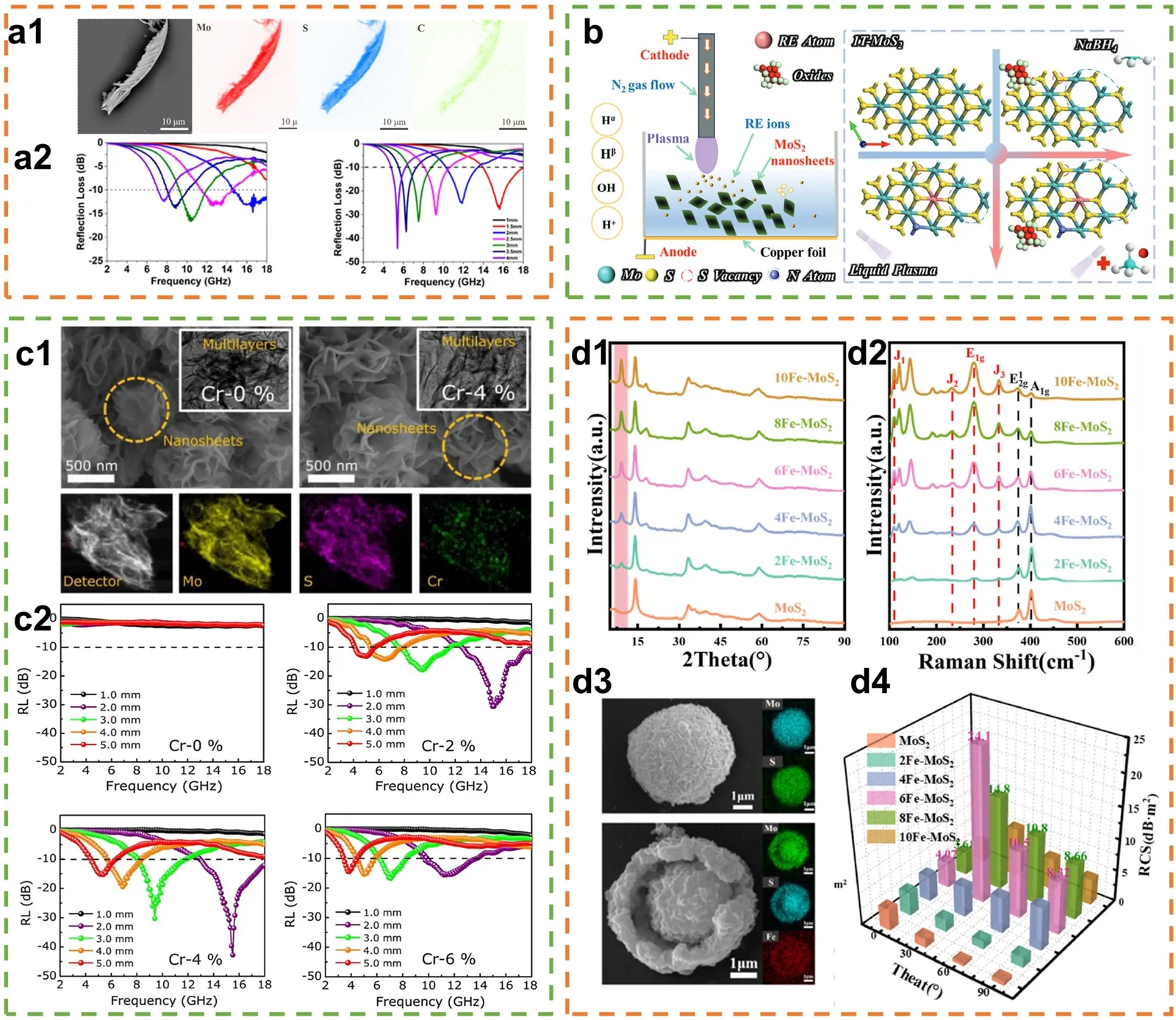
**a1** SEM image and corresponding EDS mappings of C-doped MoS_2_-1.5; **a2** RL curves of C-doped MoS_2_-0.5, 1.0, 1.5 respectively [125]. Copyright 2020 American Chemical Society. **b** Graphical summary illustrating the synthesis and system configuration for liquid plasma production, and the consequent phase transformation in the microstructure of IT-MoS_2_ post NaBH_4_ and liquid plasma treatment [34]. Copyright 2024 Wiley-VCH. **c1** SEM images of MoS_2_, Cr-4% doped MoS_2_ nanosheets and EDS mappings of Cr-4% doped MoS_2_; **c2** RL curves of Cr-0%, 2%, 4%, 6% doped MoS_2_ respectively [126]. Copyright 2018 Springer Nature. **d1** XRD patterns and **d2** Raman spectra of Fe doped MoS_2_ samples; **d3** SEM images and corresponding EDS mappings of MoS_2_ and 8Fe-MoS_2_ yolk-shell microspheres respectively; **d4** The RCS value reduction of the Fe doped MoS_2_ samples [127]. Copyright 2025 Elsevier

Doped TMDs has been extensively investigated for EMA [128]. Wang and colleagues developed a lightweight C-doped MoS_2_ aerogel with a unique bionic banana-leaf microstructure via self-assembly and directional freezing [125]. Using sodium alginate as both a template and carbon source, they constructed a precursor through assembly with ammonium thiomolybdate. Subsequent directional freezing and high-temperature pyrolysis (900 °C) yielded a porous C-doped MoS_2_ aerogel, as shown scanning electron microscope images (SEM) in Fig. 6a1. At a 30 wt% filler loading, the C-doped MoS_2_-1.5 aerogel achieved a RL_min_ of –43 dB at 5.4 GHz with a matching thickness of 4 mm. Moreover, it exhibited an EAB of 4 GHz (14–18 GHz) at a thickness of only 1.5 mm (Fig. 6a2). The EMA mechanism is primarily attributed to moderate conductive loss, interfacial polarization, multiple reflections, and excellent impedance matching. Professor Wu's team developed a novel liquid plasma-assisted strategy to incorporate rare earth elements (La, Ce, Pr) into the lattice of 1T-MoS_2_ (Fig. 6b), aiming to enhance its environmental stability [34]. Through a combination of experimental and theoretical approaches, the influence of hybridized RE-4*f* and Mo-4*d* orbitals on EMW attenuation was systematically investigated. The liquid plasma treatment significantly increased the 1T-phase content, reaching 82.69% in the Ce20-D7 sample. Introduction of rare earth elements promoted orbital hybridization between RE-4*f* and Mo-4*d*, facilitating the short-range migration of weakly bound electrons along the electric field direction and thereby enhancing polarization loss. The Pr15-D7 sample exhibited an EAB of 7.12 GHz at a thickness of 2.6 mm and a RL_min_ of –52.02 dB, while the Ce20-D7 sample achieved an EAB of 6.96 GHz at 2.7 mm. DFT calculations further confirmed that orbital hybridization enhances electronic interactions, leading to optimized EMA performance. Cr was also incorporated into MoS_2_ nanosheets via a hydrothermal method (Fig. 6c1) [126]. Both experimental and first-principles computational results demonstrate that Cr doping can induce ferromagnetism in MoS_2_. Although its influence on high-frequency permeability is limited, Cr doping significantly modifies the dielectric properties and improves the impedance matching of the nanosheets, rendering them promising for EMA applications. At an optimal doping concentration of 4.24 at%, the Cr-doped MoS_2_/paraffin composite (with 20 wt% filler loading) exhibits a RL_min_ of –43 dB and an EAB of 7.2 GHz (Fig. 6c2). Zhong et al. developed an innovative iron atomic doping strategy [127] to modulate the phase composition and defect characteristics of MoS_2_, leading to the successful synthesis of Fe-doped yolk-shell MoS_2_ nanoflower microspheres for EMA, as shown in Fig. 6d3. Experimental results indicate that the incorporation of Fe atoms not only creates atomic-scale defect sites acting as charge-enriched centers but also induces crystal lattice distortion, facilitating the formation of a high density of stable 1T/2H heterointerfaces (Fig. 6d1). The proportion of the metallic 1T phase increases with higher Fe doping concentrations (Fig. 6d2). The 8Fe-MoS_2_ sample exhibits exceptional EMA performance, achieving an EAB of 5.36 GHz (12.64–18 GHz) at a matching thickness of 1.95 mm and a RL_min_ of –72.18 dB at 1.65 mm as shown in Fig. 6d4. Multiscale simulations reveal that the outstanding absorption properties originate from the synergistic effects of Fe defect-induced polarization, interfacial polarization at the high-density heterointerfaces, electrical loss, and optimized EM propagation paths.

In summary, atomic-scale defects, such as vacancies and atomic doping, serve as effective approaches for modulating the properties of MoS_2_-based EMA materials. Substantial literature has confirmed the efficacy of such atomic-level engineering strategies [129]. However, compared to the well-established applications of doping and vacancy engineering in semiconductors, catalysis, and electrochemistry, research in the field of EMA remains at an early stage and is expected to enter a phase of significant growth. Promising research directions include: (1) synergistic doping with transition metals and nonmetals, and the coordinated control of doping and vacancies for tuning dielectric properties; (2) the enhancement of absorption performance through high-concentration doping (alloying), multi-element doping, and even high-entropy doping; and (3) intercalation-based high-concentration doping and its associated magnetic effects for improved magnetic loss.

### Phase Engineering Regulation

As described in Sect. 3.1, TMDs exhibit distinct polytypic characteristics, where variations in intralayer covalent bonding and interlayer stacking sequences define different crystalline phases and lead to significant differences in their physical properties. The notable contrast between metallic 1T-phase and semiconducting 2H-phase MoS_2_ provides substantial opportunities for phase engineering to tailor the dielectric and EMA properties of MoS_2_-based materials [29, 130]. In this section, we review common strategies for modulating phase composition and highlight specific applications and critical roles of phase-controlled MoS_2_ in EMA application.

Common strategies for phase engineering include ion intercalation, external irradiation, electric field control, mechanical strain, salt-assisted phase transformation, thermal treatment, and electron injection. (1) Ion Intercalation: Treatment with n-butyllithium solution induces the transition from the 2H to the 1T phase in MoS_2_. Studies show that lithium intercalation disrupts the original trigonal prismatic coordination, leading to rearrangement of molybdenum atoms into an octahedral 1T configuration. Electrochemical lithium intercalation has been reported to enable precise control of the intercalation amount by adjusting the cutoff voltage, mitigating the inhomogeneity issues associated with conventional chemical lithiation and enabling high-yield production of monolayer TMD nanosheets [131]. Additionally, intercalation of Na⁺ and K⁺ ions can also drive the 2H-to-1T transition [132, 133]. Zhang Hua et al. developed a proton intercalation/deintercalation method to achieve reversible phase transitions between 1T′-WS_2_ and 1T′d-WS_2_ [134]. By controlling proton concentration in a micro-battery setup, highly reversible phase switching was realized. (2) External Irradiation: Mild Ar plasma bombardment can locally induce the transition from 2H to 1T phase in monolayer MoS_2_ [135]. The resulting 1T/2H heterophase structures can be used to fabricate field-effect transistors with low contact resistance. Lin et al. [136] observed the real-time phase transition from 2H to 1T in monolayer MoS_2_ under electron beam irradiation in STEM, revealing an atomic glide mechanism involving an intermediate stripe phase prior to full transformation. Laser irradiation has also been employed to trigger the transition from 1T′-MoS_2_ to 2H-MoS_2_ [137], as confirmed by Raman and photoluminescence spectroscopy. (3) Electric Field Control: Wang et al. [138] demonstrated the first electrostatic gating-induced phase transition from 2H to 1T′ in MoTe_2_ using an ionic liquid gate. The transition was verified by Raman spectroscopy, accompanied by anisotropic scattering behavior. Zhang et al. [139] achieved reversible phase switching between the 2H and 2Hd phases in MoTe_2_ and Mo_1−*x*_W_*x*_Te_2_ within a solid-state resistive random-access memory device. Atomic-resolution STEM imaging clearly revealed structural changes before and after the phase transition. (4) Mechanical Strain: It has been confirmed that a tensile strain as small as 0.2% is sufficient to induce the 2H-to-1T′ phase transition in MoTe_2_ at room temperature [140]. The process is fully reversible, with the original phase restored upon strain release. (5) Salt-Assisted Phase Transformation: A universal salt-assisted strategy was developed for synthesizing high-purity 1T′-phase TMDs (e.g., WS_2_, WSe_2_, MoS_2_, MoSe_2_) [141]. By carefully selecting salt types and concentrations, precise control over phase purity was achieved. (6) Thermal Annealing: Thermal annealing induced a surface-specific phase transition from 1T to 2H in bulk 1T-TaS_2_ crystals [142]. Scanning tunneling microscopy showed that the transformation occurred only in the topmost layer, with the underlying structure retaining the 1T phase. (7) Electron Injection: Electron transfer driven by contact with [Ca_2_N]⁺·e⁻ resulted in the transition from 2H-MoTe₂ to 1T′-MoTe_2_ [143]. The low work function and high mobility of the electron-rich interlayer facilitated electron injection and subsequent phase change.

**Fig. 7:**
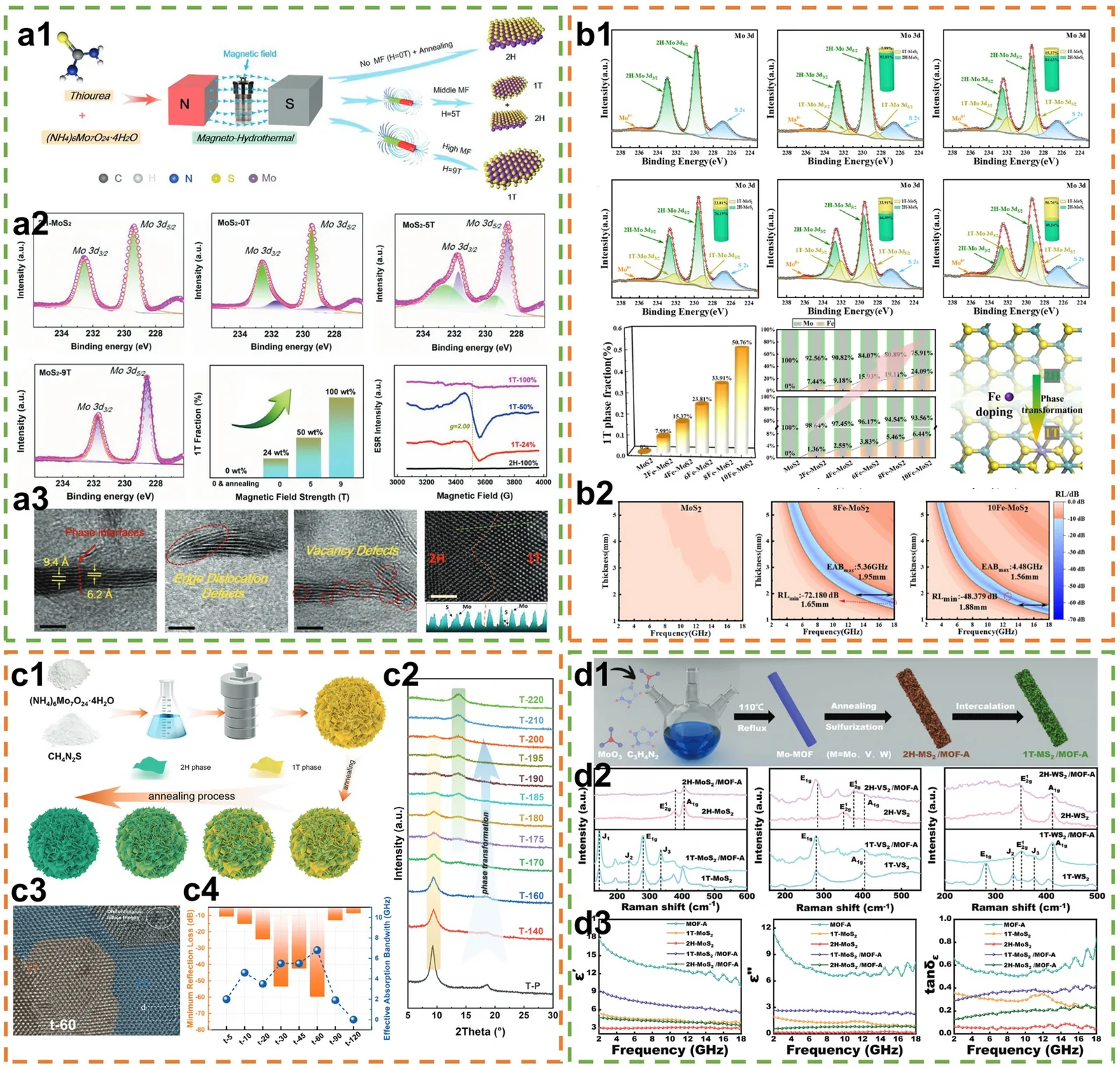
**a1** Graphical abstract illustrating the magneto-hydrothermal routes to tailor the phase composition of MoS_2_; **a2** High-resolution XPS spectra from the Mo 3d signal of 2H-MoS_2_, MoS_2_-0, 5, 9 T, and summary of 1T phase ratio and EPR spectra of the samples, respectively; **a3** HRTEM and STEM images of 1T-50% MoS_2_ [35]. Copyright 2021 Wiley-VCH. **b1** High-resolution XPS spectra of Mo 3d signal of Fe doped MoS2, summary of 1T phase ratio, the mole fraction of Fe element and Mo element in this materials from XPS spectroscopy and ICP-MS, and schematic diagram of Fe doping effect on the phase structure of MoS2; **b2** 2D RL plots of MoS2, 8Fe-MoS2 and 10Fe-MoS2, respectively [127]. Copyright 2025 Elsevier. **c1** Schematic illustration of the synthesis of 1T-MoS2 and phase transformation during the annealing process; **c2** XRD patterns of samples from T-P to T-220; **c3** HAADF-STEM images of t-60; **c4** comparison of EAB and RLmin of the samples, respectively [144]. Copyright 2024 Wiley-VCH. **d1** Schematic diagram of the preparation of 1T and 2H-MS2/MOF-A; **d2** Raman spectra of 1T-MS2,2H-MS2,1T and 2H-MS2/MOF-A (M = Mo, V, W); **d3** $\varepsilon^{\prime }$, $\varepsilon^{\prime \prime }$ and ${\mathrm{tan}}\delta_{e}$ curves of 1T/2H-MoS2/MOF-A samples, respectively [145]. Copyright 2024 Wiley-VCH

Research and applications across various fields have predominantly focused on 2H-MoS_2_ due to its high thermodynamic stability and tolerance toward diverse composite formation and functionalization [146]. However, the relatively low electrical conductivity of the 2H phase limits further enhancement of its dielectric loss and EMA performance. Through specialized synthesis and modification strategies, it is possible to fabricate MoS_2_ with coexisting 1T and 2H phases, leveraging the metallic 1T phase to improve conductive loss and significantly boost EMA properties [147]. In contrast to the diamagnetic behavior of the 2H phase, the octahedral coordination in 1T-MoS_2_ leads to asymmetric electron distribution and generates a net magnetic moment [148]. Capitalizing on this magnetic disparity, Ning et al. [35] employed a magnetic field-assisted hydrothermal method (Fig. 7a1) to precisely control the 1T phase content (0%, 24%, 50%, 100%) in MoS_2_ under external magnetic fields ranging from 0 to 9 T, achieving atomic-level regulation of phase boundaries (Fig. 7a2). Experimental results demonstrate that the sample with 50% 1T phase exhibits optimal EMA performance: a RL_min_ of –45.5 dB at 3.5 mm thickness and an EAB of 3.89 GHz. This enhancement is attributed to three synergistic mechanisms: strong charge transfer and interfacial polarization at 2H/1T heterointerfaces (Fig. 7a3), dipole polarization induced by sulfur vacancies, and appropriately optimized conductive loss. CST simulations further confirmed superior impedance matching and high power dissipation density in the 1T-50% sample. This study establishes for the first time a quantitative correlation between the phase composition of MoS_2_ and its electromagnetic attenuation performance, offering a new strategy for designing lightweight absorbers based on TMDs. Research demonstrates that in situ doping with trace amounts of Fe under optimized conditions serves as an effective strategy for modulating the phase composition of MoS_2_ [127]. Fe incorporation induces lattice distortion and generates atomic scale charge-enriched defect centers, while concurrently stabilizing 1T/2H heterointerfaces. With progressively increasing Fe doping concentrations, the 1T phase content rises from 0% to 50.76% in the 8Fe MoS_2_ sample (Fig. 7b1), establishing a dense and stable network of two-phase interfaces. This phase engineering approach significantly optimizes the electromagnetic parameters of the material through three complementary mechanisms: enhanced charge mobility via the 1T phase, maintained impedance matching by the 2H phase, and strong interfacial polarization at phase boundaries. The optimal 8Fe-MoS_2_ absorber achieves exceptional EMA performance, with a RL_min_ of −72.18 dB at 1.65 mm thickness and an EAB of 5.36 GHz at 1.95 mm (Fig. 7b2).

The intercalation of guest molecules or ions can facilitate the phase transition of MoS_2_ from the 2H to the 1T phase. Based on this principle, Professor Huang Ying's team developed a facile one-step hydrothermal method to prepare gram-scale 1T@2H-MoS_2_ with 61% 1T phase content by embedding guest species, and obtained pure 2H phase MoS_2_ via thermal treatment for comparison [149]. A systematic investigation into the influence of different phase structures on electromagnetic parameters revealed that the 1T/2H-MoS_2_ composite at 15% filler loading achieves a RL_min_ of –52.7 dB at 17.7 GHz with a thickness of 2.6 mm, and an EAB of 10.52 GHz, significantly outperforming the pure 2H phase. This enhancement originates from the improved dielectric loss due to the high electrical conductivity of the 1T phase, strengthened polarization effects (including interfacial and dipole polarization), and favorable impedance matching. Similarly, Zhang et al. employed a one-step hydrothermal method to incorporate highly conductive 1T phases into the 2H-MoS_2_ crystal matrix via NH_4_^+^ ion intercalation as a phase control strategy [150], thereby enhancing dielectric loss and forming heterostructures. Concurrently, CTAB was utilized as a soft template to construct hierarchical 3D microspheres, improving impedance matching. By systematically modulating both the 1T phase content and the morphological architecture, the electromagnetic parameters and EMA performance of MoS_2_ were thoroughly investigated. The optimized sample, denoted as A-MoS_2_-2, achieved a RL_min_ of –53 dB at a thickness of 1.99 mm and an EAB of 5.6 GHz at 1.77 mm, fully covering the entire Ku band. Liu et al. [151] investigated the influence of the sulfur-to-molybdenum (S/Mo) ratio under hydrothermal conditions on the 1T phase content and its corresponding effect on EMA performance. Their study revealed that at an S/Mo ratio of 4.5, the MoS_2_-4.5 sample exhibited the highest 1T phase content, reaching 66 wt%. This unique metal–semiconductor heterostructure significantly enhanced the electrical conductivity and polarization loss capability of the material. X-ray diffraction (XRD), Raman, and X-ray photoelectron spectroscopy (XPS) characterizations confirmed that the formation of the 1T phase originated from NH_4_^+^ ion intercalation, while transmission electron microscope (TEM) analysis revealed alternating 1T/2H nanodomains and abundant lattice defects within the nanosheets. Electromagnetic parameter measurements indicated that the increased 1T phase content moderately elevated the imaginary part of the complex permittivity (*ε*″) and optimized impedance matching. As a result, the composite with 50 wt% paraffin loading demonstrated excellent EMA performance, achieving a RL_min_ of –27 dB at 7.12 GHz with a thickness of 4.22 mm, and an EAB of 4.16 GHz covering 11.84–16 GHz. To enable more convenient and precise control of the 1T/2H phase ratio while minimizing alterations to the morphological characteristics and self-assembly behavior of MoS_2_, Huang et al. developed a streamlined hydrothermal synthesis method for preparing 1T phase enriched MoS_2_ with a 1T content reaching 76% (Fig. 7c1) [144]. Subsequent controlled annealing treatments enabled fine monotonic adjustment of the phase ratio, establishing a strong correlation between phase composition, complex permittivity, and electrical conductivity (Fig. 7c2). Precise phase ratio regulation substantially enhanced the EMA capabilities, yielding a RL_min_ of 59.8 dB at 2.68 mm and an EAB of 6.8 GHz at 2.48 mm (Fig. 7c4). This transition from high energy to low energy states proves highly controllable and straightforward compared to the reverse process, thereby facilitating mechanistic studies of phase engineering mediated dielectric property modulation.

Furthermore, Jia et al. [145] successfully achieved precise control over the interlayer spacing of TMDs on metal–organic framework (MOF) through an intercalation-annealing strategy, thereby inducing a phase transition between the 1T and 2H phases (Fig. 7d1). The 1T phase, characterized by its larger interlayer spacing, higher sulfur vacancy concentration, and metallic conductivity, exhibits superior synergistic effects of interfacial polarization and conduction loss. Comprehensive characterization techniques including XRD, TEM, Raman spectroscopy, and XPS confirmed distinct crystal structures between 1T-MoS_2_ (with an interlayer spacing of 0.98 nm) and 2H-MoS_2_ (0.67 nm), revealing that the 1T phase facilitates faster charge transfer and offers more active sites (Fig. 7d2). EMA measurements demonstrated outstanding performance of the 1T-MS_2_/MOF-A composite at a 45 wt% filler loading: 1T-MoS_2_/MOF-A achieved a RL_min_ of –61.07 dB at 3.0 mm and an EAB of 7.2 GHz at 2.3 mm; 1T-VS_2_/MOF-A and 1T-WS_2_/MOF-A attained EAB values of 5.2 and 5.84 GHz, respectively. Debye relaxation analysis and *α* calculations indicated that the dominant loss mechanisms originate from heterointerface polarization between the 1T phase and the carbon-based MOF-A matrix, coupled with high conduction loss attributable to the metallic phase. Impedance matching analysis further confirmed that the 1T phase effectively balances the dielectric parameters (Fig. 7d3), allowing greater EMA penetration and subsequent dissipation. By systematically comparing three TMDs materials (MoS_2_, VS_2_, WS_2_), this study mitigates interference from intrinsic material properties and identifies the critical role of metal–semiconductor interfacial polarization in EMA, thereby providing both theoretical insights and a novel phase-engineering strategy for designing high-performance microwave absorbers.

It should be noted that defect/doping engineering and phase engineering play distinct yet complementary roles in modulating the carrier behavior and dielectric response. Defect engineering mainly introduces local defect states and carrier traps, enhancing dipole polarization and hopping conductivity with a relatively mild effect on the real part of permittivity. In contrast, phase engineering (e.g., 2H to 1T transition) significantly alters the long range conductivity by modifying the electronic band structure of TMDs. However, phase engineering is not always aimed at enhancing conductivity; when excessive conductivity causes impedance mismatch, reducing the content of the metallic 1T phase can appropriately lower the conductivity and restore the penetration of EMW into the material. Therefore, in practical design, the 1T/2H ratio should be flexibly tuned to achieve the best trade-off between conductive loss and impedance matching according to the specific absorption requirements.

As discussed above, phase engineering plays a decisive role in modulating the dielectric properties of MoS_2_. Heterophase 1T/2H MoS_2_, which balances impedance matching and loss capability, exhibits excellent EMA performance. Current research challenges are primarily concentrated in two aspects: (1) developing convenient and efficient methods to adjust the phase ratio in various TMDs and establishing a systematic mechanism by which phase engineering regulates absorption properties; (2) integrating phase-engineered TMDs with other functional components in composite absorber systems to achieve multifunctional and intelligent EMA materials.

### Micro/Meso-scale Morphology Control

**Fig. 8:**
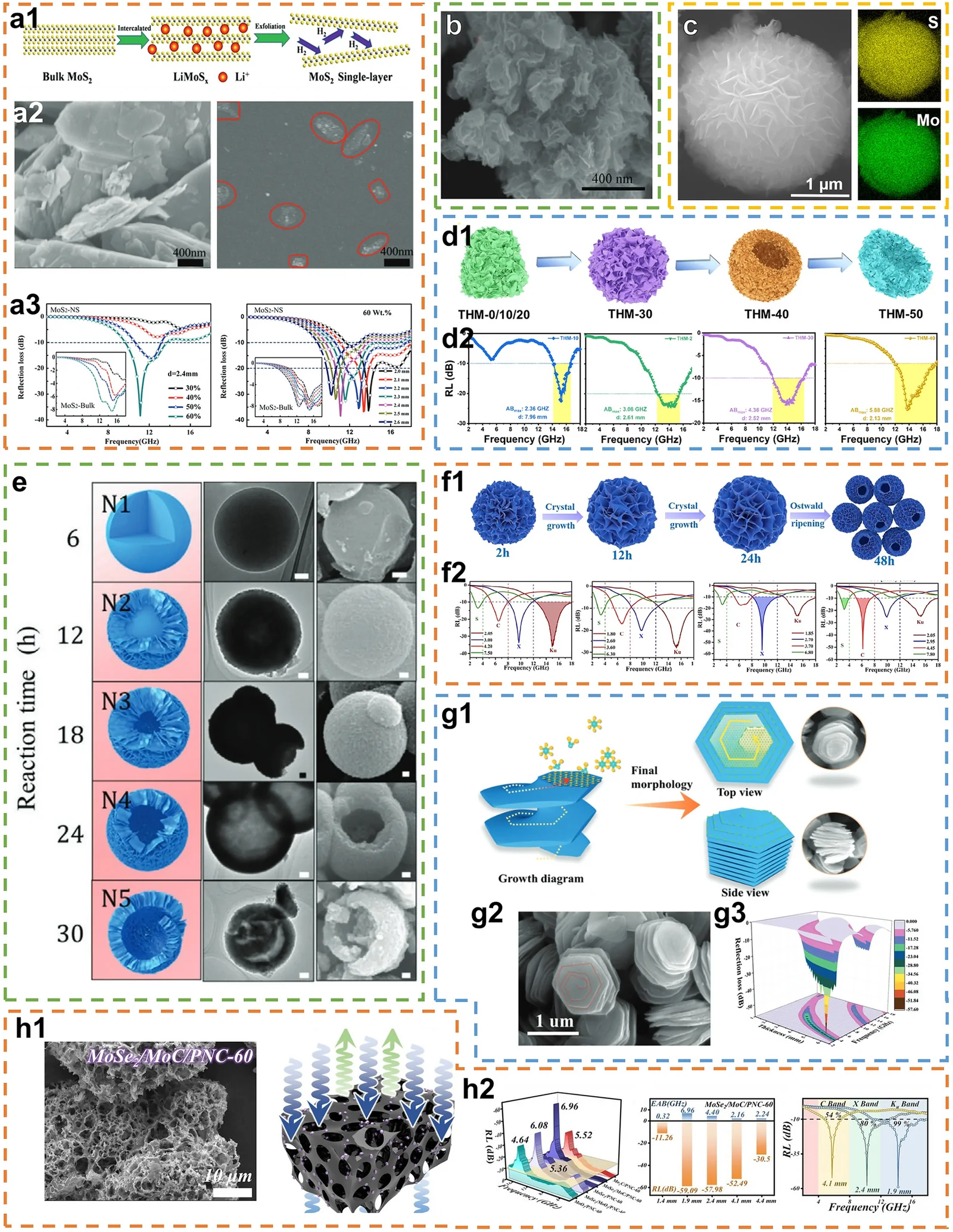
**a1** Schematic diagram of Li intercalation method for exfoliation of MoS_2_ nanosheets; **a2** SEM images of MoS_2_ bulk and nanosheets, respectively; **a3** RL curves of MoS_2_-NS with different filling ratio at 2.4 mm and at different thickness with 60 wt%, respectively [20]. Copyright 2015 Royal Society of Chemistry. **b** SEM image of typical MoS2 nanosheets synthesized by hydrothermal method [33]. Copyright 2016 Royal Society of Chemistry. **c** Typical self-assembled micro-flowers SEM image of MoS2 nanosheets and corresponding EDS mappings [144]. Copyright 2024 Wiley-VCH. **d1** Schematic illustration for evolution processes of MoS2 micro-morphology by adjusting the solvent contents; **d2** RL curves of MoS2 with different morphology [155]. Copyright 2023 Elsevier. **e** Schematic of the regulation of the morphology of hollow-sphere NbS2 [30]. Copyright 2021 Wiley-VCH. **f1** Schematic of evolution process of flower-like MoSe2 microspheres morphology influenced by solvothermal duration; **f2** RL curves of MoSe2 with different morphology [33, 36]. Copyright 2019 Elsevier. **g1** Schematic illustration of the growth of hexagonal VS2 nanosheets; **g2** SEM image of coaxial stacking VS2 nanosheets; **g3** 3D RL plots of sample S-190 [156]. Copyright 2020 Royal Society of Chemistry. **h1** MoSe2/MoC/PNC-x porous morphology and schematic illustration of interaction with EMW; **h2** EMA performance of MoSe2/MoC/PNC-60 [157]. Copyright 2023 Springer Nature

Compared with the two aforementioned modulation strategies, morphological control operates at a larger scale, ranging from hundreds of nanometers to hundreds of micrometers. The topological morphology significantly influences the transmission path and multiple reflection-scattering behavior of EMW, and governs the formation of conductive networks [152], thereby exerting a substantial effect on both the dielectric properties and EMA performance. Although naturally occurring MoS_2_ exists typically in bulk form, TMDs intrinsically tend to form 2D layered structures, which can be readily exfoliated or synthesized into MoS_2_ nanosheets. Cao et al. first reported the dielectric constant and EMA properties of MoS_2_ nanosheets obtained via n-butyllithium intercalation and exfoliation (Fig. 8a1) [20]. SEM images revealed that the exfoliated MoS_2_ nanosheets were smaller and more uniform compared to bulk MoS_2_ (Fig. 8a2). TEM observations confirmed that the nanosheets were highly flexible and ultrathin, with a thickness of approximately 2.6 nm. The MoS_2_ nanosheet/paraffin composite with 60 wt% filler loading exhibited a RL_min_ of –38.42 dB at a thickness of 2.4 mm and an EAB of 4.1 GHz, covering the frequency range of 9.6–13.76 GHz (Fig. 8a3). In contrast, the bulk MoS_2_/paraffin composite only achieved an RL_min_ of –8.24 dB. These results indicate that MoS_2_ nanosheets offer remarkable advantages in EMA applications, attributable to their higher specific surface area and abundant surface defects such as sulfur and molybdenum vacancies. These defects act as electric dipoles under an alternating electric field, leading to enhanced dielectric loss and thereby improved EMA performance.

MoS_2_ nanosheets synthesized via hydrothermal methods typically exhibit substantial wrinkling, deviating from idealized flat 2D plates [153, 154]. This crumpled and curved morphology is generally beneficial for EMA [33], as it exposes more edges and defect sites, creates greater porosity and specific surface area, prolongs EMW propagation paths, and facilitates the formation of interconnected conductive networks, as shown in Fig. 8b. By carefully modulating the hydrothermal parameters, these nanosheets can be guided to self-assemble into flower-like architectures (Fig. 8c) [144]. These 3D flower-like microspheres further enhance the EMA performance of the material. The interlocking of nanosheets and the resulting porous structure not only reduce the overall material density, favoring lightweight design, but also significantly extend the EMW travel path through multiple reflections and scattering mechanisms, thereby enhancing electromagnetic energy dissipation. Moreover, the hierarchical porous architecture provides abundant heterogeneous interfaces, inducing strong interfacial polarization and simultaneously promoting impedance matching. It is noteworthy that such an open 3D conductive network maintains high electrical conductivity while avoiding impedance mismatch, enabling the material to exhibit both strong absorption and broad effective bandwidth across a wide frequency range. Consequently, the hydrothermal method offers a simple yet effective route for synergistic control of material morphology from the microscopic to mesoscopic scales, providing a scalable strategy for designing high-performance microwave absorbers.

Liang and colleagues successfully synthesized a 1T/2H-MoS_2_ composite with a 3D flower-like hollow structure by modulating the solvent ratio of deionized water to anhydrous ethanol (Fig. 8d1) [155]. They systematically investigated the influence of microstructural morphology on EMA performance. The study revealed that when the volume of deionized water exceeded 30 mL, the material formed a more regular flower-like hollow architecture, as exemplified by the THM-40 sample. This unique morphology offers three major advantages. First, the hollow structure formed by layered stacking provides abundant interfaces, enhancing multiple reflections and scattering of EMW. Second, the incorporation of the metallic 1T phase significantly improves electrical conductivity, thereby promoting conductive loss. Third, dipole polarization and interfacial polarization, induced by sulfur and molybdenum vacancies, synergistically enhance dielectric loss. By precisely controlling the solvent ratio (40 mL water and 10 mL ethanol), the optimized THM-40 sample exhibited exceptional EMA performance. It achieved a RL_min_ of –56.32 dB at 17.16 GHz with a thickness of 1.85 mm, and an EAB of 5.88 GHz (ranging from 11.96 to 17.84 GHz) at 2.13 mm, covering 98% of the Ku band (Fig. 8d2). The study confirms that this morphology control strategy optimizes both impedance matching and attenuation capacity, achieving a synergistic improvement in thin thickness, strong absorption, and broad bandwidth.

This morphology control strategy is equally applicable to other TMDs. Cao and colleagues [30] systematically investigated the solvothermal synthesis of NbS_2_, a Group VB TMDs, focusing on microstructural modulation and its effects on EMA. Their study demonstrated that precise control of reaction parameters, including time, temperature, and precursor ratio, enables fine-tuning of NbS_2_ nanostructures, particularly the wall thickness (ranging from 126 to 577 nm) of hollow spheres and the stacking mode of nanosheets, as shown in Fig. 8e. Under optimal conditions (210 °C, 24 h, Nb:S = 1:11.7), the obtained NbS_2_ hollow nanospheres, composed of vertically aligned nanosheets, exhibited exceptional EMA properties. The material possessed a unique 1T/2H polytypic structure. When the phase ratio approached 1:1, a RL_min_ of –43.35 dB was achieved at 7.82 GHz. By reducing the precursor amount to decrease the wall thickness to 126 nm, an EAB of 6.48 GHz was attained in the 11.52–18 GHz frequency range, with an RL_min_ of –43.85 dB. The performance enhancement is attributed to three mechanisms: interfacial polarization enhanced by abundant active sites at edges and basal planes; multiple internal reflections enabled by the hollow spherical architecture; and facilitated electron transition within conductive networks formed by stacked nanosheets. Furthermore, the hollow porous structure improved impedance matching, while surface defects and functional groups increased relaxation loss.

In a similar manner, Ji et al. precisely engineered the microstructure of MoSe_2_ via solvothermal synthesis (Fig. 8f1) [36], constructing a series of hierarchical flower-like microspheres with tunable density, ranging from solid to hollow (denoted S2 to S48). With prolonged reaction time (from 2 to 48 h), the morphology evolved from compact petals (S2) to open petals (S24) and finally to hollow structures (S48), significantly increasing the specific surface area (from 16.7 m^2^ g^−1^ for S2 to 18.8 m^2^ g^−1^ for S48) and porosity. This morphological evolution directly influenced the electromagnetic properties: the dense structure (S2) exhibited strong performance in the Ku-band (12–18 GHz) with a RL_min_ of –36 dB and a 5.7 GHz bandwidth, owing to its high permittivity. The open structure (S24) achieved full coverage of the X-band (8–12 GHz) with a RL_min_ of –57.2 dB. Meanwhile, the hollow architecture (S48) effectively reduced the equivalent permittivity, extending the absorption into the lower-frequency C-band (4–8 GHz) with a bandwidth of 2.4 GHz and a RL_min_ of –51.6 dB, as shown in Fig. 8f2. The study elucidated the structure–performance correlation: the hierarchical morphology prolongs the microwave propagation path through multiple scattering, the 2D layered structure enhances conductive loss, and pore engineering optimizes impedance matching. Cao and colleagues precisely tuned the morphology and 1T/2H phase ratio of coaxially stacked VS_2_ nanosheets by controlling the hydrothermal temperature from 170 to 200 °C (Fig. 8g1) [156]. The sample synthesized at 190 °C exhibited efficient dual‑band absorption in the C‑band (4–8 GHz) and Ku‑band (12–18 GHz). The low‑frequency (C‑band) absorption, which is rare for non‑magnetic dielectric materials, originates from the unique coaxial stacking architecture that prolongs EMW propagation paths and enhances multiple reflections, combined with moderate conductivity that optimizes impedance matching at longer wavelengths. The material achieved a RL_min_ of –57 dB at 4 GHz and an EAB of 7 GHz (Fig. 8g3). Morphological analysis showed that increasing temperature transformed the nanosheets from rough irregular shapes into well‑stacked hexagonal configurations at 190 °C, while excessive temperature (200 °C) disrupted the stacking order. The synergistic modulation of conductivity and dielectric loss via defect engineering and phase composition balanced impedance matching and attenuation. This work demonstrates that morphology‑structure‑property control in VS_2_ nanosheets can overcome the low‑frequency absorption limitations of non‑magnetic materials, offering a strategy for multi‑band EMA design.

Generally, constructing stable 3D foam or aerogel structures from MoS_2_ presents greater challenges compared to graphene [158]. This difficulty primarily arises from several key factors. First, the relatively weak van der Waals forces between MoS_2_ layers often lead to structural collapse during the assembly of 3D architectures. In contrast, graphene benefits from stronger covalent bonding between *sp*^2^ hybridized carbon atoms, which enhances its structural integrity [159]. Second, the formation of robust foams or aerogels typically requires effective cross-linking and supporting frameworks. Graphene, with its superior electrical conductivity and tunable surface properties, can more readily form stable 3D networks through chemical or physical methods [160]. Conversely, the surface chemistry of MoS_2_ is less amenable to modification, which limits its ability to remain stably dispersed in solution and to assemble into tailored macrostructures. Graphene can be functionalized through oxidation, such as in graphene oxide, to introduce diverse functional groups that improve dispersibility and interfacial interactions with other materials. In an innovative approach, Worsley et al. [161] reported a detailed method for fabricating ultra-low-density monolithic WS_2_ and MoS_2_ aerogels via freeze-drying and thermal decomposition of precursors. The research team employed ammonium thiomolybdate and ammonium thiotungstate as precursors. A 3D network was first formed through freeze-drying, followed by thermal decomposition at 450 °C under a hydrogen atmosphere. This process successfully yielded MoS_2_ and WS_2_ aerogels with densities as low as 0.4%–0.5% of their theoretical values. SEM and TEM characterization revealed that the MoS_2_ aerogel exhibits a randomly oriented sheet-like morphology with varying degrees of curvature, whereas the WS_2_ aerogel consists of locally aligned smooth nanosheets. Both materials are composed of nanoparticles approximately 5–10 nm in size and 2–6 layers thick, exhibiting well-crystallized intralayer structures but overall turbostratic stacking. The Young’s modulus of both MoS_2_ and WS_2_ nanosheets with chitosan and glutaraldehyde, followed by liquid-phase freeze-drying, they produced an MoS_2_ aerogel exhibiting high porosity, with pore sizes ranging from 20 to 50 µm, and excellent mechanical stability, sustaining compressive elastic strains of up to 60%.

In the research field of EMA materials, 3D aerogel architectures based on TMDs are typically fabricated as multicomponent systems incorporating carbon materials or other functional constituents [162–165]. To the best of our knowledge, there have been no publicly reported instances of pure TMDs-based aerogels being utilized for EMA applications. For instance, Wu et al. designed a 3D porous EMA material via a salt-melt synthesis strategy, focusing specifically on the influence of TMDs morphology regulation on EMA performance [157]. The research team utilized PVP with varying molecular weights to modulate the hierarchical pore structure of a porous nitrogen-doped carbon framework, thereby optimizing the impedance matching characteristics of the material. By introducing a MoC transition layer through interfacial engineering, a MoSe_2_/MoC/PNC heterostructure was constructed (Fig. 8h1), which significantly enhanced the electrical conductivity and interfacial polarization effects, leading to improved dielectric loss capabilities. Experimental results indicated that the composite exhibits superior broadband absorption performance across the C, X, and Ku bands, achieving a RL_min_ of −59.09 dB at a thickness of 1.9 mm and an EAB of 6.96 GHz (Fig. 8h2). Furthermore, the 3D porous structure also imparts excellent marine anti-corrosion properties to epoxy resin coatings by prolonging the diffusion path of corrosive agents. This work offers new insights into the development of tunable multifunctional EMW absorbers with broadband performance from the perspective of TMDs morphology control and porous structural design.

In this chapter, we review recent advances in the multiscale design and modification of pure TMDs-based EMA materials, leveraging the highly tunable structural properties inherent to their extensive material family. We systematically summarize the crucial roles of atomic‑scale doping and vacancy engineering, micro‑scale phase control, and meso‑scale morphology strategies in enhancing EMA performance. Among these strategies, each has distinct advantages and limitations. Vacancy and doping engineering at the atomic scale can introduce local defect states to enhance dipole polarization and allow tunable permittivity, yet the controllable range of defect concentration and distribution remains limited due to current synthetic methods such as hydrothermal processes. Phase engineering, which involves the transition between semiconducting 2H and metallic 1T phases, significantly boosts conductivity and provides additional phase interface polarization. However, excessive metallic phase content tends to cause impedance mismatch, and the 1T phase is thermodynamically metastable, raising concerns about long‑term stability. Morphology regulation at the meso‑scale, including flower‑like, hollow structures, prolongs EMW propagation paths, improves impedance matching, and offers lightweight characteristics. Nevertheless, the reproducibility of such complex morphologies is often poor due to high sensitivity to synthesis conditions. Benefiting from their 2D layered architecture, intrinsic self‑assembly capability, tunable electrical conductivity due to polytypic phase coexistence, and enhanced dipole polarization from defects and dopants, pure TMDs have demonstrated promising EMA properties. Optimal performance usually requires cross‑scale synergistic design to balance loss capability and impedance matching. Looking forward, major research directions for pure TMDs‑based absorbers include: (1) multi‑element synergetic doping and even high‑entropy doping to increase polarization loss and improve impedance matching, thereby broadening the EAB; (2) high‑concentration substitution, alloying, or intercalation to induce dielectric enhancement and possible magnetic effects; (3) innovative morphological control and rational three‑dimensional aerogel design to reduce absorber density and introduce additional functionalities such as thermal management.

## Multicomponent Synergy for TMDs-Based EMW Materials

To further enhance EMA performance, combining TMDs with other components to form composite materials represents an effective and straightforward strategy. TMDs maintain their characteristic lamellar nanostructure during composite synthesis, preferentially serving as wrinkled nanosheet building blocks that vertically align with secondary components. Crucially, the nanosheet edges exhibit defect-rich terminations abundant in active sites. These reactive edges readily integrate with dissimilar materials through interfacial bonding, establishing robust heterointerfaces that provide TMDs with exceptional compatibility for multicomponent hybridization. This intrinsic structural feature facilitates the formation of chemically coupled architectures essential for functional composites. The construction of composites introduces abundant heterogeneous interfaces, which significantly enhance interfacial polarization loss. Additionally, the compositing process introduces more crystalline defects, thereby inducing additional defect dipole polarization. For semiconducting 2H-MoS_2_, the incorporation of highly conductive components can effectively improve EMW dissipation. Furthermore, composite constituents with specific properties can impart unprecedented multifunctional integration capabilities to TMDs-based absorbers. In this section, we provide a detailed review of recent advances in TMDs-based composite EMA materials, summarize current development trends, and offer insights to guide the design of next-generation TMD-based absorbers.

### C/TMDs Composites

Carbon materials constitute the earliest investigated and most significant EMW absorbers [166, 167]. Their multifaceted merits including low density, superior electrical conductivity, tailorable defect engineering, facile synthesis protocols, and exceptional chemical stability establish carbon architectures as preeminent candidates for absorption applications [168]. Hybrids integrating TMDs with carbon matrices leverage their shared 2D configurations and highly tunable properties to critically modulate permittivity spectra, reconcile impedance mismatch constraints, and achieve efficient electromagnetic energy dissipation. Given the pivotal role of carbon matrices in advancing EMA technologies, evidenced by extensive research on their tunable dielectric properties and unique structural hierarchies, this review deliberately dedicates a stand-alone section to systematically analyze these materials distinct from conventional dielectric substances. Based on the morphological dimensionality profiles of obtained composites, this review systematically categorizes pioneering developments across three structural classifications: one-dimensional (1D) fiber-like assemblies, 2D stacked heterolayers, and 3D hierarchical architectures comprising carbon and TMDs.

#### 1D Fiber-Like C/TMDs Composites

**Fig. 9:**
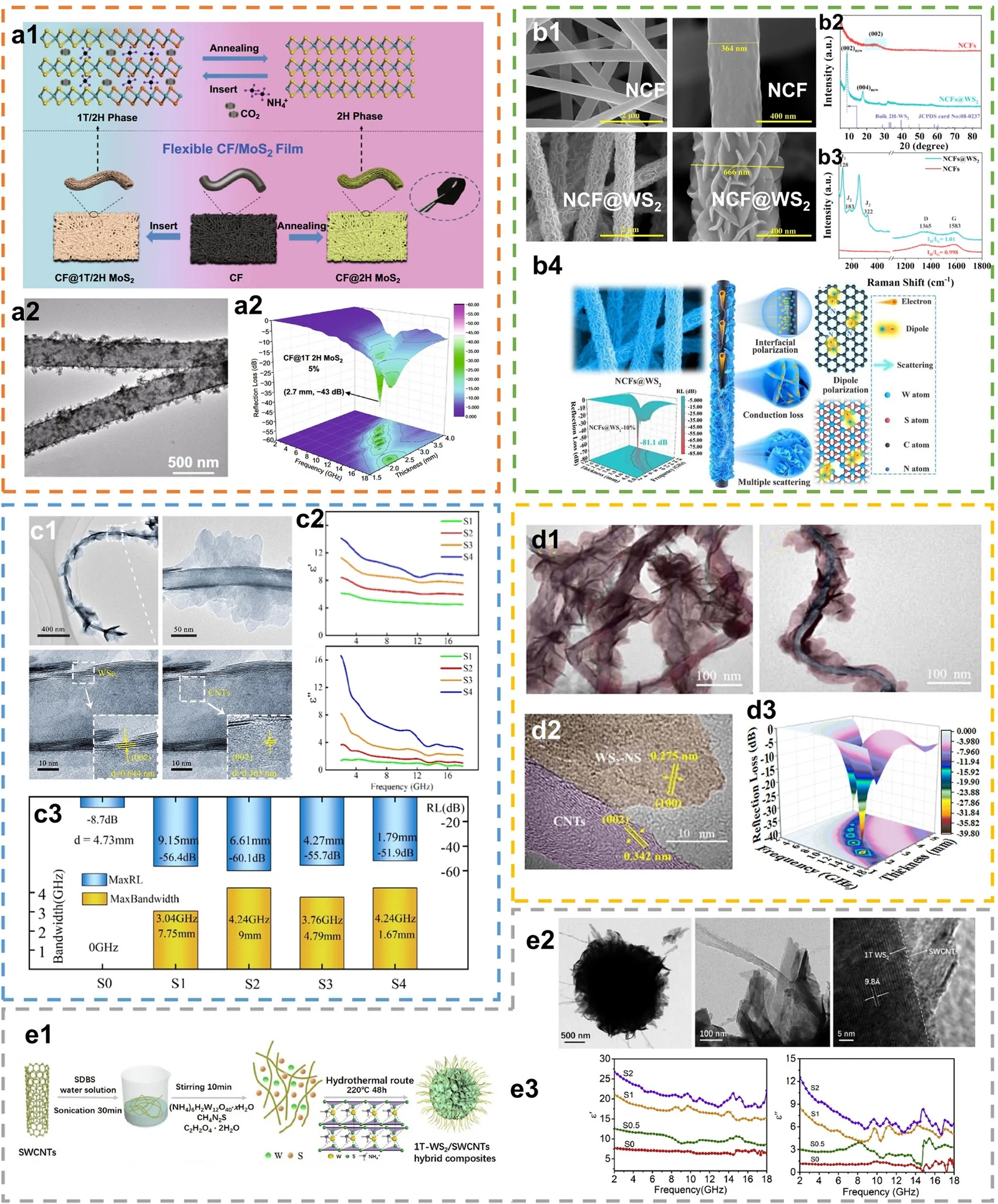
**a1** Schematic illustrating the fabrication of flexible CF@MoS_2_ film; **a2** TEM image of CF@1T/2H MoS_2_ composite; **a3** 3D RL plot of CF@1T/2H MoS_2_ at 5 wt% filling ratio [149]. Copyright 2021 Springer Nature. **b1** SEM morphology image of NCF and NCF@WS_2_; **b2** XRD patterns of NCF and NCF@WS_2_; **b3** Raman spectrum of NCF and NCF@WS_2_; **b4** EMA performance and EMW loss mechanism of NCF@WS_2_ [170]. Copyright 2023 Elsevier. **c1** TEM and HRTEM images of WSe_2_@CNTs; **c2** Permittivity curves of WSe_2_@CNTs samples; **c3** Summary of RL_min_ and EAB of WSe_2_@CNTs samples [171]. Copyright 2022 Elsevier. **d1** WS_2_/CNTs coated structure morphology; **d2** HRTEM image of WS_2_/CNTs materials; **d3** 3D RL plot of WS_2_/CNTs [172]. Copyright 2020 Elsevier. **e1** Schematic of synthesis of 1T-WS_2_/SWCNTs hybrid composites; **e2** TEM images of 1T-WS_2_/SWCNTs; **e3** Permittivity curves of WS_2_/SWCNTs [173]. Copyright 2020 Elsevier

As mentioned above, the sandwich-type atomic configuration intrinsic to TMDs fundamentally constrains their morphological diversity beyond 2D nanosheets and self-assembled spheres, necessitating reliance on secondary components for macroscopic architecture control [169]. The significantly larger dimensions of carbon fibers (CF) establish them as dominant growth substrates, enabling MoS_2_ nanosheets to nucleate and conformally coat their surfaces via van der Waals epitaxy. Huang et al. pioneered a streamlined single step hydrothermal synthesis enabling kilogram scale production of 1T@2H MoS_2_ heterophase architectures via molecular/ionic intercalation (Fig. 9a1) [149]. Structural validation via XRD, Raman, XPS, SEM and TEM confirmed phase specific configurations (Fig. 9a2). The hybrid phase composite with 15 wt% filler loading achieves a RL_min_ of −52.7 dB at 2.6 mm thickness and an ultra-broad EAB of 10.52 GHz, originating from the 1T phase's metallic conductivity that enhances dielectric polarization loss and optimizes impedance matching. Flexible microwave absorbers were also engineered via direct epitaxial growth on CF substrates, achieving −43 dB at only 5 wt% loading (Fig. 9a3). Subsequently, the same team fabricated a WS_2_ nanosheet anchored N doped CF architecture (NCFs@WS_2_) through electrospinning, pre-oxidation, carbonization, and solvothermal growth (Fig. 9b1–b3) [170]. This hierarchical composite delivers a RL_min_ of −81.1 dB at 10 wt% loading and 3.5 mm thickness, and an EAB of 6.24 GHz at 3.0 mm thickness. The superior attenuation arises from multiscale synergistic effects: the 3D conductive network of NCFs provides conduction loss, while 1T phase WS_2_ nanosheets enhance charge migration and dipole polarization via abundant edge sites. N doping and crystalline defects induce dipole polarization that cooperates with WS_2_/NCF interfacial polarization, and the lamellar WS_2_ morphology prolongs electromagnetic propagation paths through multiple scattering (Fig. 9b4). The innovation lies in integrating 1D electrospun CF with 2D TMDs, enabling lightweight flexibility while synergistically enhancing polarization and conduction losses through phase engineering and interface design.

For carbon nanotubes (CNT) with smaller dimensions, TMDs nanosheets tend to grow along the surfaces of CNTs. For instance, Cao et al. embedded 2D WSe_2_ nanosheets into 1D multiwalled CNTs via a solvothermal method (Fig. 9c1), constructing WSe_2_@CNTs nanohybrids that significantly enhanced EMA performance [171]. The CNTs provide a 3D conductive network, while WSe_2_ introduces abundant interfacial and dipole polarization sites. Their synergy mitigates the impedance mismatch of pure CNTs and overcomes the low conductivity limited loss of pure WSe_2_. By adjusting CNT content, tunable permittivity was achieved, as shown in Fig. 9c2. The optimal sample S2 exhibited triple band absorption at 3, 10, and 17 GHz with a total bandwidth of 4.24 GHz at 9 mm thickness. Sample S4 achieved a RL_min_ of −51.9 dB and a bandwidth of 3.84 GHz at 1.79 mm thickness (Fig. 9c3). In addition, the team synthesized an ultrathin WS₂ nanosheet/CNT (WS_2_ NS/CNTs) composite via a one-step hydrothermal method (Fig. 9d1, d2) [170]. Adjusting the CNT content optimizes impedance matching and dielectric loss. The composite with a WS_2_ NS to CNTs molar ratio of 10:1 (S2) delivered a RL_min_ of −51.6 dB at 14.8 GHz and an EAB of 5.4 GHz (12.6–18.0 GHz) at 1.95 mm thickness (Fig. 9d3). Compared to a physically mixed counterpart, the hydrothermally synthesized 3D network enhances EMW attenuation through interfacial polarization, dipole polarization, and multiple scattering [172].

For larger-sized TMD structures, 1D CNT can be embedded to form brush-like architectures. For instance, Cheng et al. successfully synthesized WS_2_ nanoflower/CNT (WS_2_-NF/CNT) hybrid materials via a one-step hydrothermal method (Fig. 9e1, e2) [173]. The unique self-assembled 1D-2D composite structure demonstrates excellent EMA performance. The study revealed that WS_2_ nanoflowers grow uniformly on the surface of CNTs, forming a 3D heterostructure. By adjusting the CNT content, the absorption properties can be effectively optimized. At a minimal thickness of 1.95 mm, the hybrid material achieves an RL_min_ of –51.6 dB at 14.8 GHz, along with an EAB of 5.4 GHz (Fig. 9e3).

As discussed above, the composite of 1D carbon materials with TMDs excels in constructing a long-range conductive network throughout the material. This network, formed by high-aspect-ratio fibers or nanotubes, provides an ideal skeleton for electron migration and conductive pathways, establishing a strong foundation for conductive loss. TMDs nanosheets, anchored precisely onto the surface of the 1D carbon scaffold via in situ growth or loading strategies, not only prevent their own agglomeration but also introduce a multitude of heterojunctions and defects at the interfaces. These interfaces act as polarization centers, generating significant dielectric relaxation, specifically interfacial polarization, under alternating electromagnetic fields, thereby greatly enhancing polarization loss. Concurrently, multiple internal reflections within the 1D structure prolong the dissipation path of EMW. This composite strategy ingeniously combines the rapid conductive loss of the 1D carbon material with the interface/dipole polarization loss of TMDs, achieving a synergistic effect. Its structural attributes make it particularly suitable for macroscopic absorbers requiring lightweight, flexibility, and structural integrity, such as structurally integrated components in aerospace applications.

#### 2D Stacked Heterolayers C/TMDs Composites

2D carbon materials, such as graphene, form layered heterostructures with TMDs through van der Waals forces or chemical bonding. The key advantages of these composites lie in their multidimensional interfacial polarization and structural design flexibility. The high specific surface area and flexibility of graphene provide an ideal substrate for the uniform growth of TMDs. The alternating stacking of these two components creates multiple heterointerfaces, significantly enhancing interfacial polarization and promoting charge redistribution. Furthermore, the semiconducting nature of TMDs and the high electrical conductivity of graphene synergistically modulate the complex permittivity, enabling broadband impedance matching. These materials exhibit outstanding performance in low-frequency bands. However, the interlayer stacking density and interfacial bonding strength critically influence the dominant loss mechanisms.

**Fig. 10:**
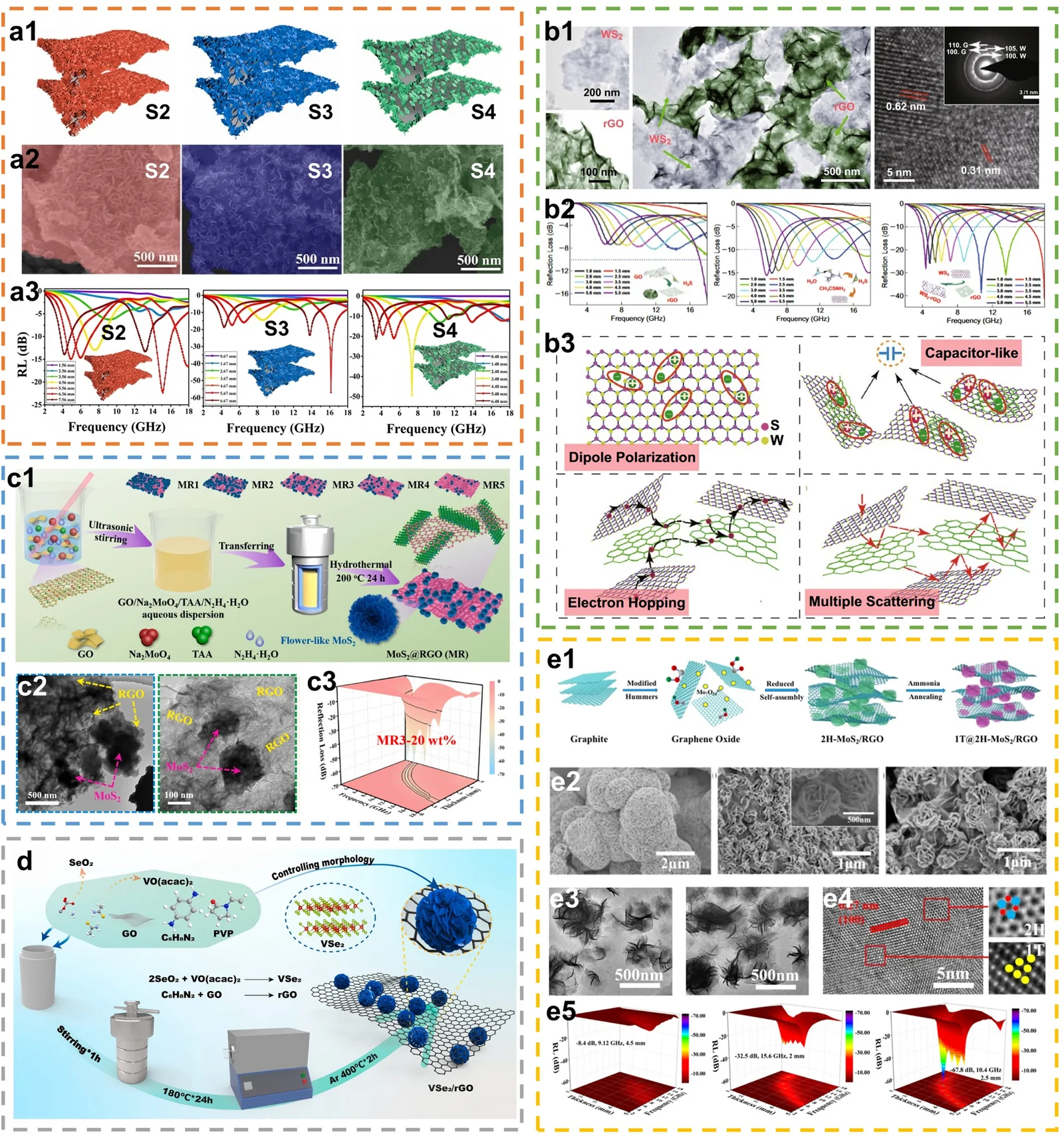
**a1** Schematic diagram of the 2D MoSe_2_@RGO vertically implanting structure; **a2** SEM images of MoSe_2_@RGO with different ratio; **a3** RL curves of MoSe_2_@RGO samples [174]. Copyright 2022 Elsevier. **b1** TEM images and SEAD patterns of WS_2_-RGO; **b2** RL curves of RGO, WS_2_, WS_2_-RGO, respectively; **b3** Synergistic EMA mechanisms in WS_2_-RGO heterostructure [38]. Copyright 2019 Springer Nature. **c1** Synthesis process schematic of flower-like MoS_2_@RGO; **c2** TEM images of MoS_2_@RGOs samples; **c3** 3D RL plot of MR3 at 20 wt% [175]. Copyright 2025 Elsevier. **d** Schematic diagram of the design process for VSe_2_/RGO composite [176]. Copyright 2025 Elsevier. **e1** Schematic diagram of the synthetic process of the MoS_2_/RGO system; **e2** SEM images of 2H-MoS_2_, 2H-MoS_2_/RGO, 1T@2H-MoS_2_/RGO, respectively; **e3** TEM images of 1T@2H-MoS_2_/RGO; **e4** HRTEM images of 1T@2H-MoS_2_; **e5** 3D RL plots of 2H-MoS_2_, 2H-MoS_2_/RGO, 1T@2H-MoS_2_/RGO, respectively [177]. Copyright 2020 American Chemical Society

Fu et al. enhanced low frequency EMA performance (S, X, Ku bands) by constructing vertically aligned MoSe_2_ nanosheets on reduced graphene oxide (RGO) (Fig. 10a1) [174]. The hybrid leverages RGO's conductive network and MoSe_2_'s narrow bandgap for synergistic conductive and polarization loss (Fig. 10a2). Multiple relaxation effects and hierarchical structure prolong EMW paths, achieving a RL_min_ of −56.9 dB at 8.9 mm and an EAB of 4.12 GHz (Fig. 10a3). This study offers a new strategy for developing lightweight microwave absorbers with multi-band low-frequency response through interface engineering between 2D carbon materials and TMDs. Zhang et al. constructed a WS_2_‑RGO heterostructure via hydrothermal method (Fig. 10b1) [38]. Exceptional EMA properties arise from four mechanisms: sulfur vacancies and oxygen defects for dipole polarization; WS_2_/RGO interfaces for interfacial polarization; RGO conductive network for conductive loss; and large surface area for multiple scattering. The material achieves a RL_min_ of −41.5 dB at 9.5 GHz (2.7 mm) and an ultra‑wide EAB of 13.62 GHz at 1.7 mm (Fig. 10b2).

For larger-sized self-assembled TMD spheres, a coated or supported architecture can be constructed with RGO nanosheets, effectively enhancing interfacial polarization and optimizing impedance matching characteristics. Guo et al. [175] reported a heterostructured composite material comprising 2D RGO encapsulating flower-like MoS_2_ (Fig. 10c1). By tuning the mass ratio of MoS_2_ to RGO, with an optimal ratio of 1:1, the composite achieved a RL_min_ of −69.6 dB at 8.46 GHz and a broad EAB of 4.36 GHz (ranging from 11.00 to 15.36 GHz) at a low filler loading of 20 wt% (Fig. 10c3). The incorporation of 2D RGO not only enhanced dielectric loss through the formation of a conductive network but also extended the EMW propagation path via multiple reflections enabled by the surface-supported flower-like MoS_2_ nanosheets (Fig. 10c2). Ma et al. [176] found that single component VSe_2_ suffers from impedance mismatch despite its high conductivity. To solve this, they introduced RGO and tuned the VSe_2_ morphology from flower like to exfoliated nanosheets by adjusting PVP, which optimized interfacial polarization and charge transfer (Fig. 10d). The optimal sample achieved a RL_min_ of –79.50 dB at 2.01 mm and an EAB of 5.2 GHz at 1.45 mm, demonstrating that RGO/TMDs synergy balances dielectric loss and impedance matching. Guo et al. [177] used nitrogen doping to induce phase modulation in MoS_2_/RGO composites (Fig. 10e1). Rose like MoS_2_ nanoflowers were grown on RGO and calcined in ammonia, forming a multi-interface heterostructure (Fig. 10e2‑–4). The composite delivered an EAB of 4 GHz and a RL_min_ of –67.77 dB (Fig. 10e5). The EMA performance gains arise from three synergies: RGO/1T‑MoS_2_ conductive network, hierarchical structure for multiple scattering, and N‑doping-induced polarization sites.

The composite of 2D RGO with TMDs represents a classic "face-to-face" contact paradigm and a model of synergistic enhancement. RGO, with its enormous specific surface area and excellent intrinsic conductivity, provides an extremely expansive and stable support platform for TMDs, effectively suppressing the restacking of both nanosheets and thus maximizing the exposure of active sites. When the two are stacked alternately to form a layered heterostructure, intense Maxwell–Wagner-Sillars interfacial polarization occurs at their intimately contacted interfaces, which is one of the core loss mechanisms in this system. Furthermore, the potential Fermi level difference between RGO and TMDs promotes charge rearrangement at the interface, forming a built-in electric field that further facilitates charge transfer and relaxation processes [78]. The coupling of their energy band structures may also introduce additional dipole polarization. This profound synergy achieved within the 2D plane enables the composite to simultaneously possess excellent conductive loss, strong polarization loss, and readily tunable impedance matching. Consequently, 2D RGO/TMDs composites often demonstrate exceptional dielectric loss performance and a high *α*, holding great potential for thin-layer coating applications that demand highly efficient electromagnetic energy conversion.

#### 3D Hierarchical Architectures C/TMDs Composites

**Fig. 11:**
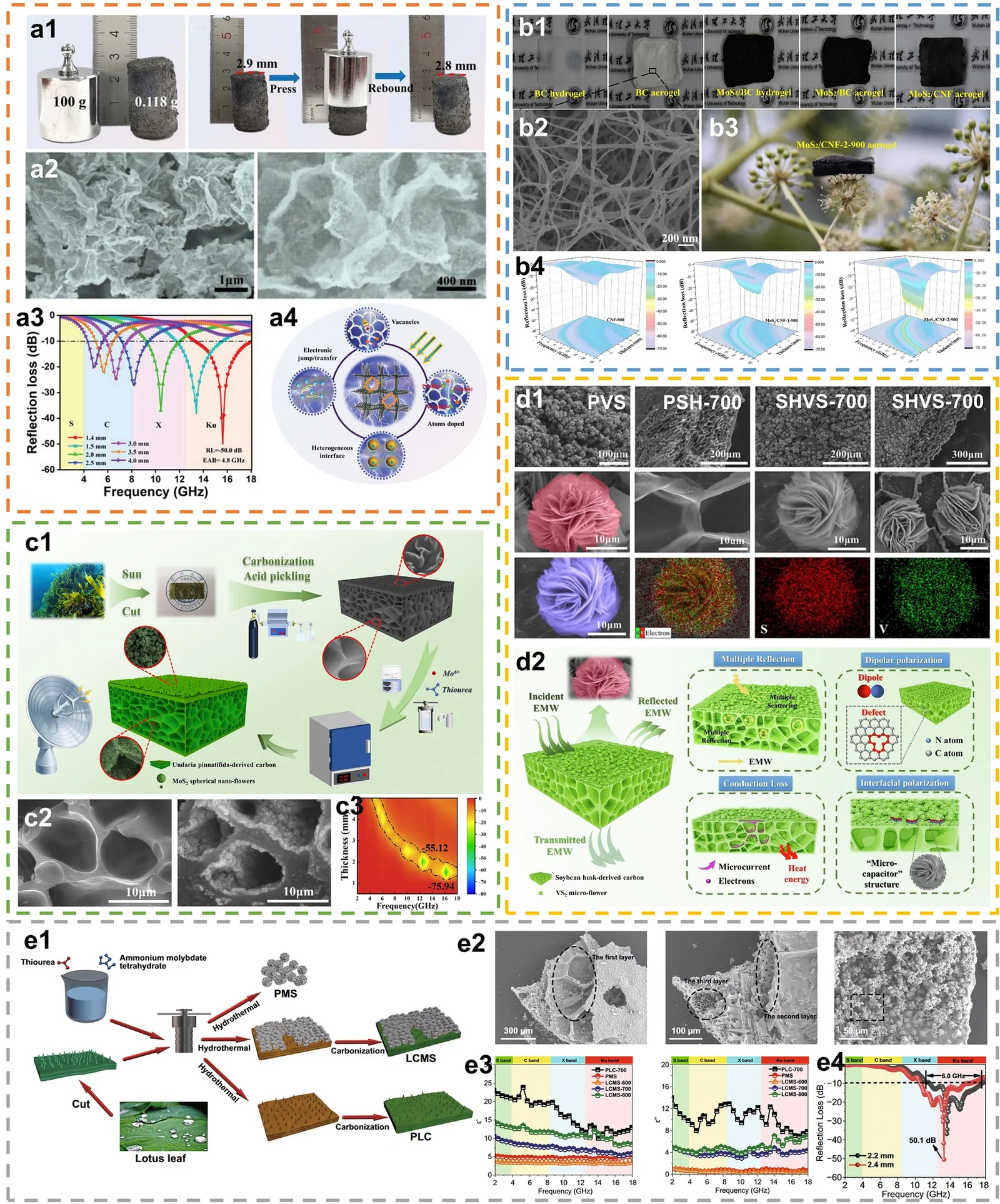
**a1** Macroscopic images of the N-RGO/CoSe_2_ and its press and rebound characteristic; **a2** SEM images of N-RGO/CoSe_2_; **a3** RL curves of N-RGO/CoSe_2_ samples; **a4** Schematic illustration of EMW dissipation mechanisms in N-RGO/CoSe_2_ composites [180]. Copyright 2023 Wiley-VCH. **b1** Physical appearance of BC hydrogel, BC aerogel, MoS_2_/BC hydrogel, MoS_2_/BC aerogel, MoS_2_/CNF aerogel, respectively; **b2** SEM image of BC aerogel; **b3** Digital photograph of MoS_2_/CNF-2-900 aerogel; **b4** 3D RL plots of CNF-900, MoS_2_/CNF-1-900, MoS_2_/CNF-2-900, respectively [163]. Copyright 2022 Elsevier. **c1** Schematic illustration for the fabrication of undaria pinnatifida derived porous C/MoS_2_ hybrid materials; **c2** SEM images of porous C and UCMS-800, respectively; **c3** 2D RL plot of UCMS-800 [179]. **d1** SEM images of PVS, PSH-700, the surface and cross section of SHVS-700 (top), the enlarged versions of each of these (middle), the EDS mapping of SHVS-700 (bottom); **d2** Synergistical EMA mechanism of SHVS-700 [37]. Copyright 2022 Elsevier. **e1** Schematic illustration of the GHPCM formation process; **e2** SEM images of LCMS-700 with typical multilayer structure; **e3** Permittivity curves of different samples; **e4** RL curves of LCMS-700 [181]. Copyright 2021 Springer Nature

As discussed in Sect. 4.3, the growth kinetics of TMDs do not favor the spontaneous formation of large-scale 3D hierarchical structures, where "large-scale" refers to dimensions significantly beyond the several-micrometer size of their self-assembled microspheres. To achieve such 3D architectures, TMDs typically require integration with other materials, such as graphene [178], nanofibers [163], or biomass-derived carbon [179]. In an innovative approach, He et al. designed a 3D hierarchical CoSe_2_/N‑doped RGO composite (Fig. 11a1) [180]. The key innovation lies in the multifunctional role of N‑RGO: it acts as a porous scaffold (surface area 358.6 m^2^ g^−1^) to uniformly disperse CoSe_2_ nanoparticles (5–20 nm, Fig. 11a2), while N doping introduces defects that enhance interfacial polarization. Moreover, a Schottky heterojunction forms between N‑RGO (work function 3.97 eV) and CoSe_2_ (5.15 eV), facilitating charge transfer and energy dissipation. As a result, the composite achieves an outstanding EAB of 4.8 GHz at an ultra‑thin thickness of only 1.4 mm, with a density merely 1/500 that of steel (Fig. 11a3). This work demonstrates that combining 3D carbon frameworks with TMDs can simultaneously address thickness, weight, and performance challenges. Dong et al. [163] fabricated a 3D porous MoS_2_/carbon nanofiber aerogel using bacterial cellulose as a template (Fig. 11b1). Notably, adjusting the MoS_2_/CNF mass ratio led to two distinct microstructures: flower‑like MoS_2_ spheres anchored on CNFs, or a core‑shell structure where CNFs are encapsulated by MoS_2_ nanosheets (Fig. 11b2, b3). This hierarchical architecture effectively prevents MoS_2_ agglomeration and provides multiple loss mechanisms, including conduction loss, polarization loss, and multiple scattering. Impressively, the MoS_2_/CNF‑2‑900 sample with only 5 wt% filler loading exhibits an EAB of 5.68 GHz at a thin thickness of 2.0 mm (Fig. 11b4), highlighting the potential of ultralight aerogels for efficient absorption.

Professor Lu and colleagues reported a biomass‑derived MoS_2_/kelp porous carbon composite (UCMS) [179]. The unique aspect is the use of kelp’s natural honeycomb pore structure as a template, followed by in situ growth of MoS_2_ nanoflowers both on the surface and inside the enclosed pores (Fig. 11c2). This is the first time that confined pore loading of MoS_2_ has been achieved in biomass carbon. The resulting 3D hierarchical design offers abundant interfaces, a conductive network, and multiple scattering. The optimized UCMS‑800 reaches an RL_min_ of –75.94 dB at just 1.68 mm and an EAB of 4.12 GHz (Fig. 11c3), showcasing sustainable, lightweight, and high‑efficiency absorption. Subsequently, the same team fabricated an N‑doped carbon/VS_2_ composite (SHVS) from waste bean hulls (Fig. 11d1) [37]. The bean‑derived carbon retains a honeycomb porous structure, and VS_2_ microflowers are uniformly anchored on the carbon framework. Nitrogen doping further introduces defect sites. This synergy provides three benefits: a 3D conductive network for conduction loss, improved impedance matching due to VS_2_, and enhanced interfacial/dipole polarization (Fig. 11d2). The optimized SHVS‑700 achieves an RL_min_ of –62.74 dB at 1.7 mm and a full Ku‑band EAB of 6.04 GHz, demonstrating that agricultural waste can be valorized into high‑performance absorbers. Furthermore, a gradient hierarchical porous C/MoS_2_ composite was reported, fabricated via a lotus leaf morphology-genetic strategy (Fig. 11e1) [181]. The leaf’s inherent gradient pore channels and Janus (hydrophobic/hydrophilic) surfaces guide MoS_2_ to form flower‑like structures on one side and nanosheets on the other, preserving the bio‑template’s full hierarchical porosity (Fig. 11e2). This structural design results in triple synergy: conductive loss from the carbon network, interfacial polarization at MoS_2_/C heterojunctions, and multiple reflections within the multilevel pores. Consequently, the material yields an RL_min_ of −50.1 dB at 2.4 mm and an ultra‑wide EAB of 6.0 GHz (11.6–17.6 GHz) at 2.2 mm (Fig. 11e3).

The composite of 3D hierarchical porous carbon with TMDs elevates material design from the level of composition and interfaces to that of macroscopic spatial architecture. Its superiority stems from the meticulously designed multiscale pore system comprising macropores, mesopores, and micropores. This open 3D network structure firstly acts as an efficient "EMW trap," significantly prolonging the propagation path of EMW through multiple reflections and scattering, thereby increasing their probability of being dissipated. Simultaneously, the enormous specific surface area and abundant porosity provide an ideal environment for the high-density and highly dispersed loading of TMDs, effectively preventing their aggregation and exposing numerous polarization sites. In this architecture, pores of different scales serve distinct functions: macropores and mesopores primarily manage the propagation and reflection of waves, optimizing impedance matching; micropores mainly induce dipole polarization. The graphitized carbon walls forming the skeleton provide conductive loss. The introduction of TMDs further enhances interfacial and defect polarization. This multi-level synergistic mechanism achieved within 3D space, perfectly unifies the contradictory requirements of good impedance matching and strong attenuation capability. It represents an ideal model for achieving high-performance EMA characterized by "strong absorption, broad bandwidth, small thickness, and low weight."

In summary, the key feature of C/TMDs composites lies in the effective synergy between the highly conductive network provided by carbon component and the tunable dielectric properties of TMDs, enabling a desirable trade‑off between loss capability and impedance matching. C/TMDs scaffolds of different dimensions offer distinct advantages. 1D fiber-like C/TMDs readily forms long‑range continuous conductive networks, contributing predominantly to conductive loss. 2D layered C/TMDs provides large‑area heterointerfaces, giving the strongest interfacial polarization, though restacking remains a concern. 3D C/TMDs skeletons combine ultralight weight with multiple reflection and scattering, yet they often suffer from low mechanical strength, complex preparation, and poor batch‑to‑batch reproducibility. Current research trends suggest that cross‑dimensional synergistic designs integrating 1D conductive networks, 2D heterointerfaces, and 3D porous pathways represent a promising route toward high‑performance C/TMDs absorbers, although rational matching among these dimensions requires further investigation.

### Other Dielectric Materials/TMDs Composites

Following carbon-based composites, the integration of TMDs with emerging dielectric materials such as MXene, metal sulfides, and oxides opens a new frontier in the design of EMA materials. Compared to carbon matrices, these dielectric components offer a more versatile platform for fine-tuning the composite's permittivity. More importantly, they enable synergistic optimization of impedance matching and loss capabilities through diversified polarization mechanisms and enhanced interfacial effects [182, 183]. This multicomponent strategy effectively overcomes the limitations of single-component systems, achieving highly efficient EMW attenuation and broad frequency band absorption at reduced thicknesses through sophisticated synergy, thereby demonstrating significant application potential.

#### MXene/TMDs Composites

**Fig. 12:**
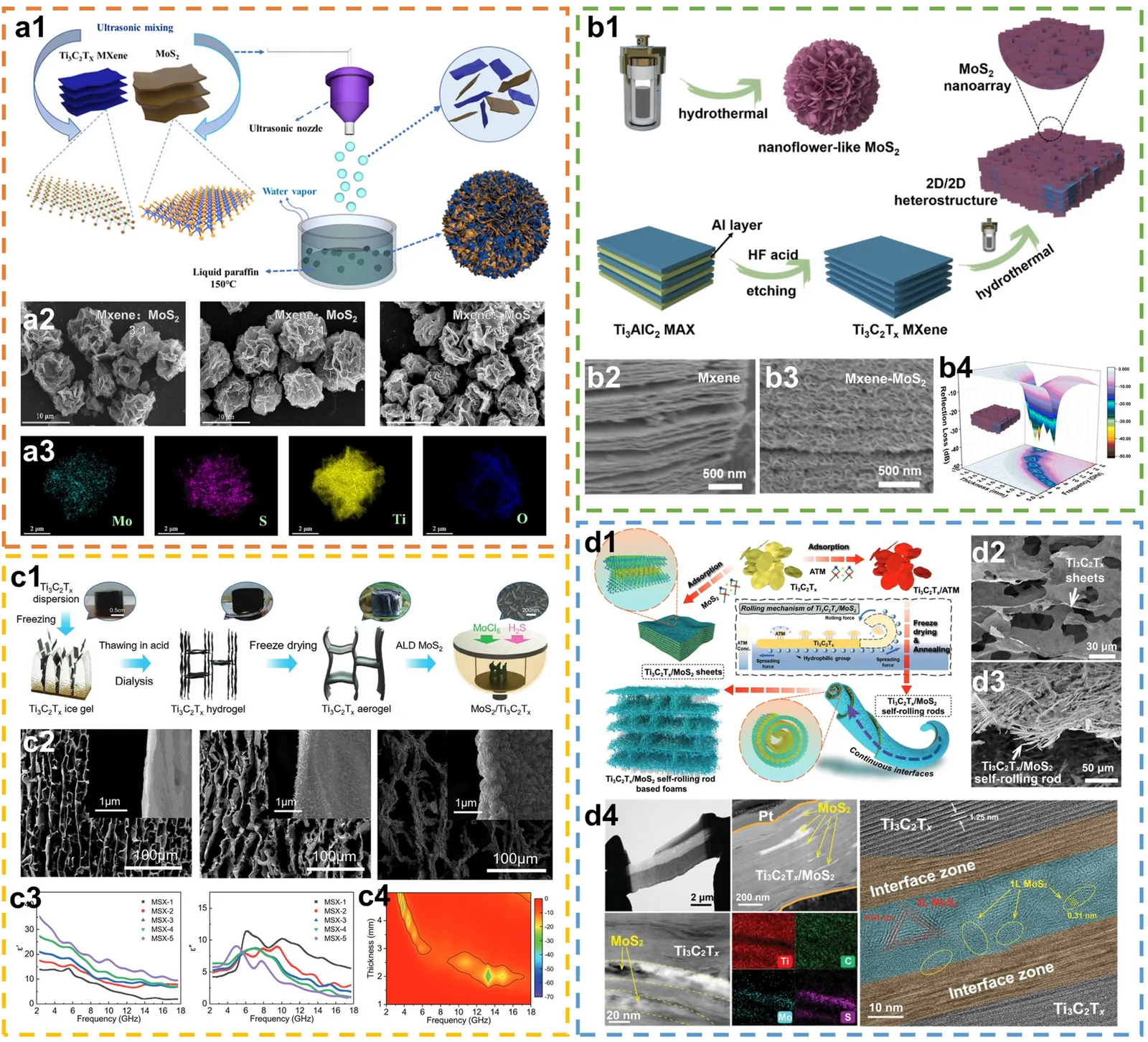
**a1** Schematic illustration of preparation process of MXene/MoS_2_; **a2** SEM images of MXene/MoS_2_ samples; **a3** EDS mappings of MXene/MoS_2_ [185]. Copyright 2022 Elsevier. **b1** Schematic diagram of the preparation process of MXene-MoS_2_ heterostructures; SEM image of **b2** MXene and **b3** MXene-MoS_2_; **b4** 3D RL plot of MXene-MoS_2_ [186]. Copyright 2021 Elsevier. **c1** Schematic diagram of the preparation process of MoS_2_/MXene hybrid aerogel; **c2** SEM images of Ti_3_C_2_T_*x*_, MoS_2_/MXene with 200 ALD cycles, and 500 ALD cycles, respectively; **c3** Permittivity curves of MSX-x samples; **c4** 2D RL plots of MSX-3 [164]. Copyright 2022 Wiley-VCH. **d1** Schematic diagram of the fabrication of Ti_3_C_2_T_*x*_/MoS_2_ sheets and rolling mechanism of Ti_3_C_2_T_*x*_/MoS_2_ self-rolling rods; SEM images of **d2** Ti_3_C_2_T_*x*_ sheets and **d3** Ti_3_C_2_T_*x*_/MoS_2_ self-rolling rods; **d4** TEM and HRTEM images of Ti_3_C_2_T_*x*_/MoS_2_ [187]. Copyright 2022 Wiley-VCH

The multilayered 2D structure and abundant surface functional groups of MXene materials enable them to maintain low density while significantly enhancing interfacial and dipole polarization losses. Furthermore, their hierarchical architecture promotes multiple reflections and scattering of EMW [184]. However, the high conductivity and high permittivity characteristic of MXene can readily lead to impedance mismatch. Compounding MXene with semiconducting TMDs such as MoS_2_ effectively mitigates this issue by balancing the impedance, prolonging the EMW propagation path, and increasing the dissipation efficiency. Zhang and colleagues prepared MXene/MoS_2_ composite microspheres via an innovative one‑step ultrasonic spray technique (Fig. 12a1) [185]. With an optimal MXene/MoS_2_ mass ratio of 5:1, the nanosheets self‑assembled into wrinkled microspheres (~ 8 µm diameter) through π–π interactions (Fig. 12a2), increasing the specific surface area to 78.2 m^2^ g^−1^ and creating abundant mesopores. Elemental mapping confirmed uniform distribution of Mo, S, Ti, and O (Fig. 12a3). As a result, the composite achieved a RL_min_ of –51.2 dB at 2.5 mm. Notably, this self‑assembly strategy avoids the need for templates and offers a scalable route. Alternatively, MoS_2_ can be grown directly onto MXene, Li et al. hydrothermally grew 2D MoS_2_ nanosheets on multilayered MXene substrates, constructing a 2D/2D heterostructure with a 3D conductive network (Fig. 12b1) [186]. The vertical growth of MoS_2_ effectively mitigates MXene restacking and expands its interlayer spacing (Fig. 12b2, b3). The resulting synergy provides multiple scattering paths from MXene and enhanced interfacial polarization/conduction loss from MoS_2_. The composite achieved a RL_min_ of –46.72 dB at only 2 mm and an EAB of 4.32 GHz (Fig. 12b4).

Yang et al. employed atomic layer deposition to coat MoS_2_ onto a 3D porous Ti_3_C_2_T_*x*_ MXene aerogel template (Fig. 12c1) [164]. The aerogel was first prepared with a long‑range aligned structure via ice‑templating. With increasing ALD cycles, MoS_2_ evolved from discrete nanosheets into a continuous fibrous network while preserving the porous skeleton (Fig. 12c2). The composite showed interesting frequency‑dependent permittivity: the real part increased from 7.1 to 18.3, while the imaginary part decreased from 7.6 to 4.2 (Fig. 12c3), indicating optimized impedance matching via heterointerfaces. The sample with 300 cycles achieved a RL_min_ of –61.65 dB at 4.53 mm and an EAB of 5.9 GHz (Fig. 12c4). Li et al. [187] developed a Ti_3_C_2_T_*x*_/MoS_2_ self‑rolled rod‑like foam (Fig. 12d1). Surface tension differences induced self‑rolling, combined with unidirectional freeze‑drying and heat treatment. The foam exhibited an ordered layered structure with interlayer spacing ~ 200 µm, composed of intersecting rod‑like units (Fig. 12d2, d3). MoS_2_ was uniformly distributed as thin sheets (~ 10 nm) between Ti_3_C_2_T_*x*_ layers, forming curved heterointerfaces (Fig. 12d4). This unique microstructure generated interfacial polarization accounting for over 80% of total dielectric loss. Consequently, the material achieved effective absorption across the entire X‑band (8.2–12.4 GHz) at an ultra‑low density of 0.009 g cm^−3^ and 3.3 mm thickness.

In summary, MXene/TMDs composites offer unique synergistic advantages: MXene provides a conductive loss network, while TMDs serve as dielectric modulation units introducing polarization relaxation. Their intimate heterointerfaces optimize impedance matching and enhance dielectric loss via charge rearrangement. Future research should focus on precise interfacial engineering, stable 3D macro‑assembly to avoid stacking, and structure‑property modeling. However, challenges remain, including MXene's environmental instability, complex and costly preparation, and insufficient understanding of multi‑loss synergy. Addressing these fundamental science and engineering technology issues will be crucial for advancing these composites toward practical applications.

#### Metal Sulfides/TMDs Composites

**Fig. 13:**
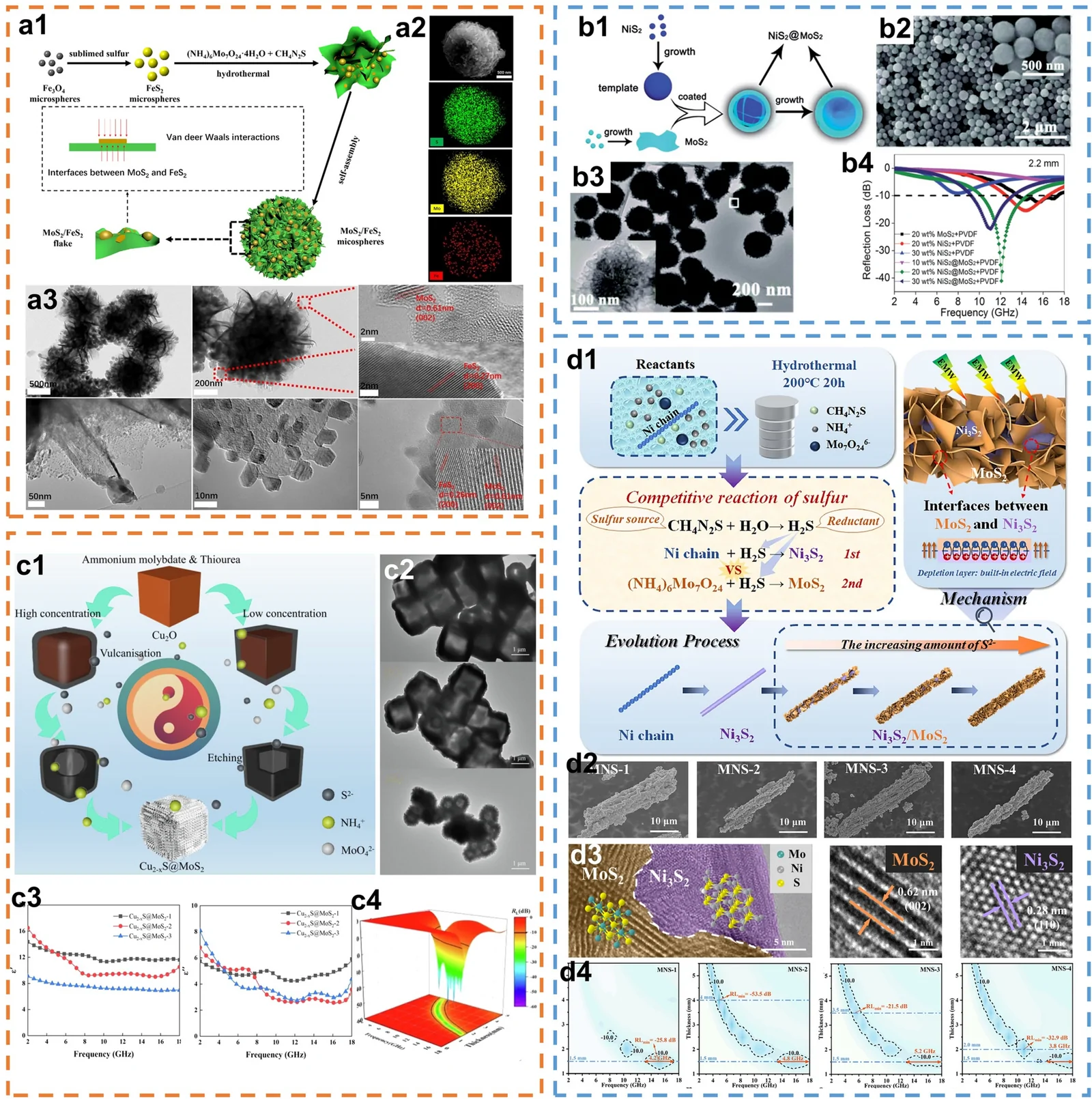
**a1** Schematic diagram of the synthetic process of MoS_2_/FeS_2_ composites; **a2** EDS mappings of FSM3; **a3** TEM and HRTEM images of FSM3 [90]. Copyright 2020 Elsevier. **b1** Schematic diagram of the synthesis of the NiS_2_@MoS_2_ nanospheres; **b2** SEM images of NiS_2_ nanospheres; **b3** TEM images of NiS_2_@MoS_2_ nanospheres; **b4** RL curves of different samples [188]. Copyright (2017) Royal Society of Chemistry. **c1** Schematic illustration of the formation mechanism of Cu_2-*x*_S@MoS_2_; **c2** TEM images of Cu_2−*x*_S@MoS_2_-1, Cu_2−*x*_S@MoS_2_-2, Cu_2−*x*_S@MoS_2_-3, respectively; **c3** Permittivity curves of Cu_2−*x*_S@MoS_2_ samples; **c4** 3D RL plots of Cu_2−*x*_S@MoS_2_-3 [189]. Copyright 2023 Elsevier. **d1** Schematic diagram of the fabrication, the competitive reaction of sulfur, and the evolution mechanism of MoS_2_/Ni_3_S_2_; **d2** SEM images of prepared MoS_2_/Ni_3_S_2_ samples **d3** HRTEM images of MoS_2_/Ni_3_S_2_; **d4** 2D RL plots of MNS-x [190]. Copyright 2024 Elsevier

Compared to composites incorporating carbon or MXene, dielectric metal sulfide/TMDs hybrids offer distinct advantages for EMA. The primary benefits stem from the high polarizability and tunable dielectric properties of metal sulfides. When combined with TMDs, these features effectively enhance dielectric loss and improve impedance matching. The formation of a semiconducting heterojunction between the components creates a built-in electric field, which significantly boosts dielectric polarization and thereby optimizes the overall absorption capability. Furthermore, the 2D structure and high conductivity of TMDs, coupled with the diversity of metal sulfides, enable fine-tuning of loss mechanisms, such as interface and dipole polarization. Xin et al. reported a 3D hierarchical MoS_2_/FeS_2_ composite with a localized face‑to‑face connected heterojunction structure (Fig. 13a1) [90]. SEM–EDS confirmed the presence and uniform distribution of the Fe and Mo elements (Fig. 13a2). HRTEM revealed lattice fringes of MoS_2_ (0.61 nm, (002) plane) and FeS_2_ (0.27 nm, (200) plane) (Fig. 13a3). Crucially, electron holography verified the formation of an intimate Ohmic contact and a localized electric field at the heterojunction interface, which significantly boosts interfacial polarization loss. As a result, the composite achieves an exceptional RL_min_ of −60.2 dB at 2 mm thickness and an EAB of 6.48 GHz, demonstrating that well‑designed heterojunctions can dramatically enhance absorption. Zhang et al. developed core‑shell NiS_2_@MoS_2_ nanospheres via a facile hydrothermal method [188]. NiS_2_ nanospheres were first synthesized solvothermally as cores, then MoS_2_ nanosheets grew on them to form a uniform core‑shell architecture (Fig. 13b1). The spheres have diameters of 200–300 nm, with a MoS_2_ shell thickness of ~ 25 nm composed of wrinkled nanosheets (Fig. 13b2, b3). Notably, the core‑shell design provides abundant heterointerfaces that enhance interfacial polarization. With 20 wt% filler loading, the composite achieves a RL_min_ of −41.05 dB at 12.08 GHz (2.2 mm) and an EAB of 4.4 GHz (Fig. 13b4, b5), highlighting the effectiveness of core‑shell engineering for TMDs‑based absorbers.

Huang et al. designed hollow Cu_2−*x*_S@1T‑MoS_2_ micro‑cubes using Cu_2_O cubes as self‑sacrificing templates (Fig. 13c1) [189]. During the hydrothermal process, MoS_2_ nanosheets grew vertically on the Cu_2−*x*_S micro‑boxes, forming a core‑shell architecture that prevents MoS_2_ agglomeration (Fig. 13c2). The high conductivity of the metallic 1T phase, the hollow conductive network, and interfacial/dipole polarization work together (Fig. 13c3). At 40 wt% loading, the composite achieves a RL_min_ of −52.2 dB at 2.1 mm and an EAB of 4.7 GHz (Fig. 13c4), demonstrating that hollow templates combined with phase engineering can effectively balance impedance matching and attenuation. Luo et al. constructed a 1D MoS_2_/Ni_3_S_2_ core‑shell composite via a one‑step hydrolysis method using nickel chains as templates [190]. By adjusting the thiourea amount, they precisely controlled the MoS_2_ shell thickness. MoS_2_ nanosheets uniformly grew on Ni_3_S_2_ nanorods, forming a cable‑like structure (Fig. 13d2). HRTEM showed lattice fringes of MoS_2_ (0.62 nm, (002)) and Ni_3_S_2_ (0.28 nm, (110)) with intimate contact (Fig. 13d3). Electron holography revealed charge redistribution at the heterointerface, enhancing interfacial polarization. As a result, the composite achieves a RL_min_ of −53.5 dB and an EAB of 4.8 GHz at only 1.5 mm thickness (Fig. 13d4), highlighting the advantage of 1D core‑shell designs for thin and strong absorption.

These metal sulfide and TMDs composites demonstrate unique synergistic enhancement in dielectric properties. Their fundamental commonality lies in providing moderate intrinsic electrical conductivity, effectively avoiding the impedance mismatch often caused by highly conductive components. These sulfides significantly enhance interfacial polarization through the formation of intimate heterointerfaces with TMDs. Inherent lattice defects and vacancies in the materials further promote dipole polarization and charge redistribution processes. Future research pivots on precisely regulating the phase composition and microstructure of sulfides to optimize their intrinsic electromagnetic parameters. Deepening the understanding of charge transfer and energy dissipation mechanisms at heterointerfaces is crucial for advancing material design. Current challenges primarily involve the insufficient chemical stability of some metal sulfides in complex environments, limiting their long-term service life. The controllable construction of macroscopic structures and scalable fabrication processes remain bottlenecks for practical application. A more systematic theoretical interpretation of EMW attenuation pathways and dominant mechanisms in multicomponent systems is still required.

#### Low-Conductivity Dielectric Materials /TMDs Composites

**Fig. 14:**
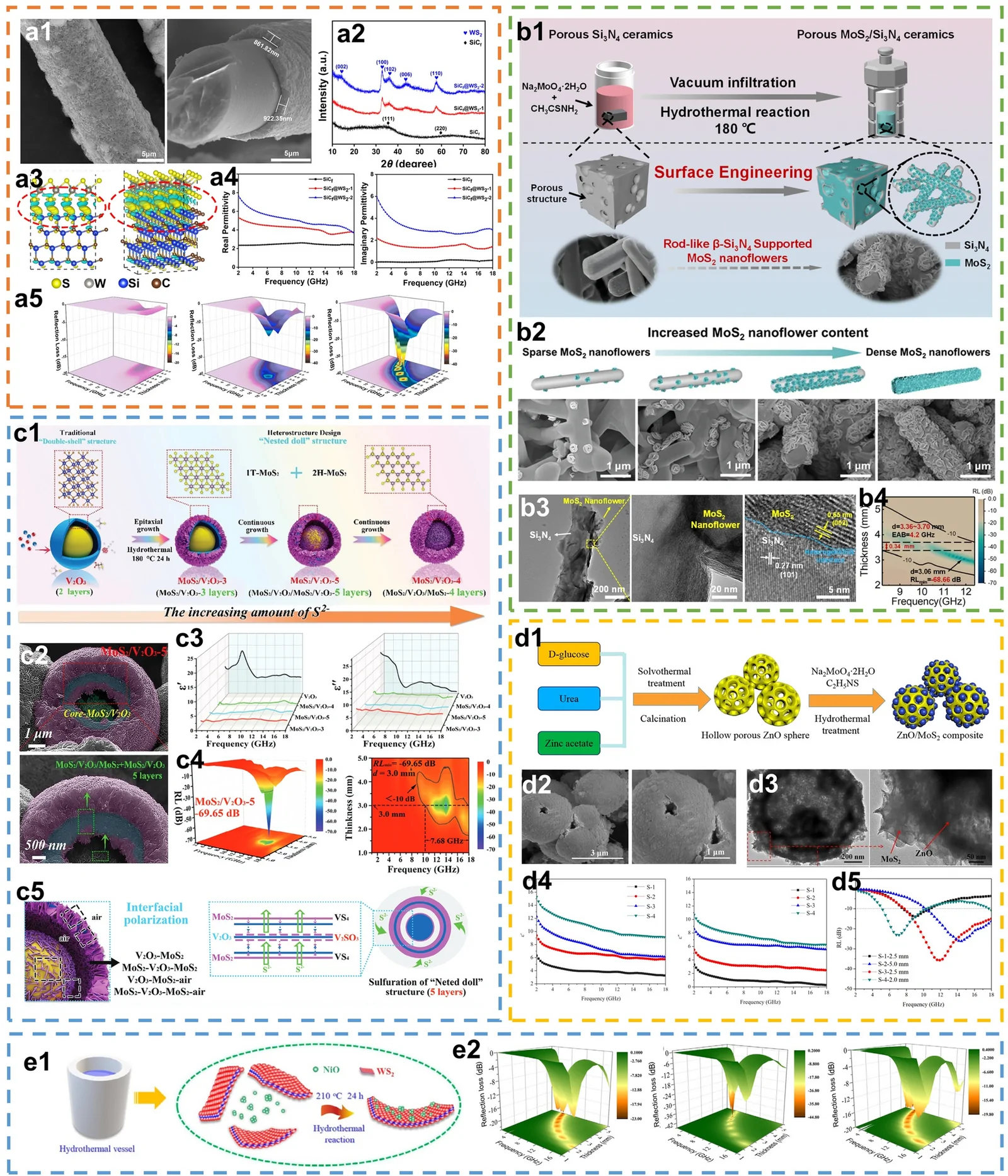
**a1** SEM image of SiC_f_@WS_2_-2 with typical core–shell structure; **a2** XRD patterns of SiC_f_, and SiC_f_@WS_2_ samples; **a3** Differential charge density distributions at hetero-interfaces; **a4** Permittivity curves of SiC_f_, and SiC_f_@WS_2_ samples; **a5** 3D RL plots of SiC_f_, and SiC_f_@WS_2_ samples [192]. Copyright 2023 Elsevier. **b1** Schematic diagram of the synthesis of porous MoS_2_/Si_3_N_4_ ceramics; **b2** SEM images of MoS_2_/Si_3_N_4_-0.70, MoS_2_/Si_3_N_4_-1.05, MoS_2_/Si_3_N_4_-1.40 and MoS_2_/Si_3_N_4_-1.75, respectively; **b3** TEM images of MoS_2_/Si_3_N_4_-1.40; **b4** 2D RL plot of MoS_2_/Si_3_N_4_-1.40 [193]. Copyright 2023 Royal Society of Chemistry. **c1** Schematic illustration of the controllable synthesis of “double-shell” structured V_2_O_3_ and “nested doll” structured MoS_2_/V_2_O_3_ absorbers; **c2** SEM images of MoS_2_/V_2_O_3_-3; **c3** Permittivity curves of MoS_2_/V_2_O_3_-x samples; **c4** 3D and 2D RL plots of MoS_2_/V_2_O_3_-5; **c5** Schematic illustration of the interfacial polarization in MoS_2_/V_2_O_3_ [27]. Copyright 2024 Wiley-VCH. **d1** Schematic representation of the synthesis of the ZnO/MoS_2_ composite; **d2** SEM images of prepared ZnO/MoS_2_ samples **d3** TEM images of prepared ZnO/MoS_2_; **d4** Permittivity curves of ZnO/MoS_2_ samples; **d5** RL curves of different samples [194]. Copyright 2020 Elsevier. **e1** Synthesis illustration for the WS_2_/NiO hybrids with heterostructures; **e2** 3D RL plots of WS_2_/NiO-1, WS_2_/NiO-2, WS_2_/NiO-3, respectively [195]. Copyright 2020 Elsevier

Beyond these, composites combining TMDs with various low-conductivity dielectric materials present a distinct advantage: the synergistic achievement of excellent impedance matching and effective loss mechanisms [191]. In such systems, the dielectric component primarily optimizes the incident EMW for penetration, while the TMDs dominate the dielectric loss through interfacial and dipole polarization. This configuration effectively avoids the charge shielding effect that can occur prematurely with highly conductive carriers. As a result, these composites can achieve stronger EMA over a broader frequency range and at a reduced thickness. Zhao et al. synthesized a 1D@2D heterostructure of SiC fibers coated with WS_2_ nanosheets via a hydrothermal method (Fig. 14a1) [192]. The coating thickness was controllable between 0.8 and 1 µm. XRD confirmed β‑SiC and 2H‑WS_2_ (Fig. 14a2). DFT calculations revealed that electron transfer from WS_2_ to SiC via C–W bonds enhances both interfacial and dipole polarization (Fig. 14a3). Adjusting the WS_2_ film thickness increased the real and imaginary permittivity to 4–8 and 3–6, respectively (Fig. 14a4), indicating strong dielectric loss from multi‑interface effects and defect‑induced relaxation. The optimized material achieved a RL_min_ of −72.76 dB at 2.86 mm and an EAB of 7.7 GHz (Fig. 14a5). Bai et al. used vacuum impregnation and hydrothermal reaction to grow nanoflower‑like MoS_2_ inside porous Si_3_N_4_ ceramics with interconnected channels (Fig. 14b1) [193]. The low‑temperature process avoided MoS_2_ decomposition. MoS_2_ evolved from isolated nanoflowers (~ 300 nm) into a continuous coating (~ 160 nm) (Fig. 14b2). TEM confirmed Mo‑N bonding and lattice defects (MoS_2_ (002) spacing 0.65 nm) (Fig. 14b3). By tailoring porosity (56%‑63%) and interfacial polarization, the material achieved full X‑band absorption and an RL_min_ of −68.66 dB at 3.06 mm (Fig. 14b4). Zhao et al. constructed a multi‑layer nested core‑shell heterostructure of V_2_O_3_ and MoS_2_ via crystal epitaxial growth and solvothermal sulfidation (Fig. 14c1) [27]. Controlling sulfur source concentration expanded MoS_2_ lattice spacing and introduced interfacial vacancies, optimizing permittivity and impedance matching. As V_2_O_3_‑MoS_2_ layers increased from two to five, a built‑in electric field formed (Fig. 14c2), inducing strong interfacial polarization and charge transfer (Fig. 14c5). Vacancy defects reduced the band gap and enhanced dielectric loss (Fig. 14c3). The five‑layer microspheres achieved an RL_min_ of –69.65 dB at 3 mm and an ultra‑wide EAB of 7.68 GHz (Fig. 14c4). Luo et al. synthesized a ZnO/MoS_2_ composite by growing MoS_2_ spheres on hollow porous ZnO microspheres (Fig. 14d1) [194]. The composite has a spherical morphology (~ 3.5 µm diameter) with abundant heterointerfaces (Fig. 14d2‑d3). Adjusting MoS_2_ content tuned the permittivity (Fig. 14d4). The sample with 30 wt% MoS_2_ showed optimal impedance matching, achieving a RL_min_ of –35.8 dB at 11.84 GHz (2.5 mm) and an EAB of 10.24 GHz (7.76‑18 GHz) (Fig. 14d5). Performance arises from multiple reflections in hollow structures, interfacial polarization from porous architecture, and conduction loss from MoS_2_. Zhang et al. developed a WS_2_/NiO heterostructure via hydrothermal method [195]. NiO nanoparticles were uniformly loaded on few‑layer WS₂ nanosheets, creating abundant interfaces (Fig. 14e1). The composite benefits from optimized impedance matching and synergy between dielectric loss and magnetic loss. It achieved a RL_min_ of –53.31 dB at 4.30 mm and an ultra‑wide EAB of 13.46 GHz (4.54–18 GHz) (Fig. 14e2). Optimizing NiO content provides synergistic enhancement of dielectric polarization and eddy current loss.

Composites combining low-conductivity dielectric materials like SiC and Si_3_N_4_ with TMDs exhibit exceptional impedance matching characteristics as their core advantage. The minimal conductive loss in these materials effectively prevents surface reflection of EMW, allowing deeper wave penetration. Their absorption mechanism primarily relies on strong polarization relaxation: the heterointerfaces between TMDs and dielectric matrices generate significant interfacial polarization, while inherent lattice defects and covalent characteristics in dielectrics like SiC contribute substantial dipole polarization. The phase transition properties of materials such as V_2_O_3_ can further introduce temperature-responsive smart EMA behavior. This combination achieves an efficient absorption mechanism dominated by polarization loss while maintaining excellent impedance matching. Future research should focus on enhancing multiple scattering and polarization effects through microstructure design, particularly via core–shell structures or 3D porous networks. Precisely engineering defects in dielectric materials to optimize polarization capability represents a key scientific challenge. The fundamental obstacle remains balancing low conductivity with sufficient attenuation capacity, as single polarization mechanisms often prove inadequate for strong EMA. Precise impedance matching regulation across broad frequency ranges remains challenging, while interfacial stability and scalable fabrication processes constitute critical bottlenecks for practical implementation. A more comprehensive theoretical framework is still required to elucidate the electromagnetic response mechanisms in these material systems.

### Magnetic Materials/TMDs Composites

Following the previously discussed dielectric composite systems, the integration of TMDs with magnetic materials, including magnetic metals, alloys, and their oxides, opens a new and more synergistic dimension for designing EMA materials. The unique advantage of such composites lies in their successful fusion of the dielectric loss from TMDs with the magnetic loss mechanisms of the magnetic components, enabling a more comprehensive dissipation of electromagnetic field energy. The magnetic constituents not only effectively enhance the attenuation of electromagnetic energy through natural resonance and hysteresis loss but also, owing to their inherently lower permittivity, facilitate the fine-tuning of the composite's overall impedance. This pushes the impedance closer to that of free space, thereby significantly optimizing the impedance matching characteristics. This dielectric-magnetic synergy allows the material to achieve broader bandwidth absorption at smaller thicknesses, demonstrating comprehensive performance that is difficult to attain with single dielectric-type composite materials.

#### Magnetic Metals or Alloys/TMDs Composites

The integration of magnetic metal or alloys with TMDs signifies a strategic shift from sole dielectric loss to dielectric-magnetic synergistic loss. The core advantage of such materials lies in introducing significant magnetic loss mechanisms, such as natural resonance and exchange resonance, which effectively complement the dielectric loss of TMDs. The magnetic particles not only enhance the conversion efficiency of electromagnetic energy but also, through their characteristic dispersion effects, help optimize the impedance matching of the composite, overcoming the poor low-frequency matching typical of pure dielectric systems. This synergistic dual-loss mechanism enables highly efficient absorption across broader frequency bands.

**Fig. 15:**
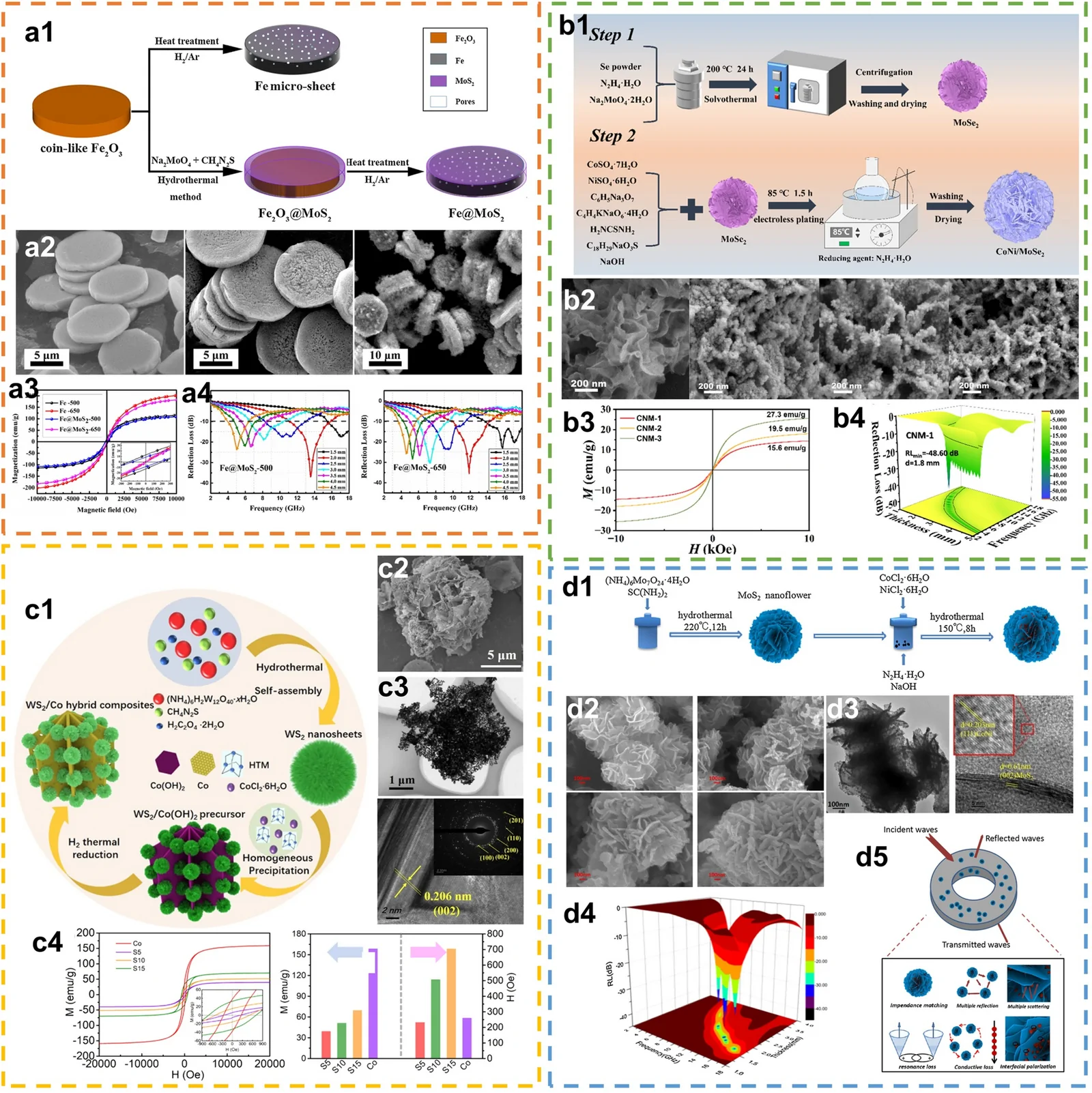
**a1** Schematic illustration of the synthesis process of porous coin-like Fe and Fe@MoS_2_ composites; **a2** SEM images of Fe_2_O_3_ micro-sheet, Fe-500, Fe@MoS_2_-500; **a3** Magnetic hysteresis loops of Fe-500, Fe-650, Fe@MoS_2_-500 and Fe@MoS_2_-650, respectively; **a4** RL curves Fe@MoS_2_-500 and Fe@MoS_2_-650, respectively [196]. Copyright 2018 Elsevier. **b1** Synthetic illustration for the synthetic process of flower-like CoNi/MoSe_2_ composites; **b2** SEM images of MoSe_2_, CNM-1, CNM-2, CNM-3, respectively; **b3** Magnetic hysteresis loops of CNM-x samples; **b4** 3D RL plots of CNM-1 [197]. Copyright 2023 Elsevier. **c1** Synthetic illustration for WS_2_/Co hybrid composites with heterostructures; **c2** SEM and HRTEM images of WS_2_/Co; **c3** TEM images of WS_2_/Co; **c4** Magnetic hysteresis loops and corresponding *M*_*s*_ and *H*_*c*_ of different samples [198]. Copyright 2022 Elsevier. **d1** Schematic diagram of the synthesis procedure for MoS_2_/CoNi composites; **d2** SEM images of prepared MoS_2_/CoNi with different ratio; **d3** TEM and HRTEM images of prepared MoS_2_/CoNi; **d4** RL-Z curves and 3D RL plot of S3 sample; **d5** EMA absorption mechanism in MoS_2_/CoNi composites [199]. Copyright (2021) Springer Nature

Pan et al. fabricated porous coin‑like Fe@MoS_2_ composites via solvothermal synthesis followed by annealing in H_2_/Ar (Fig. 15a1) [196]. MoS_2_ nanosheets uniformly coated porous Fe microflakes, forming a core‑shell structure (Fig. 15a2). The sample annealed at 650 °C exhibited enhanced saturation magnetization (201 emu g^−1^) and soft magnetic behavior (Hc < 12.57 Oe) (Fig. 15a3). By balancing dielectric loss from MoS_2_ with magnetic loss from Fe, impedance matching was optimized. The 500 °C sample achieved an EAB of 4.73 GHz (11.13–15.86 GHz) at 2.0 mm, with a RL_min_ of –37.02 dB, while the 650 °C sample shifted absorption to lower frequencies (Fig. 15a4). The porous flake morphology even surpasses Snoek’s limit, as the hierarchical porous structure introduces additional shape anisotropy and local demagnetizing fields, which delay the natural resonance frequency roll‑off and enhance the high‑frequency magnetic loss capability. This highlights the advantage of magnetic/dielectric synergy. Yu et al. synthesized 3D flower‑like CoNi/MoSe_2_ composites via hydrothermal growth followed by electroless plating (Fig. 15b1) [197]. MoSe_2_ formed a flower‑like scaffold, with CoNi alloys uniformly deposited on its surface (Fig. 15b2). By tuning the Co/Ni molar ratio, the 1:3 sample showed optimal magnetic and EMA properties, with a Ms of 15.6 emu g^−1^ (Fig. 15a3). It delivered a RL_min_ of −48.6 dB at 1.8 mm and an EAB of 3.76 GHz at only 1.4 mm (Fig. 15b4). The work demonstrates that careful control of alloy composition can effectively combine magnetic loss with dielectric loss for improved impedance matching.

Li et al. designed WS_2_/Co hybrid composites via a precursor‑guided strategy, forming flower‑like architectures where WS_2_ nanoclusters anchored on Co nanosheets (Fig. 15c1) [198]. The 3D self‑assembled morphology featured abundant WS_2_/Co interfaces (Fig. 15c2, c3). Magnetic characterization showed strong ferromagnetism: Ms increased from 39.7 to 70.1 emu g^−1^ with Co content, while Hc (233–706 Oe) enhanced magnetic loss (Fig. 15c4). The optimal sample (S10) achieved a RL_min_ of −46.5 dB at only 1.7 mm thickness, highlighting the effectiveness of magnetic metal/TMDs heterostructures for thin absorbers. Hu et al. prepared flower‑like MoS_2_/CoNi composites via a two‑step hydrothermal method [199]. A flower‑like MoS_2_ substrate was first obtained, then CoNi nanoparticles were uniformly decorated on its surface (Fig. 15d1, d2). TEM confirmed intimate contact between CoNi and MoS_2_ (002) planes (Fig. 15d3). The inclusion of CoNi significantly improved impedance matching and introduced magnetic loss. The optimal sample (S3) achieved a RL_min_ of −41.44 dB at 13.53 GHz and an EAB of 5.6 GHz at 2.0 mm (Fig. 15d4). Multiple mechanisms synergize here: flower‑like structure promotes multiple scattering, abundant MoS_2_/CoNi interfaces induce interfacial polarization, and CoNi provides both magnetic loss and enhanced conductivity. This work exemplifies the advantages of electromagnetic synergy in flower‑like architectures.

In summary, magnetic metal/alloy and TMDs composites demonstrate remarkable advantages in broadening the absorption bandwidth and optimizing impedance matching through the construction of a dielectric-magnetic synergistic system. Future research will focus on the precise regulation of interface engineering and exploration of 3D spatial assembly to maximize synergistic effects. However, challenges remain, including the susceptibility of magnetic components to oxidation, the decline of high-frequency permeability, and eddy current effects. Breakthroughs in long-term stability and scalable fabrication processes are still required.

#### Magnetic Metal Oxides/TMDs Composites

**Fig. 16:**
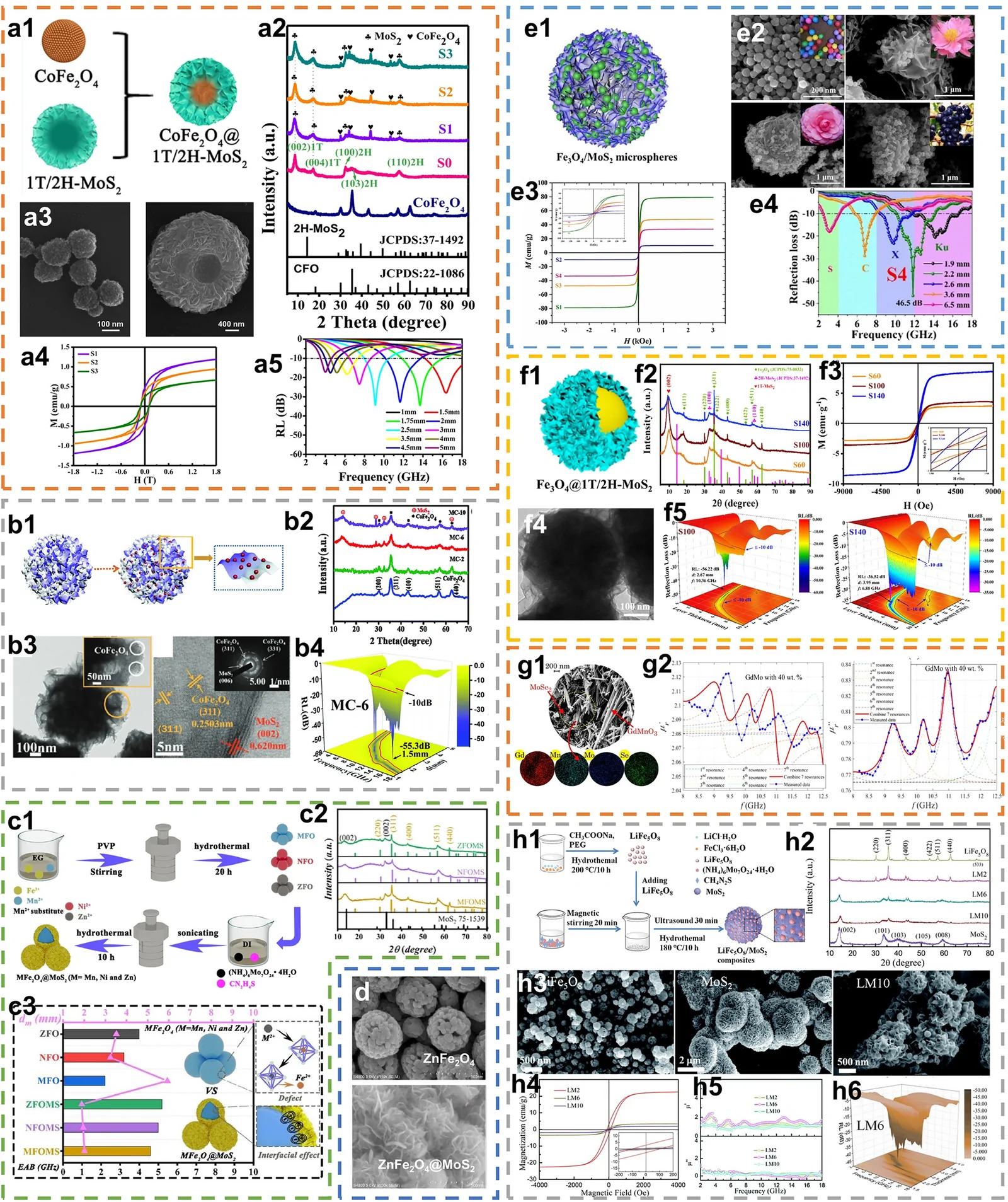
**a1** Synthesis route of CoFe_2_O_4_@1T/2H-MoS_2_ composites; **a2** XRD patterns of CoFe_2_O_4_, pure 1T/2H-MoS_2_ and composites; **a3** SEM images of CoFe_2_O_4_ and1T/2H-MoS_2_ respectively; **a4** Magnetic hysteresis loops of CoFe_2_O_4_@1T/2H-MoS_2_ composites; **a5** RL curves S2 sample[200]. Copyright 2020 American Chemical Society. **b1** Synthetic illustration for the synthesis process for MoS_2_/CoFe_2_O_4_ composites.; **b2** XRD patterns of MoS_2_/CoFe_2_O_4_ composites; **b3** TEM and HRTEM images of MoS_2_/CoFe_2_O_4_; **b4** RL curves of MC-6 [201]. Copyright 2019 Royal Society of Chemistry. **c1** Schematic diagram for the synthesis of core–shell structure MFe_2_O_4_@MoS_2_ (M = Mn, Ni and Zn) flower-like MCNCs; **c2** XRD patterns of MFe_2_O_4_@MoS_2_ samples; **c3** Summary of the EAB and matching thickness of MFe_2_O_4_@MoS_2_ samples [202]. Copyright 2023 Elsevier. **d** SEM images of ZnFe_2_O_4_ and ZnFe_2_O_4_@MoS_2_ [203]. Copyright 2021 Elsevier. **e1** Illustrative diagram of Fe_3_O_4_/MoS_2_ microspheres; **e2** SEM images of Fe_3_O_4_/MoS_2_ microspheres with typical shape; **e3** Magnetic hysteresis loops of Fe_3_O_4_/MoS_2_ composites; **e4** RL curves of S4 sample [204]. Copyright 2020 Elsevier. **f1** Illustrative diagram of Fe_3_O_4_@1T/2H-MoS_2_ microspheres; **f2** XRD patterns and **f3** magnetic hysteresis loops of S60, S100, S140 samples; **f4** TEM images of S100; **f5** 3D RL plots of S100 and S140 [205]. Copyright 2022 Elsevier. **g1** SEM images and elemental mapping of GdMnO_3_-MoSe_2_; **g2** Permeability curves of GdMnO_3_-MoSe_2_ with 40 wt% and their fitting result [206]. Copyright 2023 Elsevier. **h1** Synthetic illustration for the synthesis process of LiFe_5_O_8_/MoS_2_ composites; **h2** XRD patterns of different samples; **h3** SEM images of LiFe_5_O_8_, MoS_2_, LM10 samples, respectively; **h4** Magnetic hysteresis loops of LMx samples; **h5** Permeability curves of LMx samples; **h6** 3D RL plot of LM6 [207]. Copyright 2020 Royal Society of Chemistry

Compared to magnetic metal particles, composites of magnetic metal oxides (such as ferrites) with TMDs demonstrate superior overall performance. These materials retain magnetic loss capabilities while possessing higher resistivity, which effectively suppresses eddy current effects and benefits high-frequency applications. The heterointerfaces formed with TMDs not only enhance interfacial polarization but also optimize charge transport behavior through energy band regulation, achieving profound synergy between dielectric and magnetic losses. Additionally, they offer greater advantages in terms of environmental stability and impedance matching compatibility. Wang et al. fabricated a 3D nest‑like core‑shell CoFe_2_O_4_@1T/2H‑MoS_2_ composite (Fig. 16a1) [200]. SEM showed 150 nm CoFe_2_O_4_ nanospheres embedded in 2‑3 μm MoS_2_ microflowers (Fig. 16a2). XRD confirmed coexistence of both phases (Fig. 16a3). The composite exhibited enhanced coercivity after integration (Fig. 16a4). By tuning MoS_2_ content to optimize impedance matching, a RL_min_ of –68.5 dB at 1.81 mm and an EAB of 4.56 GHz (13.2–17.76 GHz) was achieved (Fig. 16a5, a6). This work highlights the advantage of magnetic/dielectric core‑shell architectures for ultra‑strong absorption. Cui et al. synthesized MoS_2_ decorated with CoFe_2_O_4_ nanoparticles via a two‑step hydrothermal method (Fig. 16b1) [201]. XRD confirmed MoS_2_ (002) and CoFe_2_O_4_ (311) (Fig. 16b2). HRTEM showed lattice spacings of 0.620 nm (MoS_2_) and 0.2503 nm (CoFe_2_O_4_) (Fig. 16b3). With a MoS_2_/CoFe_2_O_4_ mass ratio of 6:1, the composite achieved a RL_min_ of –55.3 dB at 16.24 GHz (Ku‑band) and an EAB of 6.26 GHz in X‑band at 1.6 mm (Fig. 16b4). The performance gain comes from improved impedance matching, enhanced interfacial polarization, and magnetic loss from CoFe_2_O_4_. Xiao et al. synthesized MFe_2_O_4_@MoS_2_ (M = Mn, Ni, Zn) flower‑like core‑shell composites (Fig. 16c1) [202]. MFe_2_O_4_ nanoparticles were prepared first, then MoS_2_ nanosheets grown in situ. Larger M^2+^ ionic radii promoted oxygen vacancies, enhancing dielectric loss. The MFe_2_O_4_@MoS_2_ achieved a RL_min_ of −50.39 dB at 9.4 GHz, while the ZnFe_2_O_4_@MoS_2_ gave an EAB of 5.2 GHz (Fig. 16c3). This strategy is further underscored by the work of Wang et al. [203], who reported a 3D flower-like ZnFe_2_O_4_@MoS_2_ core–shell composite, as shown in Fig. 16d, that exhibited a remarkable RL_min_ of −61.8 dB at 9.5 GHz and a broad EAB of 5.8 GHz, covering frequencies from 7.2 to 13 GHz.

Liu et al. fabricated Fe_3_O_4_/MoS_2_ composites by hydrothermal method [204]. Varying the Fe_3_O_4_/MoS_2_ molar ratio from 1:12.5 to 6:12.5 produced distinct morphologies (Fig. 16e1, e2). XRD confirmed pure phases (Fig. 16e3). The optimal sample (S3) had a saturation magnetization of 17.9 emu g^−1^ and low coercivity of 16.8 Oe (Fig. 16e4). It achieved an ultra‑strong RLmin of –64.0 dB at 1.7 mm and an ultra‑wide EAB of 6.1 GHz at 2.0 mm, covering full C and X bands (Fig. 16e5). This exceptional performance is attributed to optimized impedance matching and a synergistic effect between dielectric and magnetic losses. Similarly, Wu et al. prepared core‑shell Fe_3_O_4_@1T/2H‑MoS_2_ nanocomposites via a straightforward hydrothermal method (Fig. 16f1) [205]. XRD and XPS confirmed the integration of both phases (Fig. 16f2). Magnetic saturation ranged from 2.89 to 8.59 emu g^−1^ (Fig. 16f3). TEM confirmed the core‑shell configuration and flower‑like morphology (Fig. 16f4). The composite delivered a RL_min_ of −56.22 dB at 10.36 GHz (2.67 mm) and an EAB of 4.76 GHz at 1.89 mm (Fig. 16f5). The core‑shell architecture provides well‑matched impedance and multiple dissipation mechanisms. Wang et al. [206] conducted a systematic investigation into the EMA properties of GdMnO_3_-MoSe_2_ composites. GdMnO_3_, a multiferroic material, offers advantages due to its notable magnetostrictive effect, demonstrated by a saturation magnetization of 12.2 emu g^−1^. GdMnO_3_ (multiferroic, Ms = 12.2 emu g^−1^) exhibits rod‑like morphology, while MoSe_2_ nanoflowers occupy interparticle spaces, creating abundant heterointerfaces (Fig. 16g1). A seven‑order Lorentz dispersion model accurately described the complex permeability (real part ~ 2.08, tan*δ*_*m*_ ~ 0.38) (Fig. 16g2). With 40 wt% filler, the composite achieved a RL_min_ of –21.85 dB in X‑band at 2 mm thickness, thanks to balanced dielectric and magnetic losses (tan*δ*_*e*_ ~ 0.228). Li et al. [207] synthesized LiFe_5_O_8_/MoS_2_ nanocomposites via a two-step hydrothermal method. Raspberry‑like LiFe_5_O_8_ nanoparticles (50–200 nm) were uniformly distributed on MoS_2_ microflowers (Fig. 16h1–h3). XRD confirmed LiFe₅O₈ (311) and MoS_2_ (002) (Fig. 16h2). The composite had a Ms of 3.37 emu g^−1^ (Fig. 16h4) and favorable permeability from 2‑18 GHz (Fig. 16h5). With a LiFe_5_O_8_/MoS_2_ mass ratio of 1:6, a RL_min_ of −63.61 dB and an EAB of 5.2 GHz at 2.179 mm were achieved (Fig. 16h6). This superior performance is attributed to an optimized synergy between dielectric and magnetic losses, coupled with significantly enhanced interfacial polarization.

Composite systems of magnetic oxides and TMDs demonstrate unique value in the field of EMA materials through the deep synergy of dielectric-magnetic synergic loss mechanisms. Compared to magnetic metal particles, these materials effectively suppress eddy current effects due to their intrinsic high resistivity while maintaining significant magnetic loss capabilities, exhibiting superior performance particularly at low frequencies. Their core advantages lie in the strong interfacial polarization induced by heterointerfaces, optimized impedance matching characteristics, and excellent environmental stability, collectively establishing an efficient platform for broadband EMA performance.

Throughout this chapter, the fundamental innovation of compounding magnetic components (including metals, alloys, and oxides) with TMDs lies in transcending the limitation of a single dielectric loss mechanism, successfully constructing a dielectric-magnetic synergistic loss system. This strategy not only significantly enhances the conversion and dissipation efficiency of electromagnetic energy but also, through flexible design of composition and structure, enables precise regulation of the macroscopic electromagnetic parameters (complex permittivity and permeability) of the composite. This allows for the simultaneous optimization of impedance matching and attenuation characteristics across a broad frequency range. Future research should focus on exploring the coupling of novel magnetic semiconductor materials with TMDs and striving to further enhance overall performance through microscopic interface engineering and macroscopic multi-dimensional structural design (e.g., 3D ordered structures). Significant challenges remain: effectively enhancing the magnetic loss capability of magnetic components (especially oxides) at low frequencies, achieving highly uniform dispersion of magnetic units within the composite structure to prevent agglomeration, and developing scalable, cost-effective fabrication processes with controlled structures. In-depth exploration and resolution of these key issues are crucial for advancing such composite materials toward practical engineering applications.

### Multicomponent Composite Strategy

Following the exploration of single component (dielectric or magnetic) composites with TMDs, the multicomponent synergistic composite strategy signifies a significant evolution in the design philosophy of EMA materials. This system aims to construct diverse and complementary electromagnetic loss pathways through the precise architectural integration of dielectric loss units, magnetic loss units, and even conductive components with TMDs. Its core scientific value lies in enabling more independent and precise cooperative regulation of the material's macroscopic electromagnetic parameters, thereby overcoming the inherent compromise between impedance matching and attenuation capability across broader frequency bands [208]. This multi-interface, multi-mechanism synergistic effect not only surpasses the performance limits of single component composites but also provides a highly promising design platform for developing a new generation of high-performance EMA materials characterized by "strong absorption, broad bandwidth, and small thickness."

#### Multicomponent /TMDs Composites Dimensional Design

Compared with the carbon/TMDs composites of different dimensionalities discussed in Sect. 5.1, the dimensionality design of multicomponent/TMDs composites not only retains the respective characteristics of absorbers with different dimensions, but also introduces more designable components. This helps improve interfacial bonding, enhance the uniformity of the composite, and significantly increase the interfacial polarization level of the system.

**Fig. 17:**
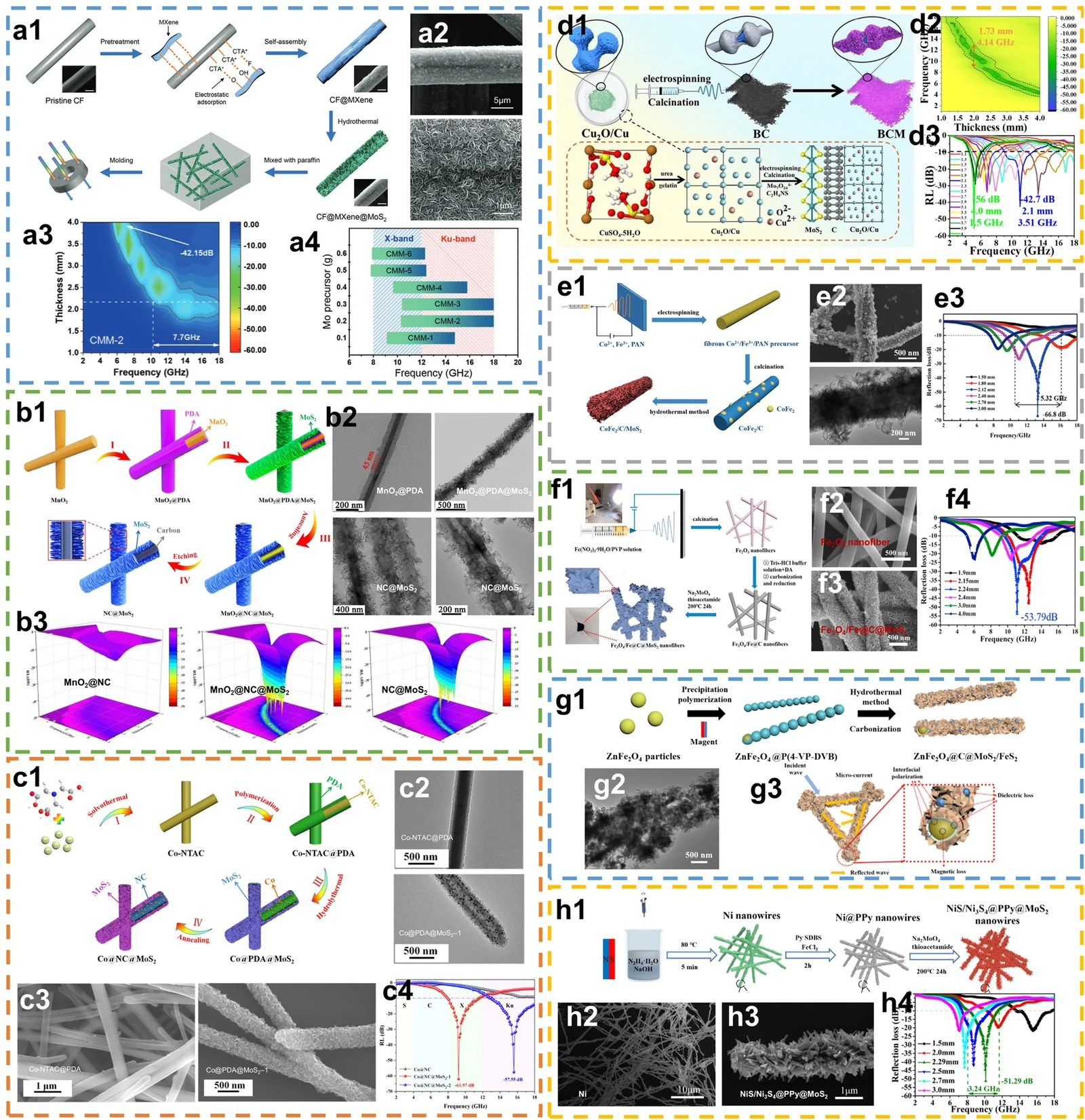
**a1** Schematic diagram of the synthesis process of the CF@MXene@MoS_2_ absorber; **a2** SEM images of CF@MXene@MoS_2_ composite; **a3** XRD patterns of relevant materials; **a4** 2D RL plot of CMM-2 and summary of effective absorption frequency band of CMM-x, respectively [39]. Copyright 2020 Wiley-VCH. **b1** Schematic illustration of the synthesis of NC@MoS_2_ nanotubes; **b2** TEM images of the coating structure of MnO_2_@PDA, MnO_2_@PDA@MoS_2_, NC@MoS_2_, respectively; **b3** 3D RL plots of MnO_2_@PDA, MnO_2_@PDA@MoS_2_, NC@MoS_2_, respectively [209]. Copyright 2021 Elsevier. **c1** The scheme for the fabrication of Co@NC@MoS_2_; **c2** TEM images of the coating structure of Co-NTAC@PDA, Co@PDA@MoS_2_-1; **c3** SEM images of Co-NTAC@PDA, Co@PDA@MoS_2_-1; **c4** RL curves of Co@PDA@MoS_2_-x samples [210]. Copyright 2022 Elsevier. **d1** Schematic illustration of the formation of Cu/Cu_2_O, Cu/Cu_2_O-CF and Cu/Cu_2_O-CF@MoS_2_; **d2** 2D RL plot of BCM-120; **d3** RL curves of BCM-120 [211]. Copyright 2024 Elsevier. **e1** Schematic diagram of the synthesis of CoFe_2_/C@MoS_2_; **e2** SEM and TEM images of CoFe_2_/C@MoS_2_ core–shell structure; **e3** RL curves of CoFe_2_/C@MoS_2_ [212]. Copyright 2021 Royal Society of Chemistry. **f1** Schematic diagram of the synthesis route of hierarchical Fe_3_O_4_/Fe@C@MoS_2_ core–shell nanofibers; SEM image of **f2** Fe_2_O_3_ nanofibers and **f3** Fe_3_O_4_/Fe@C@MoS_2_ nanofibers; **f4** RL curves of Fe_3_O_4_/Fe@C@MoS_2_ [213]. Copyright 2021 Elsevier. **g1** Schematic diagram of synthesis process of ZnFe_2_O_4_@carbon@MoS_2_/FeS_2_; **g2** TEM image of ZnFe_2_O_4_@carbon@MoS_2_/FeS_2_; **g3** Schematic illustration of the possible EMW loss mechanisms for ZnFe_2_O_4_@carbon@MoS_2_/FeS_2_; **g4** RL curves of ZnFe_2_O_4_@carbon@MoS_2_/FeS_2_ [214]. Copyright 2021 Elsevier. **h1** Synthetic diagram for the synthesis route of NiS/Ni_3_S_4_@PPy@MoS_2_ nanowires; SEM images of **h2** Ni nanowires and **h3** NiS/Ni_3_S_4_@PPy@MoS_2_ nanowires; **h4** RL curves of NiS/Ni_3_S_4_@PPy@MoS_2_ at 40 wt% [215]. Copyright 2021 Elsevier

Multicomponent compositing based on different dimensions represents a key strategy for overcoming the performance limitations of single dielectric or magnetic modification. By introducing a third functional component, it is possible to construct more abundant heterogeneous interfaces on the fiber surface, synergistically enhancing interfacial polarization and multiple scattering effects. Furthermore, the precise electromagnetic parameter complementarity between the components enables the cooperative optimization of both impedance matching and attenuation characteristics for the composite. This design philosophy significantly expands the performance regulation dimensions for 1D EMA materials. Wang et al. [39] reported a novel hierarchical core-sheath structured microwave absorber, CF@MXene@MoS_2_ (CMM), successfully fabricated via self-assembly and hydrothermal methods. This architecture employs a 1D CF template, an intermediate Ti_3_C_2_T_*x*_ MXene dielectric layer, and an outer MoS_2_ coating, forming a unique 1D-2D synergistic structure (Fig. 17a1). SEM images revealed that MXene uniformly coats the CF surface, forming a wrinkled sheath, while MoS_2_ nanosheets align vertically to constitute the outer layer (Fig. 17a2). XRD confirmed the presence of both Ti_3_C_2_T_*x*_ and 2H-MoS₂ phases (Fig. 17a3). By varying the MoS_2_ precursor loading from 0.1 to 0.6 g, the material exhibited tunable EMA properties. The optimal performance included a RL_min_ of −61.51 dB at a 3.5 mm thickness and a maximum EAB of 7.6 GHz, covering the entire Ku-band (Fig. 17a4). The material's advantages stem from multiple factors: the MXene sheath enhances conductive loss, the MoS_2_ outer layer improves impedance matching, and MoS_2_ concurrently protects the MXene from oxidation, collectively endowing the CMM composite with highly efficient absorption and excellent stability.

Zhang and colleagues reported the synthesis of a novel core–shell structured NC@MoS_2_ nanotube and its exceptional EMA performance [209]. The synthesis employed MnO_2_ nanowires as a sacrificial template. A sequential process involving polydopamine coating and subsequent surface growth of MoS_2_ nanosheets was conducted, followed by carbonization and acid etching to yield the final hollow tubular structure (Fig. 17b1). TEM characterization revealed a distinct core–shell morphology, wherein MoS_2_ nanosheets were uniformly distributed on the surface of the N-doped carbon layer, forming a hierarchical heterostructure (Fig. 17b2). This multicomponent TMDs composite leverages the construction of heterogeneous interfaces, the introduction of dielectric loss, and the formation of a conductive network. These features collectively enable superior impedance matching and a synergistic interplay of multiple loss mechanisms. Consequently, the material achieved a RL_min_ of −52.56 dB at a thickness of 2.4 mm, along with an EAB of 6.0 GHz (Fig. 17b3), demonstrating significant potential for application as a lightweight and high-efficiency microwave absorber. Subsequently, following this line of thinking, they reported the fabrication and properties of a novel, lightweight 1D EMA material, the Co@NC@MoS_2_ core–shell composite [210]. The research team successfully constructed magnetic hierarchical nanotubes through oxidative self-polymerization followed by a one-step hydrothermal method (Fig. 17c1). TEM analysis revealed a well-defined multilayer core–shell architecture, comprising cobalt nanoparticles uniformly dispersed within N-doped CNT and an outer layer of MoS_2_ nanosheets serving as a transition layer (Fig. 17c2). SEM images showed that the MoS_2_ nanosheets uniformly distributed on the nanotube surface, creating a 3D nanoflower-like structure (Fig. 17c3). The electromagnetic parameters could be further optimized by tuning the MoS_2_ loading, which resulted in exceptional EMA performance. At a 15 wt% filler loading, the composite achieved a RL_min_ of −61.97 dB at 9.2 GHz and an EAB of 5.6 GHz (Fig. 17c4).

The multicomponent strategy also introduces new design freedom to tailor the solution processability of the system. By integrating spinnable agents like RGO or PAN, the composite can be engineered for fabrication via electrospinning or wet-spinning, overcoming the intrinsic limitations of TMDs for fiber formation. Zhao et al. reported a 1D multicomponent TMDs composites, designated Cu/Cu_2_O-CF@MoS_2_, fabricated via hydrothermal temperature control combined with electrospinning [211]. The synthesis involved precisely tuning the hydrothermal temperature within a range of 100–130 °C, with 120 °C identified as the optimum condition for forming a uniform, flower-like MoS_2_ coating (Fig. 17d1). The resulting material possesses a unique hollow multilayer bow-tie structure. XRD confirmed the presence of both Cu/ Cu_2_O and 1T/2H mixed-phase MoS_2_. Through deliberate structural design and modulation of the dielectric properties, the composite achieved exceptional EMA performance. It exhibited a RL_min_ of −56 dB at a thickness of 4 mm (Fig. 17d2) and an EAB of 4.14 GHz at a thinner thickness of 1.73 mm (Fig. 17d3). This superior performance originates from the multiple reflection mechanisms enabled by the hollow architecture, the pronounced interfacial polarization at the heterogeneous interfaces, and the synergistic loss contributions from the dual-phase MoS_2_. Liao et al. [212] reported the fabrication and properties of a novel 1D CoFe_2_/C@MoS_2_ composite for EMA. A multi-step methodology involving electrospinning, subsequent high-temperature carbonization, and a final hydrothermal treatment was employed to successfully construct this 1D material with a core–shell architecture (Fig. 17e1). SEM and TEM images revealed fibers with diameters of approximately 500 nm, wherein CoFe_2_ alloy nanoparticles were embedded within the CF and uniformly coated by MoS_2_ nanosheets (Fig. 17e2). The composite exhibited exceptional EMA performance, achieving a RL_min_ of −66.8 dB at 13.28 GHz with a matching thickness of 2.12 mm, concurrently with an EAB of 5.32 GHz spanning from 10.70 to 16.02 GHz (Fig. 17e3). Tong et al. [213] reported hierarchically structured Fe_3_O_4_/Fe@C@MoS_2_ core–shell nanofibers synthesized via electrospinning combined with in situ polymerization and a hydrothermal method (Fig. 17f1). The material was fabricated using a three-step procedure: initially, α-Fe_2_O_3_ nanofibers were obtained via electrospinning (Fig. 17f2). These were subsequently coated with polydopamine and subjected to carbonization/reduction in an H_2_/N_2_ atmosphere to yield the Fe_3_O_4_/Fe@C core–shell structure. Finally, MoS_2_ nanosheets were grown on its surface through a hydrothermal process, forming a flower-like architecture (Fig. 17f3). EMA performance tests demonstrated that with a thickness of 2.24 mm, the material achieved a RL_min_ of −53.79 dB at 11.12 GHz and an EAB of 4.4 GHz, covering frequencies from 9.62 to 14.02 GHz (Fig. 17f4). This excellent performance is attributed to the synergistic effect of multiple loss mechanisms.

Liao et al. [214] constructed a 1D ZnFe_2_O_4_@carbon@MoS_2_/FeS_2_ multicomponent composite through a three-step method involving hydrothermal synthesis, magnetic field-induced polymerization, and high-temperature carbonization (Fig. 17g1). The material's advantages stem from its unique structural features: the 1D chain-like architecture prolongs the EMW transmission path, the heterogeneous MoS_2_/FeS_2_ interfaces enhance polarization loss, and the intermediate carbon layer optimizes impedance matching. SEM revealed that the microsphere chains are coated with MoS_2_ nanosheets and embedded with FeS_2_ particles, forming a hierarchical structure (Fig. 17g2). The absorption mechanism integrates magnetic loss from ZnFe_2_O_4_, dielectric loss from the carbon and MoS_2_ components, and interfacial polarization at the MoS_2_/FeS_2_ junctions (Fig. 17g3). Consequently, the composite achieves strong absorption of −52.5 dB and a broad EAB of 4.98 GHz at a thickness of 2.23 mm (Fig. 17g4), demonstrating a dual optimization of component synergy and structural design. Huang et al. [215] reported coral-like core–shell structured NiS/Ni_3_S_4_@PPy@MoS_2_ nanowires synthesized via a three-step method (Fig. 17h1). This 1D multicomponent TMD system was constructed using a Ni nanowire template (Fig. 17h2), followed by polypyrrole coating and the hydrothermal growth of MoS_2_ nanorods. SEM images revealed a unique coral-like surface morphology, which significantly increases the specific surface area and provides numerous reflection interfaces (Fig. 17h3). By optimizing the filler content to 50 wt%, the composite achieved its peak EMA performance. It exhibited a RL_min_ of −51.29 dB at 10.1 GHz with a thickness of 2.29 mm, along with an EAB of 3.24 GHz (Fig. 17h4). This excellent performance is attributed to the anisotropic characteristics of the 1D architecture, the synergistic dielectric and magnetic losses from multiple components, and optimized impedance matching.

In summary, the multicomponent strategy provides multidimensional control in designing 1D-TMDs composites. Representative systems such as CF@MXene@MoS_2_ core-sheath structures and other fiber-based composites utilize synergistic multicomponent effects to construct abundant heterogeneous interfaces and multiple loss pathways, achieving precise balance between impedance matching and attenuation characteristics [216]. This approach successfully overcomes the performance limitations of single-component or simple binary composite systems. Future research should focus on atomic level interface engineering and spatial distribution control of components, while resolving multi-phase interface stability and optimizing process versatility remain crucial for practical applications.

**Fig. 18:**
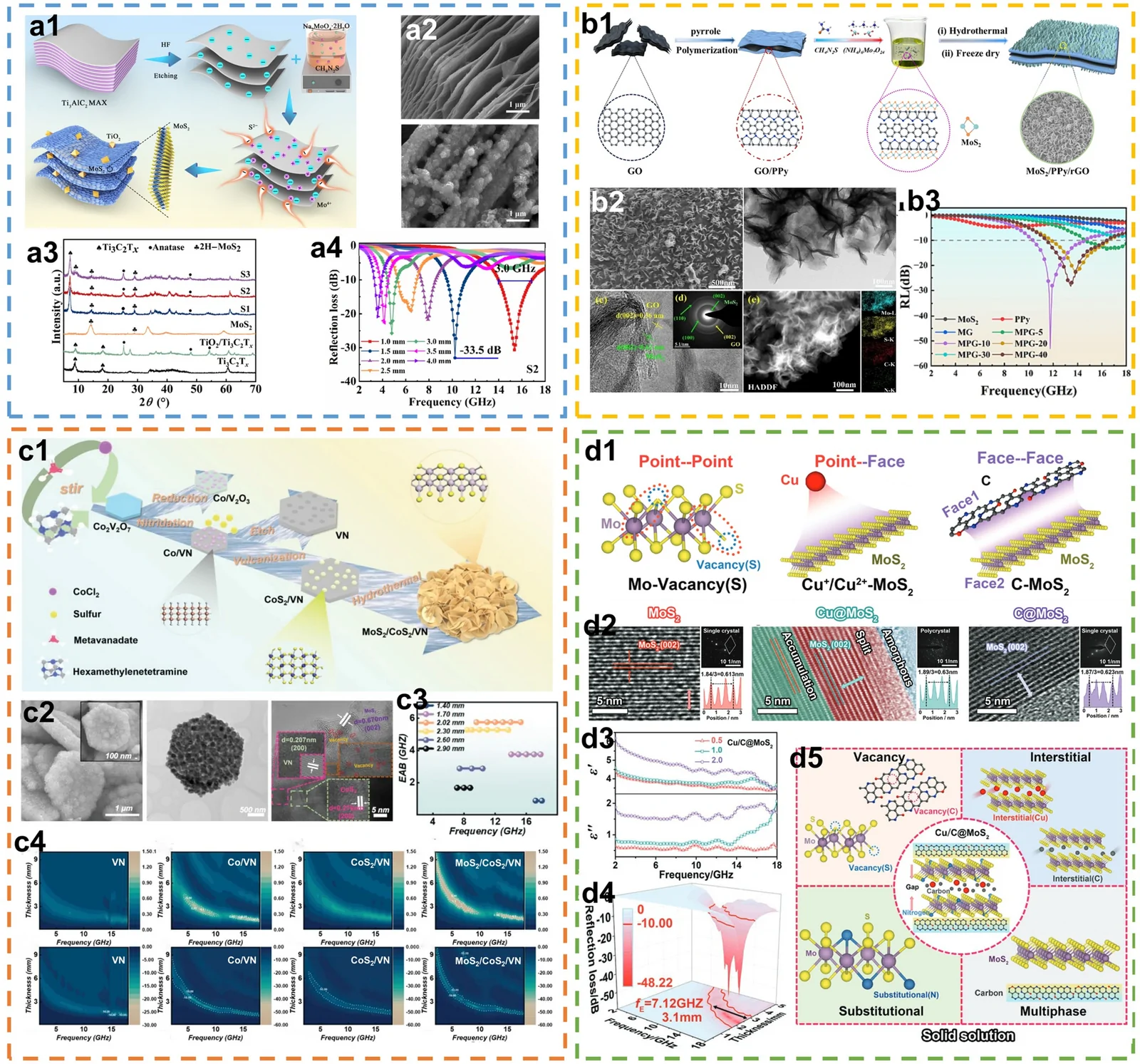
**a1** Schematic diagram of the fabrication process of MoS_2_/TiO_2_/Ti_3_C_2_T_*x*_ composites; **a2** SEM images of Ti_3_C_2_T_*x*_ and MoS_2_/TiO_2_/Ti_3_C_2_T_*x*_ composites, respectively; **a3** XRD patterns of relevant materials; **a4** RL curves of S2 sample [218]. Copyright 2021 Springer Nature. **b1** Schematic illustration of the fabrication process of MoS_2_/PPy/GO (MPG) composites; **b2** SEM, TEM and EDS mapping of MPG-10, respectively; **b3** RL curves of different samples at 2.07 mm [219]. Copyright 2024 Elsevier. **c1** Schematic diagram of the preparation of MoS_2_/CoS_2_/VN composites; **c2** SEM and TEM images of the coating structure of MoS_2_/CoS_2_/VN composites; **c3** Summary of EAB of MoS_2_/CoS_2_/VN at different thicknesses; **c4** Normalized input impendence (up) and 2D RL plots (down) of VN, Co/VN, CoS_2_/VN, MoS_2_/CoS_2_/VN, respectively [220]. Copyright 2023 Wiley-VCH. **d1** Schematic illustration structures of polarization centers in MoS_2_, Cu@MoS_2_, and C@MoS_2_; **d2** HRTEM, SAED patterns of MoS_2_, Cu@MoS_2_, and C@MoS_2_ respectively; **d3** Permittivity curves of Cu/C@MoS_2_ samples; **d4** 3D RL plots of Cu/C@MoS_2-_2.0 sample; **d5** Schematic illustration of vacancy, interstitial, substitutional, and multiphase structure in MoS_2_ composites [40]. Copyright 2022 Wiley-VCH

Distinct from the axial confinement in 1D architectures, 2D multicomponent TMDs composites leverage their planar extensibility to construct denser face-to-face heterointerfaces [217]. This configuration not only significantly enhances interfacial polarization and charge transfer but also enables precise dielectric property regulation through inter-component band engineering. The characteristic 2D electron confinement effect, synergizing with multiple interfaces, provides unique advantages for optimizing electromagnetic parameters and achieving broad band strong absorption. Du et al. [218] reported a hierarchically structured MoS_2_/TiO_2_/Ti_3_C_2_T_*x*_ composite synthesized via a hydrothermal method. The innovative design features vertically anchored MoS_2_ nanosheets within the Ti_3_C_2_T_*x*_ interlayers, accompanied by the in situ generation of TiO_2_ nanoparticles (Fig. 18a1). SEM images revealed that MoS_2_ nanosheets grow uniformly on both the surface and within the interlamellar spaces of Ti_3_C_2_T_*x*_, forming a core–shell-like architecture (Fig. 18a2). XRD analysis confirmed that the incorporation of MoS_2_ inhibits the phase transformation of Ti_3_C_2_T_*x*_ into TiO_2_ while simultaneously expanding the interlayer spacing (Fig. 18a3). Raman spectroscopy further disclosed the characteristic vibration modes of MoS_2_ and a defect-induced shift in Ti–O bonds within the composite. This material demonstrates exceptional EMA performance, achieving a RL_min_ of −33.5 dB at 10.24 GHz and an EAB of 3.1 GHz at an ultralow thickness of merely 1.0 mm (Fig. 18a4). These results signify the realization of lightweight and broad-frequency EMA characteristics. Liu et al. [219] reported a multilayered MoS_2_/PPy/RGO (MPG) composite synthesized via a hydrothermal method, which features a unique hierarchical structure with ear-like MoS_2_ nanosheet arrays. During synthesis, the modification of RGO with polypyrrole effectively prevents graphene restacking and simultaneously provides active substrates for the vertical growth of MoS_2_ nanosheets, resulting in abundant nano-interfaces (Fig. 18b1). Material characterization confirmed the presence of 1T/2H mixed-phase MoS_2_ in the composite, while TEM verified homogeneous elemental distribution and distinct lattice fringes (Fig. 18b2). For EMA optimization, the polypyrrole content was precisely tuned to achieve optimal impedance matching and synergistic multi-loss mechanisms. Consequently, the MPG-10 sample demonstrated a RL_min_ of −53.5 dB at a thickness of 2.07 mm, along with an EAB of 5.36 GHz at a thinner thickness of 1.82 mm (Fig. 18b3).

Zhou et al. [220] reported the synthesis and performance optimization of a novel MoS_2_/CoS_2_/VN multilayer heterostructure for EMA. A composite featuring triple heterointerfaces was prepared via a straightforward hydrothermal stirring method, achieving synergistic effects among its components (Fig. 18c1). The material exhibits several distinctive characteristics. Regarding the synthesis protocol, a stepwise method was employed to prepare a Co_2_V_2_O_7_ precursor, which was subsequently converted to Co/VN through ammonification, followed by sulfidation and self-assembly to form the final architecture (Fig. 18c2). Structurally, HRTEM revealed well-defined lattice fringes corresponding to the VN (200), Co (111), and CoS_2_ (200) planes, while the MoS_2_ nanosheets exhibited an interlayer spacing of 0.67 nm. In terms of performance optimization, the synergistic interplay between interfacial polarization and defect-induced dipole polarization enabled a RL_min_ of −50.48 dB at a thickness of 2.02 mm and an EAB of 5.76 GHz, concurrently imparting excellent corrosion resistance (Fig. 18c3, c4). This multicomponent design strategy based on transition metal nitrides provides a new perspective for developing advanced EMA materials.

Gao et al. [40] constructed a MoS_2_-based multiphase solid solution, designated Cu/C@MoS_2_, via a metal–organic synergistic strategy. This approach simultaneously regulates multiple structural components to achieve a cooperative polarization loss mechanism, including sulfur vacancies for point-to-point modulation, copper intercalation for point-to-plane interaction, nitrogen substitution for additional point-to-point control, and carbon heterointerfaces for plane-to-plane engineering (Fig. 18d1). HRTEM revealed variations in interplanar spacing, with measured values of 0.613 nm for MoS_2_, 0.630 nm for Cu@MoS_2_, and 0.623 nm for C@MoS_2_, thereby confirming successful structural modulation (Fig. 18d2). Notably, Cu intercalation significantly enhanced the permittivity, increasing the imaginary part by a factor of 4.16, which is attributed to improved charge carrier mobility. Concurrently, the incorporated carbon phase enlarged the interlayer spacing, thereby optimizing the multiple polarization response (Fig. 18d3). The resulting Cu/C@MoS_2_ composite achieved a RL_min_ of −48.22 dB at a thickness of 2.5 mm and an EAB of 7.12 GHz (Fig. 18d4). Its energy attenuation efficiency was enhanced by 222–267% compared to any single component. This work establishes a new model for enhancing EMA performance in TMDs materials through multiscale polarization coupling.

#### Multicomponent /TMDs Composites Core–Shell Engineering

**Fig. 19:**
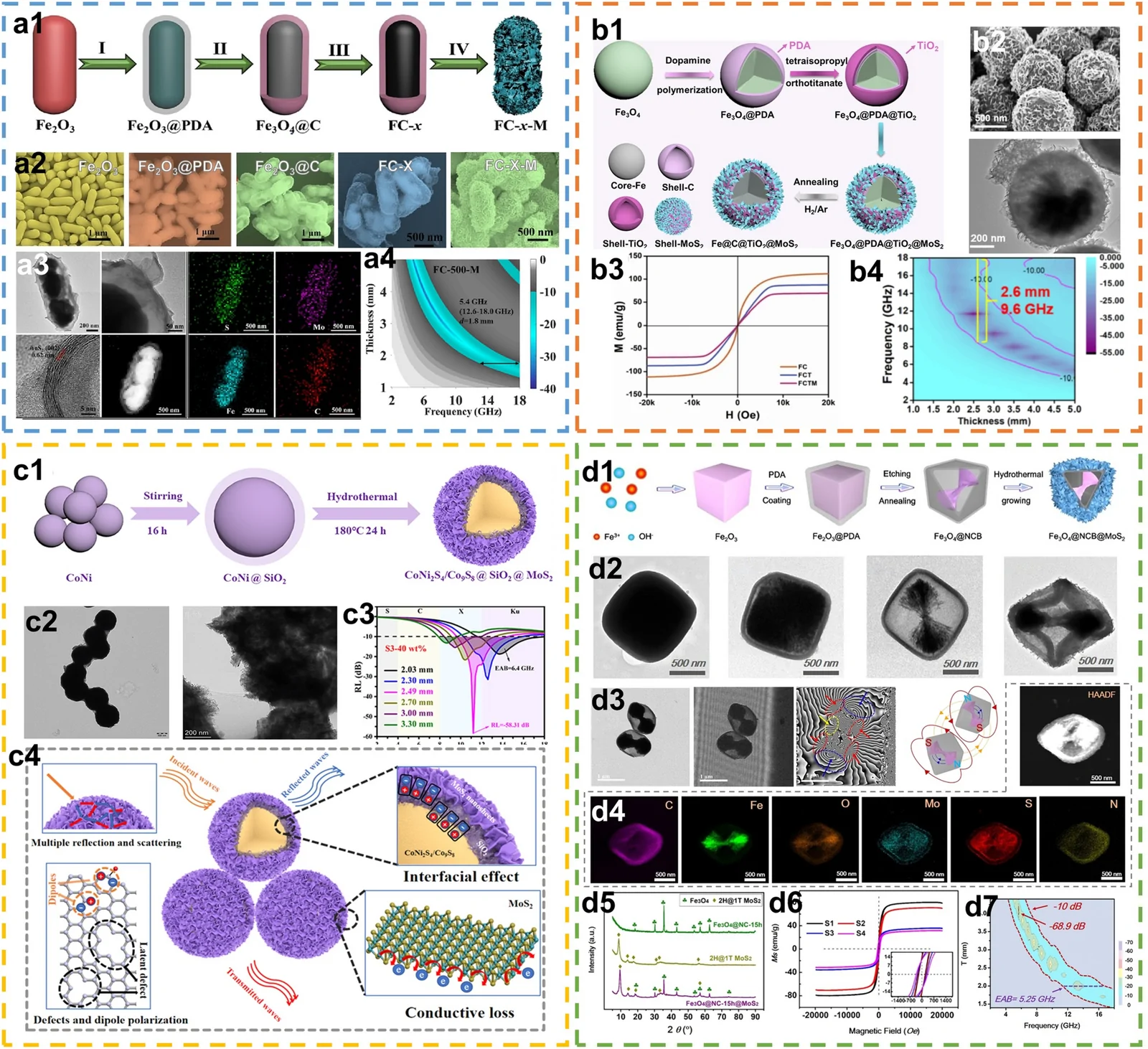
**a1** Schematic diagram of the synthesis process of FC-x-M composites; **a2** SEM images of corresponding materials; **a3** TEM, HAADF images and EDS mappings of FC-500-M; **a4** 2D RL plot of FC-500-M [223]. Copyright 2023 Elsevier. **b1** Schematic diagram for the preparation of multi-shell Fe@C@TiO_2_@MoS_2_ microspheres; **b2** SEM and TEM images of FCTM composite; **b3** Magnetic hysteresis loops of FC, FCT, FCTM composites; **b4** 2D RL plot of FCTM composites [224]. Copyright 2022 Elsevier. **c1** Schematic diagram of coating process for the core@shell structured CoNi_2_S_4_/Co_9_S_8_@SiO_2_@MoS_2_ flower-like MCNCs; **c2** TEM images of CoNi@SiO_2_ and MCNCs; **c3** RL curves of S3; **c4** EMW loss mechanisms in CoNi_2_S_4_/Co_9_S_8_@SiO_2_@MoS_2_ sample [225]. Copyright 2023 Elsevier. **d1** Schematic diagram of the synthesis process for dumbbell-like Fe_3_O_4_@NC@MoS_2_; **d2** TEM images of Fe_3_O_4_@NC in different processing stages; **d3** TEM, off-axis electron hologram and reconstructed magnetic field distribution in S3 sample; **d4** HAADF and EDS mapping of S4 sample; **d5** XRD patterns of relevant samples; **d6** Magnetic hysteresis loops of S1-S4 samples; **d7** 2D RL plot of S4 at 50 wt% [226]. Copyright 2021 American Chemical Society

Core shell engineering is an efficient design strategy for constructing heterointerfaces in EMA materials. This strategy not only achieves complementary performance through the differences in intrinsic electromagnetic properties between different components, but also significantly enhances the absorption performance by forming abundant heterointerfaces among the components. The construction of multicomponent core–shell architectures based on TMDs exceptional coating properties demonstrates an advanced interfacial engineering strategy in EMA absorption material design [221]. These precisely tailored core–shell configurations enable synergistic optimization of graded electromagnetic parameter distribution and impedance matching characteristics [222]. Liu et al. [223] constructed a multicomponent core–shell material, Fe/Fe_3_O_4_@C@MoS_2_, through a four-step methodology (Fig. 19a1). The process began with the preparation of ellipsoidal Fe_2_O_3_ precursors, which had an average length of 1.2 μm and a diameter of 0.4 μm. These precursors were uniformly coated with polydopamine to form an initial core–shell structure. Subsequent carbothermal reduction under a nitrogen atmosphere yielded Fe_3_O_4_@C microcapsules, with SEM images revealing their rough surfaces and internal cavities. A further hydrogen reduction step allowed for precise control over the mass ratio of Fe to Fe_3_O_4_ in the core (0–100%), while SEM confirmed the preservation of the intact capsule morphology (Fig. 19a2). In the final step, vertically aligned MoS_2_ nanosheets were grown via a hydrothermal reaction. TEM analysis showed a 60 nm carbon shell overlaid with MoS_2_ exhibiting a lattice spacing of 0.62 nm, indicative of a 1T/2H mixed phase. EDS elemental mapping confirmed the spatial distribution of the Fe core and the C/S/Mo shell (Fig. 19a3). This design achieves a performance breakthrough via dual optimization pathways: an increased Fe content in the core enhances magnetic loss, yielding a saturation magnetization of 67.3 emu g^−1^ and enabling the material to surpass the Snoek limit, while the introduction of MoS_2_ nanosheets contributes multiple interfacial and dipole polarization effects. Additionally, the porous structure promotes multiple reflections of EMW. The optimal sample, designated FC-500-M, achieved a wide EAB of 5.4 GHz and a RL_min_ of −36.1 dB at a thin thickness of 1.8 mm (Fig. 19a4), successfully demonstrating the advantages of precise component control coupled with multiscale structural design synergy. Huang et al. [224] developed an innovative synthesis for a multicomponent core–shell structured Fe@C@TiO_2_@MoS_2_ EMW absorber (Fig. 19b1). An in situ reduction process was employed to construct a four-layer heterostructure, wherein the magnetic Fe core interfaces sequentially with a dielectric carbon layer, TiO_2_ nanocrystals, and an outer layer of MoS_2_ nanosheets, creating multiple heterogeneous interfaces (Fig. 19b2). Magnetic characterization revealed a high saturation magnetization of 69.9 emu g^−1^ (Fig. 19b3). This architecture, through deliberate interfacial compatibility engineering, enables synergistic polarization at the Fe–C, C–TiO_2_, and TiO_2_–MoS_2_ junctions. Combined with the conduction loss from the carbon interlayer and magnetic coupling effects, the composite achieved a RL_min_ of −54.2 dB at a thickness of 2.5 mm, along with an EAB of 9.6 GHz, which covers the entirety of the X and Ku bands (Fig. 19b4). The multi-shell design strategy presented herein provides a new paradigm for developing high-performance EMA materials.

Yang et al. [225] reported a core–shell structured CoNi_2_S_4_/Co_9_S_8_@SiO_2_@MoS_2_ flower-like multicomponent nanocomposite fabricated through sequential hydrothermal synthesis, a Stöber process, and additional hydrothermal treatment. The construction of abundant heterogeneous interfaces within this material significantly enhances the interfacial polarization effect (Fig. 19c1). Its unique core–shell configuration, which comprises a magnetic CoNi alloy core, an insulating SiO_2_ interlayer, and a dielectric MoS_2_ outer shell, forms a 3D hierarchical morphology that enables the synergistic optimization of impedance matching and loss capability (Fig. 19c2). In terms of EMA performance, the composite exhibits a RL_min_ of −58.31 dB and an EAB of 6.40 GHz (Fig. 19c3). This exceptional performance originates from multiple concurrent loss mechanisms: interface polarization dominated dielectric loss, inherent magnetic loss, and multiple scattering effects induced by the core–shell architecture (Fig. 19c4). Ning et al. [226] reported the synthesis and EMA properties of a dumbbell-shaped Fe_3_O_4_@N-doped carbon@2H/1T-MoS_2_ core–shell material. A composite with multiple interfaces was constructed through controlled acid etching and wet chemical methods (Fig. 19d1). The process began with the acid etching of Fe_3_O_4_ to modulate its morphology from cubic to a dumbbell shape, followed by carbon coating and subsequent MoS_2_ growth to form a hierarchical architecture (Fig. 19d2). Electron holography demonstrated that the dumbbell-like structure enhanced the magnetic coupling effect (Fig. 19d3), while EDS mapping confirmed the multicomponent distribution of Fe_3_O_4_, N-doped carbon, and MoS_2_ (Fig. 19d4). XRD patterns indicated the presence of a 2H/1T mixed phase MoS_2_ and Fe_3_O_4_ (Fig. 19d5). Magnetic measurements revealed that the saturation magnetization decreased from 80.06 to 31.5 emu g^−1^ with prolonged etching time (Fig. 19d6). The material exhibited exceptional EMA performance, achieving a RL_min_ of −68.9 dB at 5.8 GHz and an EAB of 5.2 GHz (Fig. 19d7). This outstanding performance is attributed to optimized impedance matching, multi-interfacial polarization, and enhanced dielectric loss resulting from charge imbalance at the 2H/1T phase boundaries.

**Fig. 20:**
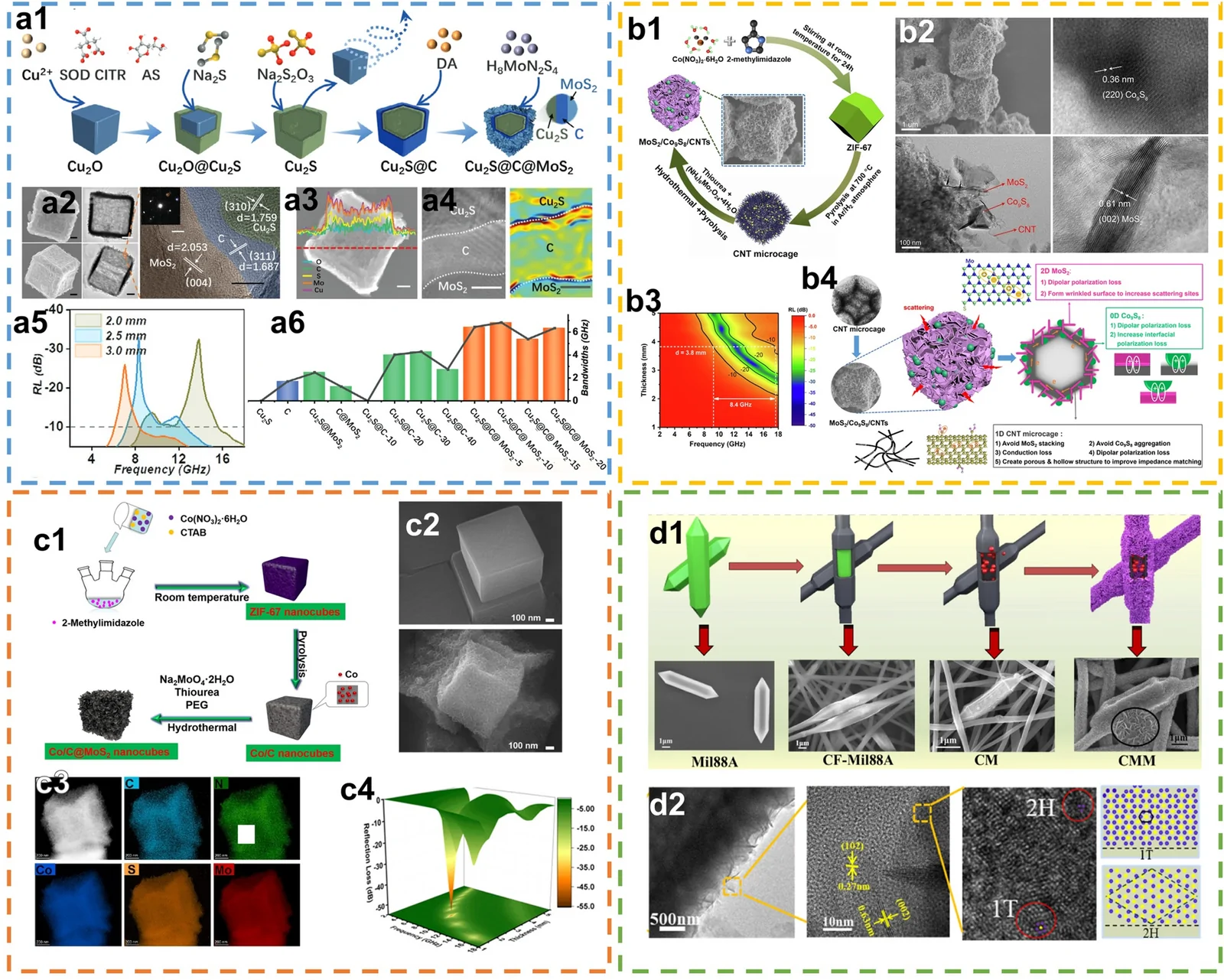
**a1** Schematic diagram of the synthesis process of Cu_2_S@C@MoS_2_ composites; **a2** SEM, TEM and HRTEM images of Cu_2_S@C@MoS_2_ composites; **a3** EDS line scanning of Cu_2_S@C@MoS_2_ cube; **a4** holograms and charge density maps at interface area; **a5** RL curves of Cu_2_S@C@MoS_2_-15; **a6** Summary of EAB of relevant materials [227]. Copyright 2023 Wiley-VCH. **b1** Schematic of the synthesis of Co_9_S_8_/CNTs/MoS_2_; **b2** SEM, TEM images of Co_9_S_8_/CNTs/MoS_2_; **b3** 2D RL plot of different Co_9_S_8_/CNTs/MoS_2_; **b4** schematic diagram of EMA mechanism [228]. Copyright 2021 Elsevier. **c1** Schematic illustration of the synthesis process of Co/C@MoS_2_ nanocubes; **c2** SEM images of ZIF-67 and Co/C@MoS_2_ coating structure of MoS_2_/CoS_2_/VN composites; **c3** HAADF image and EDS mapping of Co/C@MoS_2_; **c4** 3D RL plot of Co/C@MoS_2_ [229]. Copyright 2022 Elsevier. **d1** Schematic illustration of coating structure and corresponding SEM images; **d2** TEM and HRTEM of 2H and 1T-MoS_2_ [230]. Copyright 2023 Elsevier

By extending the multicomponent core shell design strategy, the synergistic system of MOF derived materials and TMDs elevates interface engineering to the molecular level. During the pyrolysis of MOF precursors, metal elements lead to the formation of metal or metal oxide nanoparticles encapsulated within a carbon matrix, resulting in a more uniform and well dispersed core shell structure. This process fully embodies the concept of "precision synthesis" advocated by the 2025 Nobel Prize in Chemistry, as precisely controlling the pyrolysis of MOF precursors allows the construction of hierarchical porous structures with atomically dispersed active sites. These composites combine structural programmability with functional tunability, offering new perspectives for developing next generation high performance EMA materials. Zhang et al. [227] reported the construction of multi-shelled Cu_2_S@C@MoS_2_ nanocubes through an in situ stepwise assembly approach (Fig. 20a1). This process involved the sequential deposition of Cu_2_S, carbon, and MoS_2_ layers onto a 20 nm thick hollow cubic framework. A key feature of the synthesis is the insertion of an intermediate carbon layer, which isolates Cu_2_S from MoS_2_, thereby suppressing the formation of disordered heterojunctions and enabling ordered interfacial charge reconstruction. TEM images confirmed the distinct multi-shelled architecture and the well-defined Cu_2_S@C and C@MoS_2_ heterointerfaces (Fig. 20a2). Furthermore, line-scanning analysis via EDS verified the gradient distribution of constituent elements (Fig. 20a3). By precisely tuning the thickness of the intermediate carbon layer and the MoS_2_ content, the interfacial polarization was optimized. Consequently, the material exhibited an ultra-wide EAB of 7.03 GHz at a minimal thickness of 2.0 mm (Fig. 20a5). The breakthrough in broadband EMW attenuation was elucidated by DFT calculations and electron holography (Fig. 20a4), which revealed a mechanism of directional charge reconstruction at the heterointerfaces and confirmed that the multicomponent synergy operates through a dual-frequency resonance response. Sun et al. [228] reported a MOF derived synthetic strategy for multicomponent TMDs composites and their exceptional EMA performance. The researchers utilized ZIF-67 as a precursor to fabricate CNT micro-cages. Subsequently, MoS_2_ nanosheets and Co_9_S_8_ particles were assembled onto their surfaces via a hydrothermal method, constructing a multidimensional hybrid architecture integrating 0D, 1D, and 2D components (Fig. 20b1). SEM images showed that the product retained the dodecahedral morphology of the MOF precursor, with a wrinkled surface texture formed during the process. TEM images confirmed the presence of distinct lattice fringes with spacings of 0.36 and 0.6 nm, corresponding to the Co_9_S_8_ (220) and MoS_2_ (002) crystal planes, respectively (Fig. 20b2). The composite exhibited outstanding EMA performance at an ultralow filler loading of just 9 wt%, achieving a RL_min_ of −35.4 dB and an EAB of 8.4 GHz (Fig. 20b3). This performance optimization originates from three key mechanisms: a conductive network formed by the CNT micro-cages enhancing conduction loss, multiple polarization relaxations generated by the MoS_2_ nanosheets and Co_9_S_8_ particles, and multiscale interfaces working synergistically to improve impedance matching (Fig. 20b4).

Wang et al. [229] successfully constructed Co/C@MoS_2_ core–shell nanocubes via a two-step pyrolysis-hydrothermal method. The MOF-derived Co/C core, obtained from the pyrolysis of ZIF-67 at 700 °C, provides a conductive network and magnetic components. In contrast, the hydrothermally grown MoS_2_ shell, synthesized at 200 °C, optimizes impedance matching through its semiconducting properties (Fig. 20c1). SEM images confirmed that the cubic morphology remained intact after composite formation, with MoS_2_ nanosheets uniformly coating the surface to form a distinct core–shell interface (Fig. 20c2). EDS mappings verified the homogeneous distribution of Co, N, C, Mo, and S elements (Fig. 20c3). The material exhibited exceptional EMA performance, achieving a RL_min_ of −52.76 dB at 8.88 GHz with a thickness of 2.5 mm, and an EAB of 3.84 GHz at a thinner thickness of 1.5 mm (Fig. 20c4). The performance optimization is attributed to a triple synergistic effect: (1) Dipole and interfacial polarization induced by the MoS_2_ shell. (2) The synergistic interplay of conduction and magnetic losses from the Co/C core (3) optimized impedance matching and enhanced multiple scattering enabled by the hierarchical architecture. Zhao et al. [230] developed a novel EMW absorber based on MOF derivatives compounded with TMDs. The research team constructed a core–shell heterostructured CF-Mil-88A@MoS_2_ composite through a three-step methodology. The process involved the initial hydrothermal synthesis of Mil-88A nanorods, followed by their integration with polyacrylonitrile via electrospinning and subsequent carbonization to yield CM fibers. Finally, a flower-like MoS_2_ outer layer was grown hydrothermally (Fig. 20d1). SEM images revealed that the pristine Mil-88A exhibited a smooth nanorod morphology with dimensions of 500 nm in diameter and 6 μm in length. After carbonization, these transformed into CF embedded with Fe_3_O_4_ particles, which were then coated with MoS_2_ to form a distinct hierarchical architecture. TEM images confirmed the clear core–shell structure, and high-resolution TEM imaging identified lattice fringes of 0.63 and 0.27 nm, corresponding to the (002) and (102) planes of MoS_2_, respectively (Fig. 20d2). This material achieved a RL_min_ of −70 dB at a thickness of 2.5 mm, along with an EAB of 4.68 GHz. The performance enhancement stems from a synergistic interplay of three key factors: (1) the controlled graphitization degree and defect density governed by the carbonization temperature. (2) The increased interfacial polarization afforded by the flower-like MoS_2_ structure, (3) the magnetic loss contribution from the Fe_3_O_4_ nanoparticles. These factors collectively enable excellent impedance matching and activate multiple relaxation loss mechanisms.

The primary advantage originates from the strong polarization relaxation induced at the core–shell interface and the functional complementarity between components: the core governs electromagnetic loss mechanisms, while the TMDs outer shell serves dual roles in impedance matching regulation and interfacial polarization enhancement, while simultaneously maintaining the structural integrity of core components, thereby establishing a multi-level synergistic loss mechanism. Future research will focus on atomic-level interface engineering, dynamic response characteristics, and biomimetic structural design, while precise control of core–shell architecture, long-term stability, and scalable fabrication processes remain critical challenges to be addressed.

#### Multifunctional Multicomponent /TMDs Composites

TMDs are not particularly advantageous for intrinsic multifunctional integration. Therefore, to endow TMDs‑based microwave absorbers with additional properties beyond EMW attenuation, hybridization with functional multicomponents (e.g., carbon networks, polymers, phase‑change materials, or magnetic alloys) and rational structural design are essential. Common added functionalities include thermal management [231] (heat dissipation and insulation), mechanical flexibility [232] and resilience, environmental tolerance [233] (corrosion resistance, flame retardancy, water repellency), and energy storage or conversion (photothermal/electrothermal). These features are driven by practical application needs, and their integration is typically achieved through strategies such as constructing hierarchical conductive networks, embedding phase‑change materials, or designing compressible porous structures. Representative examples are grouped below according to their primary added functionality.

**Fig. 21:**
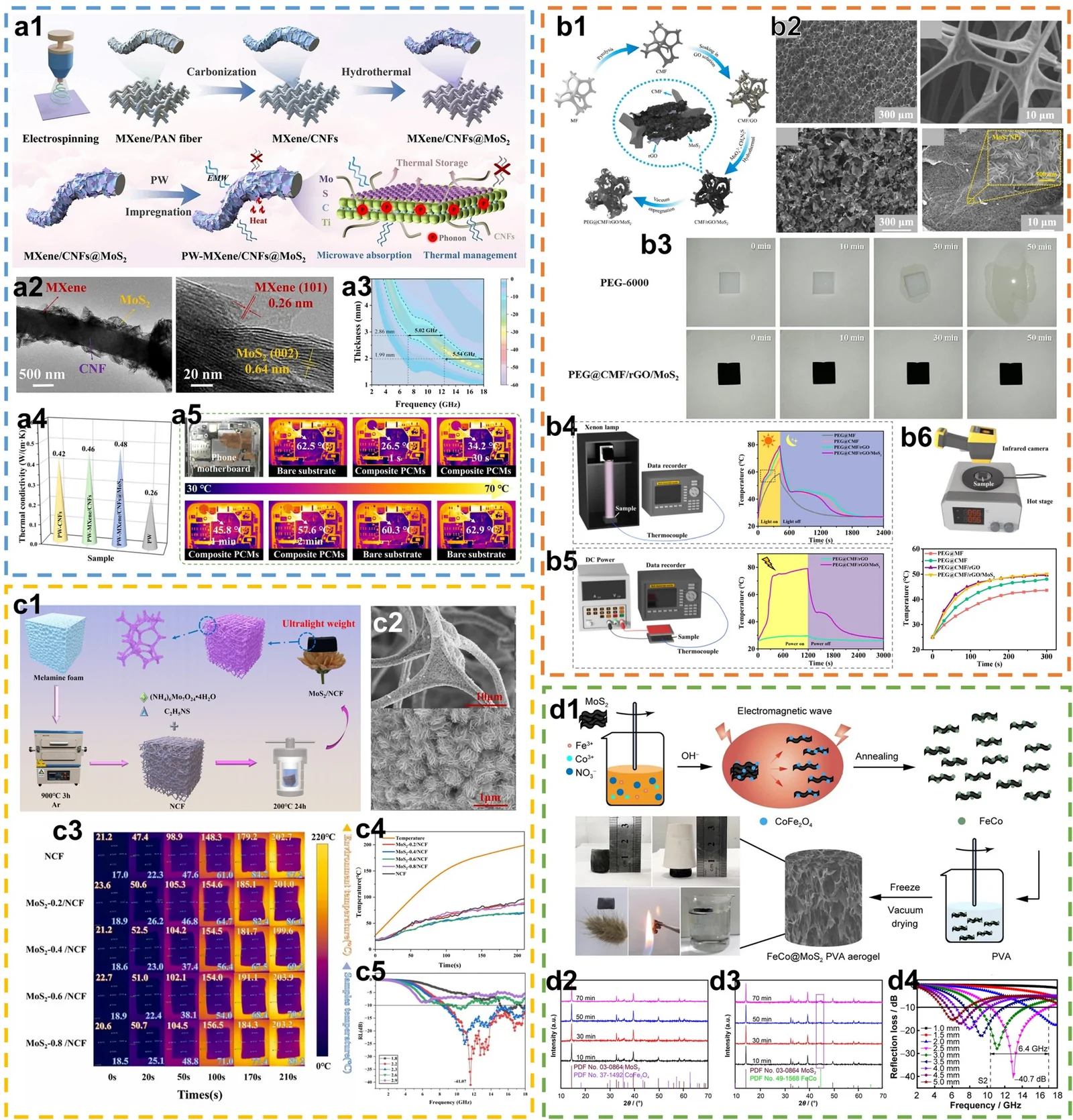
**a1** Schematic diagram of PW-MXene/CNFs@MoS_2_ composite PCMs; **a2** TEM and HRTEM images of MXene/CNFs@MoS_2_; **a3** 2D RL plot of PW-MXene/CNFs; **a4** Thermal conductivity of composite PCMs and PW; **a5** Photograph and infrared images of the motherboard of a working smartphone with and without composite PCMs [234]. Copyright 2025 Springer Nature. **b1** Fabrication route of the CMF/RGO/MoS_2_ network and the corresponding CPCM; **b2** SEM images of CMF (up) and CMF/RGO/MoS_2_ (bottom); **b3** the leakage test result of PEG-6000 and PEG@CMF/RGO/MoS_2_; **b4** Light-to-thermal conversion test equipment and result; **b5** Electric-to-thermal conversion test equipment and result; **b6** Time–temperature curves of the thermal response test equipment and result [235]. Copyright 2023 Elsevier. **c1** Schematic diagram of the fabrication of MoS_2_/NCF composites; **c2** SEM images of MoS_2_-0.4/NCF; **c3** Infrared thermal images and **c4** time–temperature curves of different samples; **c5** RL curves of MoS_2_-0.6/NCF [165]. Copyright 2025 Elsevier. **d1** Schematic diagram illustrating fabrication of FeCo@MoS_2_ PVA aerogel; XRD patterns of **d2** CoFe_2_O_4_@MoS_2_ and **d3** FeCo@MoS_2_; **d4** RL curves of S2 sample [236]. Copyright 2022 Springer Nature

The multicomponent composite strategy effectively overcomes the functional limitations of single TMDs materials, enabling synergistic integration of EMA properties with additional functionalities through precise component design and interface engineering. For the purpose of thermal management and phase‑change energy storage, Feng et al. [234] successfully developed a multifunctional PW-MXene/CNFs@MoS_2_ composite through a multi-interface engineering strategy. The 3D MXene/carbon nanofiber network substrate was first constructed via electrospinning followed by carbonization (Fig. 21a1). MoS_2_ nanosheets were then grown in situ on this substrate through a hydrothermal method, and the final hierarchical composite was obtained via melt impregnation. TEM characterization confirmed the uniform coating of MoS_2_ nanosheets on the fiber surfaces. HRTEM images revealed distinct lattice fringes with spacings of 0.26 and 0.62 nm, corresponding to the MXene (101) and MoS_2_ (002) planes, respectively (Fig. 21a2). In terms of performance, the material exhibits exceptional EMA characteristics, achieving a RL_min_ of −64.1 dB and an effective bandwidth of 4.28 GHz (Fig. 21a3), alongside a significantly enhanced thermal conductivity of 0.48 W m^−1^ K^−1^ (Fig. 21a4). The multifunctional mechanism can be attributed to several key factors. First, the interfacial polarization effect induced by the MXene-CNFs-MoS_2_ multi-level heterostructure. Second, the dipole relaxation promoted by the edge defects of MoS_2_. Third, the efficient phonon transport channels established by the CNFs network. Fourth, the optimized EMA through graded impedance matching design. Furthermore, the composite also demonstrates a high thermal storage capacity of 121.8 J g^−1^, excellent cycling stability with 97% performance retention over 300 cycles, and effective leakage resistance (Fig. 21a5). These combined properties indicate significant application potential in the field of thermal electromagnetic synergistic management for electronic devices. Regarding photothermal and electrothermal conversion, Hu et al. [235] successfully constructed a 3D network architecture of MoS_2_-decorated carbonized melamine foam and reduced graphene oxide, designated CMF/RGO/MoS_2_, via a method combining melamine foam carbonization with a hydrothermal reaction. The multifunctional phase change material was subsequently prepared by loading polyethylene glycol into this scaffold through vacuum impregnation (Fig. 21b1). The resulting composite achieved a high PEG loading capacity of 92.1 wt%. Microscopic analysis revealed an interpenetrating network formed by the RGO and the CMF skeleton, with MoS_2_ nanoparticles uniformly coating the surfaces (Fig. 21b2), which endowed the material with excellent anti-leakage properties (Fig. 21b3) and enhanced thermal performance. Specifically, the melting and crystallization enthalpies reached 169.3 and 165.9 J g^−1^, respectively. The material's multifunctionality is demonstrated in three key aspects. Firstly, it exhibits a photothermal conversion efficiency of 98.5%, originating from the synergistic broadband photon capture by RGO and MoS_2_ (Fig. 21b4). Secondly, it achieves an electrothermal conversion efficiency of 78.3%, attributed to the MoS_2_-enhanced conductive network (Fig. 21b5). Thirdly, it displays prominent EMA with a RL_min_ of −32.49 dB, a performance governed by mechanisms including multiple scattering within the 3D porous structure, dielectric loss, and interfacial polarization. Fourthly, it displays superior thermal response performance, heating to 50.1 °C within 300 s on a 55 °C hot stage, alongside a significantly improved thermal conductivity of 0.3549 W m^−1^ K^−1^, representing a 464% enhancement (Fig. 21b6). This design provides a novel strategy for developing advanced multifunctional phase change materials.

For thermal insulation and mechanical robustness, Guo et al. [165] constructed a MoS_2_/N-doped hollow carbon foam composite, designated MoS_2_/NCF, with multicomponent synergistic effects through the high-temperature pyrolysis of a melamine foam template combined with a hydrothermal reaction. During the synthesis process, the controlled precursor concentration enabled the in situ growth of 1T/2H mixed-phase MoS_2_, whose primary advantage lies in the synergistic enhancement of heterointerfacial polarization and defect-induced dipole polarization (Fig. 21c1). Microstructural analysis revealed that MoS_2_ nanosheets uniformly coat the 3D hollow neuro-like carbon skeleton (Fig. 21c2), forming a hierarchical conductive network. In thermal insulation performance tests, infrared thermography confirmed that a 7 mm thick sample could block a temperature rise of 110 °C from a 200 °C heat source (Fig. 21c3). The corresponding time–temperature profile indicated its low thermal conductivity of 0.0627 W m^−1^ K^−1^, which originates from the combined effects of phonon scattering by MoS_2_ and the localized thermal resistance of the N-doped carbon framework (Fig. 21c4). Regarding EMA performance, the material exhibited an ultra-wide EAB of 9.62 GHz and a RL_min_ of −41.07 dB at a thickness of 2.3 mm (Fig. 21c5). This is attributed to the synergistic interaction of interfacial polarization loss, conductive loss, and the impedance matching provided by the porous architecture, thereby realizing an integrated design for combined thermal management and EMA. In terms of mechanical flexibility and environmental robustness, Qian et al. [236] developed a novel EMW-assisted synthesis method for preparing FeCo@MoS_2_, a multicomponent TMDs composite. This technique utilizes an electromagnetic field to induce the exfoliation of MoS_2_ and facilitate the dispersed growth of FeCo nanoparticles, thereby enabling precise control over the material's architecture. The resulting FeCo@MoS_2_ polyvinyl alcohol aerogel exhibits remarkable compression resilience, withstanding up to 50% compressive strain, ultralight characteristics reflected by a specific surface area of 8.32 m^2^ g^−1^, and additional multifunctional properties including flame resistance and water repellency (Fig. 21d1). XRD patterns confirmed the successful formation of both the MoS_2_ (002) plane and the FeCo (110) crystalline phase (Fig. 21d2, d3). EMA evaluation revealed an optimal RL_min_ of −40.7 dB and an EAB of 6.4 GHz at a thickness of 2.5 mm (Fig. 21d4). The material maintains excellent impedance matching and attenuation balance even under deformation, achieved through the synergistic mechanism of an enhanced conductive network and magnetic loss. This work provides new insights for designing flexible EMA materials.

Multifunctional multicomponent TMDs composites achieve effective integration of EMA with additional functionalities through synergistic component interactions and precise interface regulation. The examples above cover thermal management with phase‑change energy storage, photothermal/electrothermal conversion, thermal insulation, mechanical flexibility, and environmental robustness. These functions are driven by practical application needs such as heat dissipation in electronics, adaptability for wearable devices, and durability in harsh environments. Their integration is typically achieved by incorporating functional secondary components and by designing hierarchical architectures. This system demonstrates a core advantage in transcending the limitations of single-material systems, maintaining excellent EMA while incorporating multiple functions such as thermal management, environmental responsiveness, and mechanical reinforcement, showcasing the distinctive features of integrated multifunctional design. Future research will focus on exploring dynamically tunable structures, adaptive interfaces, and intelligent response systems. Nevertheless, critical challenges including compatibility control in multicomponent systems, optimization of functional synergy, long-term stability assurance, and precise large-scale fabrication require thorough resolution. Addressing these issues will facilitate the transition of these advanced materials from fundamental research to practical engineering applications.

This chapter has systematically reviewed the multicomponent synergy strategies for TMDs-based EMW absorbers. Across carbon-based, other dielectric-based, magnetic-based, and multicomponent integrated systems, a central understanding emerges that optimal absorption performance does not rely on the extreme exploitation of a single component, but rather on the synergistic balance among different loss mechanisms. Carbon/TMDs composites offer the strongest conductive loss, yet excessive conductivity readily destroys impedance matching. Consequently, research has shifted from maximizing conductivity to finely tuning it through control over carbon dimensionality, content, and distribution. Dielectric-based composites (e.g., MXene, metal sulfides) primarily utilize heterointerfaces and defects to enhance polarization loss, achieving easier impedance matching but generally lower overall loss capability. The introduction of magnetic components compensates for the lack of magnetic loss in the low to medium frequency range, but it also brings new problems such as increased density and eddy current loss.

Notably, recent research trends have moved from double component composites toward multicomponent synergy and even multifunctional integration. This evolution reflects a deeper understanding of the nature of synergistic effects: different loss mechanisms are not simply additive, but can produce nonlinear enhancement through carefully designed interfaces and architectures. For instance, in a ternary carbon/magnetic/TMDs system, magnetic particles not only contribute magnetic loss but also improve impedance matching through electromagnetic interactions with the carbon network, while the locally distorted electric field further enhances interfacial polarization. Nevertheless, two major bottlenecks remain. First, the predictability of multicomponent systems is still poor, with most studies relying on trial and error and lacking unified design guidelines. Second, functional integration often comes with performance tradeoffs; how to achieve efficient multifunctional compatibility without compromising absorption performance remains a challenge. Therefore, future development of TMDs-based multicomponent absorbers urgently needs to shift from material screening to structural programming, with emphasis on cross scale precise construction, machine learning-assisted inverse design, and quantitative understanding of the coupling among multiple loss channels.

## Concluding Remarks and Future Perspectives

TMDs have demonstrated remarkable potential and extensive development prospects in the field of EMA, establishing themselves as one of the most promising dielectric EMA materials. Their unique 2D structure and intrinsic self-assembly characteristics endow them with inherent advantages for EMA applications. Notably, the TMDs family exhibits several key features: diverse cationic and anionic compositions, multiscale structure–property tunability spanning from the atomic to the sub-millimeter level, and excellent interfacial affinity and process compatibility for hybridization. These attributes make TMDs ideal 2D building blocks for hybrid materials, offering nearly infinite design possibilities for TMDs-based absorbers. This review begins by examining the compositional and structural characteristics of TMDs, with a focus on their multidimensional tunability. It first summarizes research on the multiscale regulation of dielectric and EMA properties in pure TMDs, highlighting their adjustable characteristics and EMW attenuation advantages as dielectric absorbers. Subsequently, the review discusses the performance of highly compatible TMDs in hybrid absorbers, emphasizing their capabilities in conductive and dielectric loss mechanisms. Recent advances in constructing conductive networks, optimizing impedance matching, achieving electromagnetic synergy, and enabling multifunctional compatibility are also addressed. Table 2 summarizes representative TMDs-based materials that exhibit outstanding broadband and high intensity EMA performance. Extensive studies confirm that TMDs composites possess excellent EMA properties, positioning them as highly promising candidates for next generation EMA materials.

**Table 2:** EMA performance of representative TMDs-based materials

	Materials	RL_min_ (dB)	*d* (mm)	EAB (GHz)	*d* (mm)	Loading (wt%)	Refrences
**Pure TMDs**							
Point defect	MoS_2_	−34.35	2.1	4.04	1.4	60	[89]
	Fe-MoS_2_	−72.18	1.65	5.36	1.95	60	[127]
	WVNbTaRu-MoS_2_	−61.2	–	7.65	2.09	40	[28]
Phase engineering	1T/2H MoS_2_	−59.8	2.68	6.8	2.48	55	[144]
	1T/2H MoS_2_	−56.32	1.85	5.88	2.13	60	[155]
	1T/2H MoS_2_	−45.5	3.5	3.89	2.0	50	[35]
	MoS_2_	−38.4	2.4	4.1	2.4	60	[20]
Morphology control	MoS_2_	−39.2	2.4	7.6	3.0	30	[153]
	MoS_2_	−47.8	2.2	5.2	1.9	60	[33]
	MoSe_2_	−57.2	2.7	5.6	2.05	50	[36]
	WS_2_	−63.0	2.5	4.6	2.5	30	[154]
	VS_2_ nanosheets	−57.4	5.75	7.0	-	40	[156]
**C/TMDs**							
1D	MoS_2_/CF	−43	2.7	8.75	2.7	5	[149]
	WS_2_/CF	−81.1	3.5	6.24	3.0	10	[170]
	MoS_2_@MWCNTs	−74.3	1.7	5.5	2.0	30	[23]
	WS_2_/CNT	−51.6	1.95	5.4	1.95	40	[172]
	MoS_2_@C nanowire	−52.56	2.4	6.0	2.3	25	[209]
2D	MoS_2_/RGO	−69.6	2.77	4.87	2.01	20	[175]
	MoSe_2_/RGO	−68.7	2.32	5.04	2.32	60	[78]
	MoSe_2_/GO	−52.08	1.8	5.3	1.8	10	[79]
	VSe_2_/RGO	−79.50	2.01	5.2	1.45	20	[176]
3D	MoS_2_/C	−75.94	1.68	4.68	1.65	25	[179]
	MoS_2_/C	−50.1	2.4	6.0	2.2	40	[181]
	VS_2_/C	−62.74	1.7	6.04	1.7	25	[37]
	MoS_2_/carbon foam	−45.88	2.2	5.62	2.2	40	[162]
	MoS_2_/CNF aerogel	−48.53	4.4	5.68	2.0	5	[163]
	MoS_2_/carbon foam	−41.07	2.2	9.62	2.3	–	[165]
**Other dielectric materials/TMDs**							
MXene	MoS_2_/MXene	−51.21	2.5	4.4	1.6	30	[185]
	MoS_2_/Ti_3_C_2_T_*x*_ aerogel	−61.65	4.53	5.9	2.0	3.9	[164]
	MoS_2_/Ti_3_C_2_T_*x*_ foam	−52.1	–	6.5	3.5	–	[187]
Metal sulfides	MoS_2_/NiS_2_	−41.05	2.2	4.4	2.2	20	[188]
	Cu_2-*x*_S@MoS_2_	−52.2	2.1	4.7	2.1	40	[189]
	MoS_2_/Ni_3_S_2_	−53.5	1.8	4.8	1.8	–	[190]
	MoS_2_/FeS_2_	60.2	2.0	6.48	2.0	50	[90]
Low-conductivity dielectric materials	MoS_2_/V_2_O_3_	−69.65	3.0	7.68	3.0	20	[27]
	MoS_2_/Pr_2_O_3_	−52.02	2.6	7.12	2.6	60	[34]
	MoS_2_/ZnO	−35.8	2.5	10.24	2.5	30	[194]
	MoS_2_/NiO	−53.31	4.3	4.88	2.22	40	[195]
	NbS_2_/Nb_2_O_5_	−43.85	–	6.48	2.07	40	[30]
	SiC_f_@MoS_2_	−72.76	2.86	7.7	2.84	15	[192]
	MoS_2_/MOF-A	−61.07	3.0	7.2	2.3	45	[145]
**Magnetic materials/TMDs**	Fe@MoS_2_	−37.02	2.0	4.73	2.0	60	[196]
	CoFe_2_O_4_@MoS_2_	−68.5	1.81	4.56	1.6	60	[200]
	ZnFe_2_O_4_@MoS_2_	−61.8	3.0	7.9	2.0	30	[203]
	MoS_2_/Fe_3_O_4_	−64.0	1.7	6.1	2.0	50	[204]
	MoS_2_/LiFe_5_O_8_	−63.61	2.18	5.36	2.50	–	[207]
Dimensional design	MoSe_2_/MoC/PNC	−59.09	1.9	6.96	1.9	27.5	[157]
	Co@C@MoS_2_	−61.97	1.9	5.6	2.1	15	[210]
	Cu/Cu_2_O-CF@MoS_2_	−56	4.0	5.18	2.1	40	[211]
Multicomponent composite strategy	CoFe_2_/C@MoS_2_	−66.8	2.1	5.3	2.1	30	[212]
	MoS_2_/PPy/RGO	−53.5	2.07	5.36	1.82	30	[219]
	MoS_2_/CoS_2_/VN	−50.48	2.0	5.76	2.0	40	[220]
	Fe/Fe_3_O_4_@C@MoS_2_	−36.1	4.0	5.4	1.8	60	[223]
	Fe@C@TiO_2_@MoS_2_	−54.2	2.5	9.6	2.0	60	[224]
	CoNi_2_S_4_/Co_9_S_8_@SiO_2_@MoS_2_	−58.31	2.49	6.4	2.03	40	[225]
Core–shell engineering	Fe_3_O_4_@C@MoS_2_	−68.9	4.1	5.25	2.0	50	[226]
	Co_9_S_8_/CNT/MoS_2_	−35.4	3.8	8.4	3.8	9	[228]
	Fe_3_O_4_/CF/MoS_2_	−70.0	2.5	4.68	2.5	40	[230]
**Multifunctional composites**	MXene/CNT@MoS_2_	−64.1	2.1	4.28	2.1	66	[234]
	PEG/CMF/RGO/MoS_2_	−32.49	2.1	6.16	2.4	–	[235]
	FeCo@MoS_2_/PVA	−40.7	2.5	6.4	2.5	50	[236]

From the body of this review, several core design principles can be distilled. First, constructing conductive networks through carbon based or metallic components effectively enhances conductive loss, but must be carefully balanced with impedance matching. Second, engineering abundant heterointerfaces (e.g., core shell structures, MOF derived hybrids) significantly boosts interfacial polarization. Third, introducing defects such as sulfur vacancies or phase boundaries promotes dipole polarization and relaxation loss. Fourth, magnetic dielectric synergy, while improving impedance matching, often introduces eddy current losses at high frequencies, requiring optimization of magnetic phase content and distribution.

Regarding mechanistic consensus, it is now widely accepted that the dielectric loss of TMDs-based absorbers originates from multiple contributions: conductive loss from interconnected networks, dipolar polarization from intrinsic defects (vacancies, edges, intercalants), and interfacial polarization from phase interface and heterointerfaces between TMDs and other components. Magnetic loss, when present, arises solely from added magnetic phases, as TMDs themselves are nonmagnetic. However, the quantitative separation of these loss channels and the establishment of clear structure–property relationships remain incomplete.

A critical summary of the current research status reveals that while numerous studies have reported impressive EMA performance, most remain at the level of case specific demonstrations without systematic comparison under standardized conditions. Moreover, many works rely on empirical optimization rather than mechanistic prediction, and the reproducibility of reported results is often not verified. Therefore, the field would benefit from more rigorous benchmarking, shared databases, and the adoption of machine learning approaches to accelerate rational design. Several critical challenges remain unresolved, and further investigation in the following areas would be valuable. (1) Precise composition and structure regulation and advanced characterization; (2) multiscale synergistic engineering and inverse intelligent design; (3) low cost and sustainable manufacturing; (4) multifunctional integration and intelligent response:

(i) More effective methods for regulating the composition and structure of TMDs, along with advanced characterization techniques, are imperative. In fields such as semiconductor devices, electrocatalysis, and energy storage, strategies including substitution or intercalation doping, vacancy engineering, and precise phase transition in TMDs have been extensively studied. Sophisticated equipment, such as numerically controlled CVD systems, irradiation and plasma processing systems, and spherical aberration corrected TEM with in situ capabilities, has enabled atomic level structural analysis. However, these approaches have not yet been widely or systematically applied in EMA research. For example, emerging mechanisms such as Mo vacancy engineering [237], intercalation regulation [118] and TMDs moiré superlattices [238, 239] for regulating dielectric and EMA properties have not yet been reported. Although numerous studies on TMDs-based absorbers have been reported, the underlying mechanisms through which the composition and structure of TMDs influence their electronic properties, and consequently their dielectric and EMA performance, remain insufficiently investigated. Leveraging interdisciplinary knowledge of TMDs to achieve complementary advantages and to clarify how atomic scale structures govern electromagnetic energy dissipation represents a critical direction for future research. Such progress is fundamental for advancing TMDs as a primary class of EMA materials.
(ii) Multiscale and multicomponent synergistic regulation combined with inverse intelligent design represents a crucial research direction. Future advances will transcend conventional trial and error approaches by establishing a new paradigm that integrates precise multiscale control of material systems with artificial intelligence. Achieving multiscale synergy requires systematic analysis of how different structural hierarchies from atomic doping and vacancies to heterointerfaces and macroscopic 3D networks collectively influence electromagnetic parameters [240]. Through precisely constructed multivariate heterojunctions and complex topological architectures, researchers can achieve effective coordination between dielectric and magnetic losses while maintaining optimal impedance matching. Inverse intelligent design constitutes a paradigm shift centered on using machine learning to establish accurate mapping models between material composition structure and functional performance [241]. This approach enables researchers to input target performance metrics and computationally identify optimal solutions from vast compositional spaces [242, 243]. Consequently, it facilitates rational and efficient design of high performance composite materials while significantly accelerating the development cycle for new EMA materials.
(iii) Low-cost, large-scale synthesis and green manufacturing represent critical challenges for transitioning TMDs from laboratory research to industrial applications in the field of EMA. The primary obstacle lies in overcoming the limitations of current preparation methods in terms of cost, production scale, and environmental impact. Future opportunities reside in developing disruptive synthetic pathways based on novel, inexpensive precursors, such as low-toxicity sulfur sources or metal salts, to achieve efficient conversion through mild liquid-phase methods or modified CVD techniques. Concurrently, it is essential to innovate engineering processes by developing continuous-flow reactors or moving-bed deposition systems to replace inefficient batch processing modes, thereby enabling stable, large-scale production at the kilogram level or beyond. Furthermore, exploring green manufacturing routes with low energy consumption and minimal emissions, such as electrochemical and mechanochemical methods, is of great importance for TMDs-based absorbers. Only by establishing fully integrated green processes spanning from raw materials to final products can a solid material foundation be laid for the broad application of TMDs at macroscopic scales, ultimately achieving the transformation from high-end laboratory samples to bulk industrial materials.
(iv) Multifunctional integration and intelligent response. The sole capacity for EMA has become inadequate to satisfy the advanced requirements of modern electronic systems for multifunctional integration. The field is consequently evolving from static absorbers toward dynamic intelligent systems. Multifunctional integration aims to achieve a deep combination of EMA capability with other physical or chemical functions. Examples include developing material systems that integrate efficient EMW dissipation with superior thermal management properties, mechanical strength, microwave/terahertz/infrared compatible stealth technology, or fabricating integrated devices capable of simultaneous energy harvesting and stress sensing. This paradigm transforms EMW from a passive burden into an actively utilized resource. Intelligent response, meanwhile, seeks to endow materials with the ability to transcend static properties. It enables their EMA performance to adapt autonomously in response to external environmental stimuli, such as temperature, humidity, or electric fields [244]. Realizing this requires the incorporation of stimuli-responsive molecules or phase-change materials to construct dynamically controllable microstructures and interfaces. The ultimate goal is to create a new generation of materials capable of intelligently switching between stealth and communication states under external field control, thereby providing the fundamental material basis for adaptive camouflage and next generation intelligent devices.

## Abbreviations

EMW: Electromagnetic wave
EMA: Electromagnetic wave absorption
RL_min_: Minimum reflection loss value
EAB: Effective absorption bandwidth
1D: One-dimensional
2D: Two-dimensional
3D: Three-dimensional
*ε*_*r*_: Complex relative permittivity
*μ*_*r*_: Complex relative permeability
$\varepsilon_{p}^{\prime \prime }$: Polarization loss part
$\varepsilon_{c}^{\prime \prime }$: Conductive loss part
*α*: Attenuation constant
DFT: Density functional theory
CBM: Conduction band minimum
VBM: Valence band maximum
*Β*: Correction factor
CVD: Chemical vapor deposition
XRD: X-ray diffraction
SEM: Scanning electron microscope
TEM: Transmission electron microscope
XPS: X-ray photoelectron spectroscopy
HAADF-STEM: High-angle annular dark-field scanning transmission electron microscope
